# Microglia activation by derepression of endogenous retroviruses drives inflammation and cellular senescence

**DOI:** 10.1038/s41593-026-02404-y

**Published:** 2026-08-17

**Authors:** Xin Yan, Christina Georgopoulou, Hang-Mao Lee, Ala Ahrari, Jenny Russ, Vijay Chandrasekar, Tim Ducksch, Giuliano Crispatzu, Valentina Talevi, Liang Qiao, Shobhit Agrawal, Sophie Crux, Andrew W. Daman, Lena Wischhof, Miriam Stork, Margit Zweyer, Emma Dorotea Zanfi, Manon Chevallot-Beroux, Yunxiao Li, Elena De-Domenico, Dina Hüsson, Lorenzo Bonaguro, Yuanfang Li, Jonas Schulte-Schrepping, Qingyi Liang, Ketty Kessler, Dan Ehninger, Shahin Rafii, Jiankai Luo, Andreas Hermann, Annett Halle, Ying Liu, Elvira Mass, Melania Capasso, Hiroki Kato, Joachim L. Schultze, Pierluigi Nicotera, Daniele Bano, Steven Zvi Josefowicz, Martin Fuhrmann, Thomas Ulas, Marc Beyer, Juntang Lin, Monique M. B. Breteler, N. Ahmad Aziz, Paolo Salomoni

**Affiliations:** 1 Nuclear Function Group, German Center for Neurodegenerative Diseases (DZNE), Bonn, Germany; 2 Translational Neurodegeneration Section ‘Albrecht Kossel’, University Medical Center Rostock, University of Rostock, Rostock, Germany; 3 Population Health Sciences, DZNE and Institute for Medical Biometry, Informatics and Epidemiology (IMBIE), Faculty of Medicine, University of Bonn, Bonn, Germany; 4 Collaborative Innovation Center of Stem Cell and Biotherapy, Henan Medical University, Xinxiang, China; 5 Henan Joint International Research Laboratory of Stem Cell Medicine, Henan Medical University, Xinxiang, China; 6 Platform for Single Cell Genomics and Epigenomics (PRECISE), DZNE and University of Bonn, Bonn, Germany; 7 Immunogenomic and Neurodegeneration Group, DZNE, Bonn, Germany; 8 Neuroimmunology and Imaging Group, DZNE, Bonn, Germany; 9 Immunology and Microbial Pathogenesis Program, Department of Pathology and Laboratory Medicine, Division of Cell and Cancer Pathobiology, Weill Cornell Medicine Graduate School of Medical Sciences, New York, NY USA; 10 Aging and Neurodegeneration Group, DZNE, Bonn, Germany; 11 Clinical Single Cell Omics, Systems Medicine, DZNE, Bonn, Germany; 12 Department of Cancer Biology, University College London (UCL) Cancer Institute, University College London, London, UK; 13 Translational Biogerontology Group, DZNE, Bonn, Germany; 14 Division of Regenerative Medicine, Hartman Institute for Therapeutic Organ Regeneration, Ansary Stem Cell Institute, Department of Medicine, Weill Cornell Medicine, New York, NY USA; 15 German Center for Neurodegenerative Diseases (DZNE), Rostock/Greifswald, Rostock, Germany; 16 Microglia and Neuroinflammation, DZNE, Bonn, Germany; 17 Department of Neuropathology, University Hospital Bonn, Bonn, Germany; 18 Department of Surgery, Weill Cornell Medicine, New York, NY USA; 19 Life and Medical Sciences (LIMES) Institute, Developmental Biology of the Immune System, University of Bonn, Bonn, Germany; 20 Immune Regulation Group, DZNE, Bonn, Germany; 21 Institute of Cardiovascular Immunology, University Hospital Bonn, University of Bonn, Bonn, Germany; 22 Genomics and Immunoregulation, Life and Medical Sciences (LIMES) Institute, University of Bonn, Bonn, Germany; 23 Synaptic Connectivity and Neurodegeneration Group, DZNE, Bonn, Germany; 24 Academy of Chinese Medical Sciences, Henan University of Chinese Medicine, Zhengzhou, China; 25 Institute for Medical Biometry, Informatics and Epidemiology (IMBIE), Faculty of Medicine, University of Bonn, Bonn, Germany; 26 Department of Neurology, Faculty of Medicine, University of Bonn, Bonn, Germany

**Keywords:** Microglia, Histone variants

## Abstract

Aging-associated loss of chromatin compaction is linked to derepression of retrotransposable elements (RTEs) in mouse and human tissues. Whether such RTE transcription contributes to the microglia activation that is common in aged brains is unknown. Here, we show that DAXX, a histone chaperone and RTE repressor, is downregulated during aging, preserves microglia homeostasis and inhibits cellular senescence. Loss of *Daxx* in young-adult microglia drives a reactive phenotype marked by chromatin decompaction at RTEs, loss of homeostatic markers, cell cycle re-entry and behavioral changes. This state leads to DNA damage and microglial depletion, followed by replacement with DAXX-deficient/*Apoe*^high^ microglia displaying features of senescence. Sustained induction of senescence relies on promyelocytic leukemia protein, a DAXX-interacting factor and interferon target. Together, these findings highlight the importance of heterochromatin maintenance in preserving adult microglial identity and plasticity, with broader implications for brain homeostasis, healthy aging and behavior.

## Abstract (web summary)

Reawakening of endogenous retroviruses due to loss of chromatin compaction causes microglia to acquire aging-associated features, leading to perturbed tissue homeostasis and behavior.

## Main

Loss of heterochromatin is a hallmark of aging and cellular senescence^1^ and has been linked to derepression of repeat elements, including retrotransposable elements (RTEs)^2–4^. In adult tissues, RTEs are silenced via multiple mechanisms, including loading of the histone variant H3.3 (refs. ^5,6^). RTEs are co-opted in the response to inflammatory stimuli via their enhancer function and are themselves targets of key proinflammatory transcription factors (TFs)^7^. More generally, RTE deregulation has been implicated in aging, neurodegenerative diseases, autoinflammatory conditions and cancer^5,8–10^.

Microglia are the resident innate immune cells of the CNS^11^, where they mediate surveillance against infections and various brain pathological states^12–14^. During aging and in neurodegenerative conditions, microglia undergo transition from a homeostatic state to distinct reactive states^15,16^, which have been reported as either beneficial or detrimental depending on the disease type and/or stage. These transitions are associated with profound alterations of the microglia transcriptome and their function^16,17^. Although chromatin modifications have been suggested to modulate microglia development and activity^18,19^, the impact of age-dependent decline in chromatin compaction in microglia remains mostly unexplored. In this respect, trimethylated lysine 27 histone 3 (H3K27me3) was linked to either an increased or decreased inflammatory response^20–22^. Interestingly, loss of heterochromatin has been associated with senescence induced by pathogenic Tau^23^ and to the ‘dark microglia’ state^24^.

The H3.3 chaperone DAXX^25^ acts as a repressor of RTEs of viral origin (hereafter referred to as long-terminal repeat RTEs (LTR-RTEs))^4,26–28^ via interaction with H3.3 and the H3K9 methyltransferase TRIM28–SETDB1 complex^4^. We have shown that loss of *Daxx* in hematopoietic progenitors causes a reduction in H3K9me3 and RTE derepression, leading to PU.1-mediated neutrophilia^27^. Moreover, DAXX is upregulated in microglia following interferon-γ (IFNγ) stimulation^29^, but the functional implications of its induction remain unknown.

In the present study, we unravel a role for DAXX in maintenance of the homeostatic state of adult microglia, with implications for our understanding of the relationship between loss of epigenetic barriers, innate immune cell senescence and aging-associated neuroinflammation.

## Results

### Expression dynamics of RTEs and their regulators during maturation of microglia from development to adult stages

**Fig. 1:**
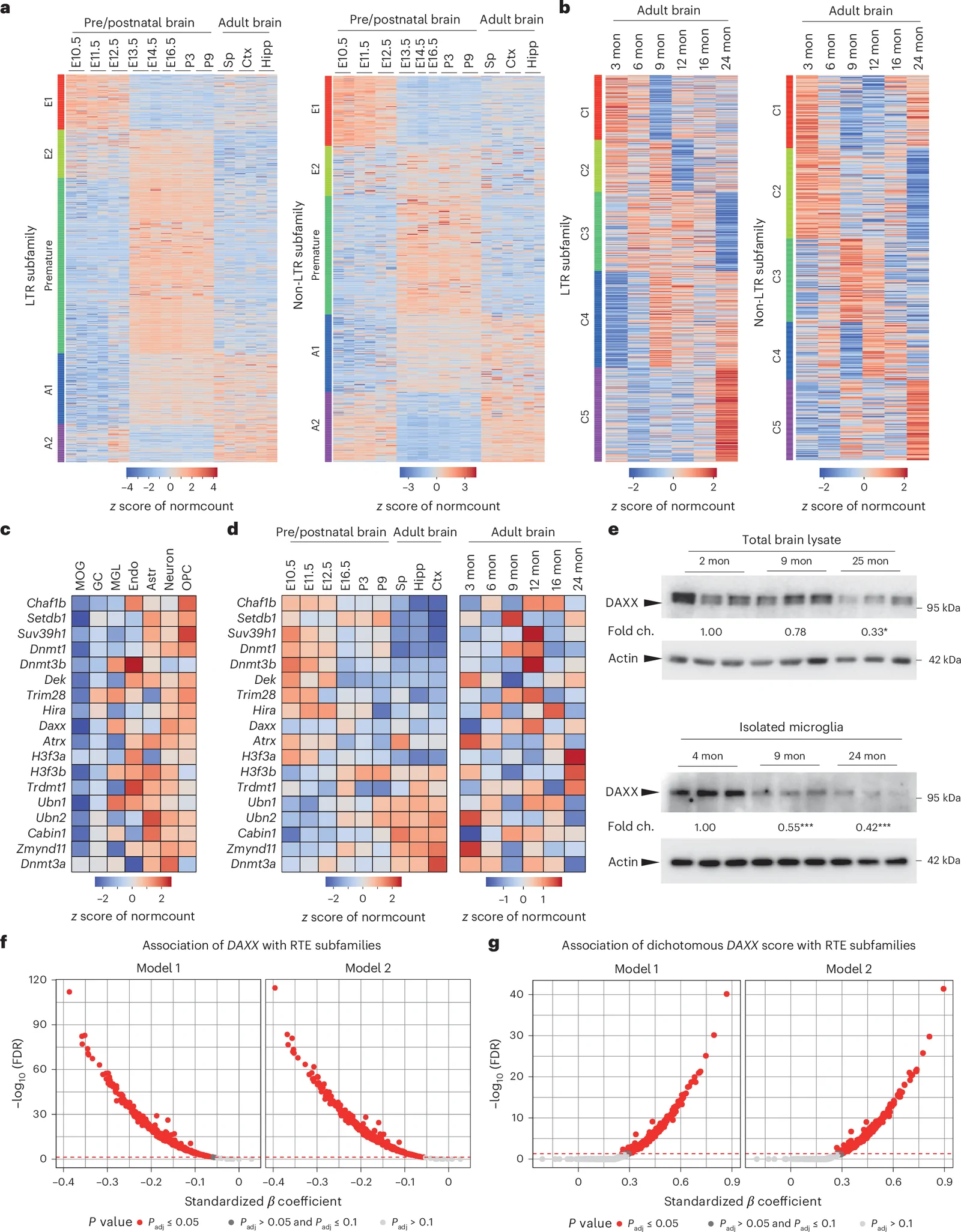
Dynamic expression of RTEs and RTE regulators in microglia during development, in the adult brain and during aging. **a**, Heat maps showing *k*-means clustering (*k* = 5) of dynamically expressed LTRs and non-LTRs in microglia isolated from the developing brain or CNS across embryonic, postnatal and adult stages (data from ref. ^30^; *n* = 1 mouse for E14.5 and E16.5, *n* = 2 mice per group for the rest). Clusters are named as early microglia cluster 1 (E1), E2, premature, adult microglia cluster 1 (A1) and A2 and are indicated on the left; mon, month; normcount, normalized count. **b**, Heat maps showing *k-*means clustering (*k* = 5) of dynamically expressed LTRs and non-LTRs in microglia isolated from 3- to 24-month-old mouse brains (data from ref. ^33^; *n* = 4 mice for 3 and 12 months, *n* = 2 mice for 6 months, *n* = 3 mice for 9 and 24 months, *n* = 5 mice for 16 months). Clusters are named as C1, C2, C3, C4 and C5 and are indicated on the left. **c**, Heat map showing the relative expression of RTE repressor genes across major brain cell types (data from ref. ^34^). **d**, Heat maps showing the relative expression of RTE repressor genes in microglia across embryonic (E), postnatal (P), adult and aging stages (data from refs. ^30,33^). **e**, Western blots and quantification analysis of DAXX expression in whole-brain lysates from 2-, 9- and 25-month-old mice (*n* = 3 mice for each age; data were analyzed by ordinary one-way analysis of variance (ANOVA), *F* = 5.812, *P* = 0.0395; Tukey’s multiple comparisons test, *P*_9 month versus 2 month_ = 0.5353, *P*_25 month versus 2 month_ = 0.0355, *P*_25 month versus 9 month_ = 0.1449) and in isolated microglia from brains of 4-, 9- and 24-month-old mice (*n* = 3 mice for each age; data were analyzed by ordinary one-way ANOVA, *F* = 57.81, *P* = 0.0001; Tukey’s multiple comparisons test, *P*_9 month versus 4 month_ = 0.0005; *P*_24 month versus 4 month_ = 0.0001; *P*_24 month versus 9 month_ = 0.1213). β-Actin served as a loading control; Fold ch., fold change. **f**, Volcano plots showing the association between expression levels of *DAXX* (on a continuous scale) and those of RTE subfamilies (*N* = 2,922) in a human cohort using multivariable linear regression models, while applying false discovery rate (FDR) correction to account for multiple comparisons; Model 1 (adjusted for age and sex): higher expression levels of *DAXX* were associated with lower expression levels of 782 (of 795) RTE subfamilies (FDR-adjusted two-sided *P* < 0.05); Model 2 (adjusted for age, sex and white and red blood cell counts): higher expression levels of *DAXX* were associated with lower expression levels of 785 (of 795) RTE subfamilies (FDR-adjusted two-sided *P* < 0.05). Each dot represents one RTE subfamily, with statistically significant associations (that is, FDR-adjusted two-sided *P* < 0.05) marked in red. The *x* axis shows the regression coefficients for the association of *DAXX* with each RTE subfamily, while the dashed red line represents the threshold for statistical significance. **g**, Volcano plots showing the association between expression levels of *DAXX* (categorized into low-*DAXX* versus high-*DAXX* groups based on the expression levels of *DAXX* falling within the 20% lowest or highest range of the population distribution, respectively; *N* = 1,170), using multivariable linear regression and applying FDR correction to account for multiple comparisons; Model 1 (adjusted for age and sex): the expression levels of 423 RTE subfamilies were higher in the low-*DAXX* group than in the high-*DAXX* group (FDR-adjusted two-sided *P* < 0.05); Model 2 (adjusted for age, sex and white and red blood cell counts): the expression levels of 467 RTE subfamilies were higher in the low-*DAXX* group than in the high-*DAXX* group (FDR-adjusted two-sided *P* < 0.05). Each dot represents one RTE subfamily, with statistically significant associations (that is, FDR-adjusted two-sided *P* < 0.05) marked in red. The *x* axis shows the regression coefficients for the association between *DAXX* expression group and each RTE subfamily, while the dashed red line represents the threshold for statistical significance. Abbreviations: Astr, astrocyte; Ctx, cortex; Endo, endothelial cells; GC, glial cells; Hipp, hippocampus; MGL, microglia; MOG, mature oligodendrocyte; OPC, oligodendrocyte progenitor cells; Sp, spinal cord. Data are presented as mean ± s.e.m. Asterisks (*) indicate statistical significance: *P < 0.05 and ***P < 0.001; *P*_adj_, adjusted *P* value. Source data

We sought to investigate the expression of RTEs and their regulators during development and in the adult brain. Microglia originate from yolk sac progenitors, which colonize the CNS and differentiate into specialized resident macrophages^11^. By mining existing RNA sequencing (RNA-seq) datasets^30^ and *k*-mean clustering analysis, we revealed dynamic regulation of LTR- and non-LTR-RTEs across developmental and adult stages (Fig. 1a). While the early-stage cluster E1 displayed higher RTE expression, the E2 cluster was enriched in both early and fetal microglia but was downregulated in adult microglia. By contrast, the adult microglia clusters (A1–A2) were enriched in both embryonic and adult microglia. In accordance with previous studies showing RTE deregulation during aging^31,32^, a number of LTR-RTE subfamilies (based on ref. ^33^) were enriched in 24-month-old microglia (Fig. 1b).

We then assessed the expression of RTE regulators and associated factors across different brain cell types^30,34^ (Fig. 1c). The expression of *H3f3a* was higher at earlier stages, whereas *H3f3b* expression peaked at perinatal and adult stages until 3 months old (Fig. 1d). Both *H3f3a* and *H3f3b* were upregulated in 24-month-old microglia, in accordance with the known increase in H3.3 deposition at euchromatin in postmitotic cells in aged mice^35,36^. Despite the slight increase in *Daxx* mRNA expression in 24-month-old microglia (Fig. 1d), DAXX protein levels were gradually downregulated in whole-brain lysates and in column-isolated microglia during aging (Fig. 1e), suggesting that DAXX is post-transcriptionally regulated, as previously proposed^4,27^. Finally, other RTE repressors and heterochromatin regulators, such as *Suv39h*, *Dnmt3b*, *Setdb1* and *Trim28*, were enriched in early and premature microglia (Fig. 1d), whereas adult microglia displayed higher *Dnmt3a* and *Trdmt1* expression, suggesting dynamic involvement of different RTE-silencing mechanisms during microglia maturation.

**Extended Data Fig. 1:**
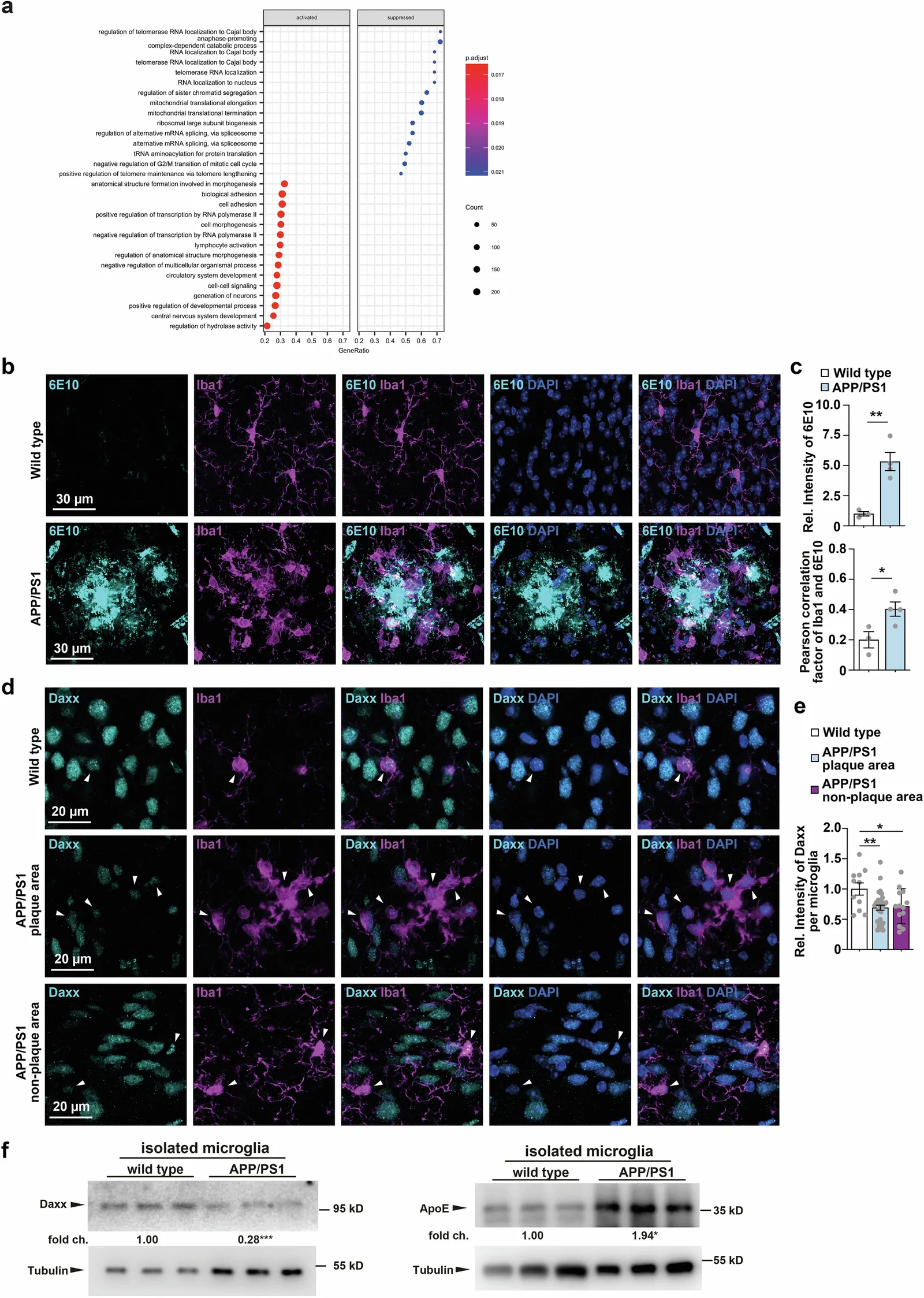
Assessing correlation between Daxx and gene expression in human blood cells and its protein levels in an AD model. **a**, Gene ontology analysis for the correlation of *DAXX* expression with the expression levels of 12167 other genes measured in the Rhineland Study (*N* = 2966): all genes were first ranked in descending order according to the Spearman’s correlation of their expression levels with that of *DAXX*, followed by a rank-based gene set enrichment analysis, applying false discovery rate (FDR) correction to account for multiple comparisons (*P*-values are two-sided and based on a permutation test using a weighted Kolmogorov-Smirnov-like statistic implemented in the clusterProfiler R package v. 3.18.1). **b-c**, Representative confocal images of Iba1 and 6E10 immunostaining and quantification of 6E10 intensity in the cortex of 12-month-old APP/PS mice (*n* = 4 mice) and wild type controls (*n* = 3 mice) (comparison by two-tailed unpaired Student’s *t* test, *P*_6E10_ = 0.005, *P*_Pearson correlation_ = 0.0364). **d**, **e**, Representative confocal images of Iba1 and Daxx immunostaining and quantification of Daxx intensity in the Aβ plaque^+^ and non^−^plaque-associated microglia from 12-month-old APP/PS mice (*n* = 36 and 13 cells, respectively, from 4 mice) and microglia from age-matched wild type controls (*n* = 11 cells from 3 mice) (Comparison by ordinary one-way ANOVA, *F* = 5.462, *P* = 0.0068; Sidak’s multiple comparisons test, *P*_plaque+ area vs WT_ = 0.0057; *P*_non-plaque area vs WT_ = 0.0491). **f**, Western blots and quantification of Daxx and ApoE in microglia isolated from 12-month-old APP/PS1 mice and wild type controls (*n* = 3 mice per group, comparison by two-tailed unpaired Student’s *t* test, *P*_Daxx_ < 0.0001, *P*_ApoE_ = 0.0495). Data are presented as mean ± s.e.m. Asterisks (*) indicate statistical significance: *P < 0.05, **P < 0.01 and ***P < 0.001; n.s. indicates not significant. Source data

We recently showed an association between RTE expression and aging in blood cells from the Rhineland Study^37^, a prospective study of healthy individuals during aging. We mined the same dataset to show that *DAXX* levels were associated with lower expression of nearly all RTE subfamilies, also after accounting for blood cell counts (Fig. 1f). The sensitivity analysis, conducted using a dichotomous *DAXX* score in a subset of the Rhineland Study population (*N* = 1,170) supported these results (Fig. 1g). A rank-based gene set enrichment analysis revealed that genes whose expression levels were positively correlated with *DAXX* expression were negatively associated with terms related to translation and RNA processing (Extended Data Fig. 1a). These data demonstrate an inverse correlation between *DAXX* expression and RTEs in humans.

Finally, we examined whether DAXX expression was affected in a model of Alzheimer’s disease (AD). To this end, we performed immunostaining on brain sections from 12-month-old APP/PS1 mice, where 6E10^+^ amyloid-β plaques in the cortex are surrounded by IBA1^+^ microglia (Extended Data Fig. 1b,c). DAXX intensity was reduced in microglia in both plaque and non-plaque areas, with the former showing a more marked reduction (Extended Data Fig. 1d,e). Accordingly, western blot analysis showed diminished DAXX protein levels, inversely correlating with the disease-associated microglia (DAM) marker ApoE (Extended Data Fig. 1f).

Together, these findings reveal dynamic modulation of RTEs and their regulators during brain development and aging and identify DAXX as an important RTE regulator in both human and mouse tissues during aging and in brain pathological states.

### DAXX represses RTEs and IFN-stimulated genes at steady state and after inflammatory stimuli in microglia ex vivo

**Extended Data Fig. 2:**
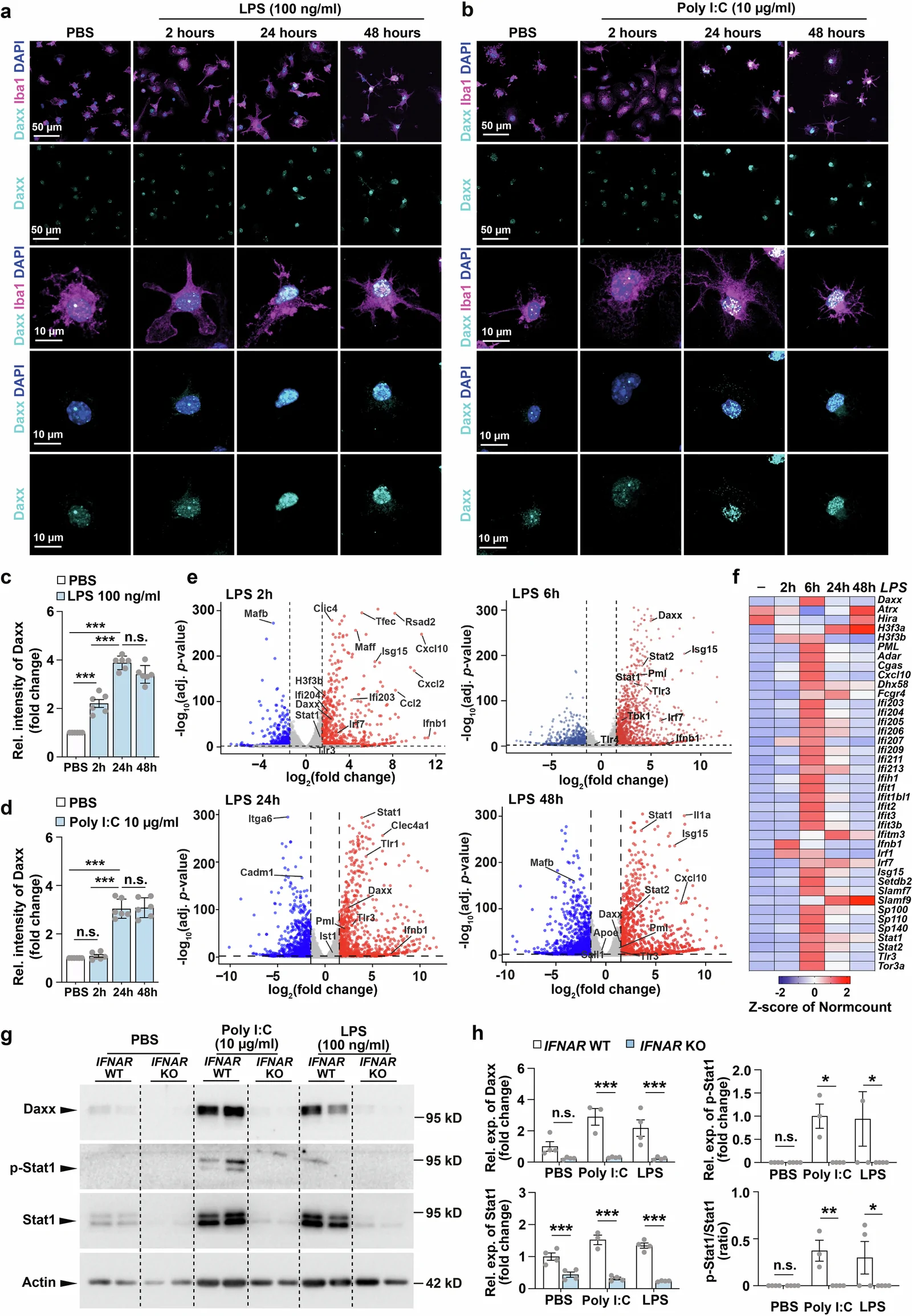
Stimulation with LPS or Poly I:C increases Daxx expression in microglia and BMDMs *ex vivo*. **a**, **c**, Representative confocal images of Daxx and Iba1 immunostaining and quantification of Daxx intensity in primary microglia following PBS or 100 ng/mL LPS stimulation for 2 h, 24 h and 48 h (*n* = 6 independent preparations per group, comparison by ordinary one-way ANOVA, *F* = 108.3, *P* < 0.0001; Tukey’s multiple comparisons test, *P*_PBS vs LPS 2h_ < 0.0001, *P*_PBS vs LPS 24h_ < 0.0001, *P*_LPS 2h vs LPS 24h_ < 0.0001, *P*_LPS 24h vs LPS 48h_ = 0.0608). **b**, **d**, Representative confocal images of Daxx and Iba1 immunostaining and quantification of Daxx protein intensity in primary cultured microglia upon PBS or 10 µg/mL Poly I:C stimulation for 2 h, 24 h and 48 h (*n* = 6 independent preparations per group, comparison by ordinary one-way ANOVA, *F* = 94.36, *P* < 0.0001, Tukey’s multiple comparisons test, *P* values: *P*_PBS vs PolyI:C 2h_ = 0.9546, *P*_PBS vs PolyI:C 24h_ < 0.0001, *P*_PolyI:C 2h vs PolyI:C 24h_ < 0.0001, *P*_PolyI:C 24h vs PolyI:C 48h_ = 0.9937). **e**, Volcano plots showing differentially expressed genes in primary microglia isolated from wild type mice following 2h-, 6h-, 24h- and 48h-LPS stimulation relative to PBS-treated controls. Differential expression analysis was performed using two-sided Wald tests implemented in DESeq2 with Benjamini–Hochberg correction. Dotted lines indicate fold-change and adjusted *P*-value thresholds. **f**, Heatmap representing expression of RTE repressor and interferon-response genes in primary microglia after LPS stimulation. **g**, **h**, Western blots and quantification of Daxx, p-Stat1, Stat1 in BMDMs from *interferon alpha receptor* knockout (*IFNAR* KO) mice and control mice (*IFNAR* WT) following 6 h Poly I:C or LPS stimulation. PBS: *n* = 4 independent preparations for each genotype; Poly I:C: *n* = 3 independent preparations for *IFNAR* WT mice and *n* = 4 independent preparations for *IFNAR* KO mice; LPS: *n* = 4 independent preparations per group (Comparison by ordinary two-way ANOVA with Sidak’s multiple comparisons test: for Daxx, *F* = 4.449, *P*_PBS IFNAR WT vs IFNAR KO_ = 0.0919, *P*_PolyI:C IFNAR WT vs IFNAR KO_ < 0.0001, *P*_LPS IFNAR WT vs IFNAR KO_ = 0.0919; for Stat1, *F* = 8.321, *P*_PBS IFNAR WT vs FNAR KO_ = 0.0009, *P*_PolyI:C IFNAR WT vs IFNAR KO_ < 0.0001, *P*_LPS IFNAR WT vs IFNAR KO_ < 0.0001; for p-Stat1, *F* = 2.279, *P*_PBS IFNAR WT vs IFNAR KO_ > 0.9999, *P*_PolyI:C IFNAR WT vs IFNAR KO_ = 0.0216, *P*_LPS IFNAR WT vs IFNAR KO_ = 0.0199; for p-Stat1/Stat1, *F* = 2.907, *P*_PBS IFNAR WT vs IFNAR KO_ > 0.9999, *P*_PolyI:C IFNAR WT vs IFNAR KO_ = 0.0072, *P*_LPS IFNAR WT vs IFNAR KO_ = 0.0174). Data are presented as mean ± s.e.m. Asterisks (*) indicate statistical significance: *P < 0.05, **P < 0.01 and ***P < 0.001; n.s. indicates not significant. Source data

We next decided to investigate DAXX expression and regulation in microglia. In agreement with previous studies showing its induction following treatment with type I or II IFNs in cell lines^38^, we showed that both lipopolysaccharide (LPS) and polyinosinic:polycytidylic acid (poly(I:C)) elevated DAXX protein levels in primary microglia, peaking at late time points (Extended Data Fig. 2a–d). Furthermore, RNA-seq data from primary microglia showed marked LPS-induced upregulation of *Daxx* expression at 6 h, in conjunction with other inflammatory and IFN-stimulated genes (ISGs), including *Stat1*, *Ifnb1*, *Isg15* and *Irf7* (Extended Data Fig. 2e,f). Finally, by using bone marrow-derived macrophages (BMDMs) from IFNα receptor knockout (*Ifnar*-KO) mice, we showed that DAXX induction is dependent on IFN signaling (Extended Data Fig. 2g,h).

**Fig. 2:**
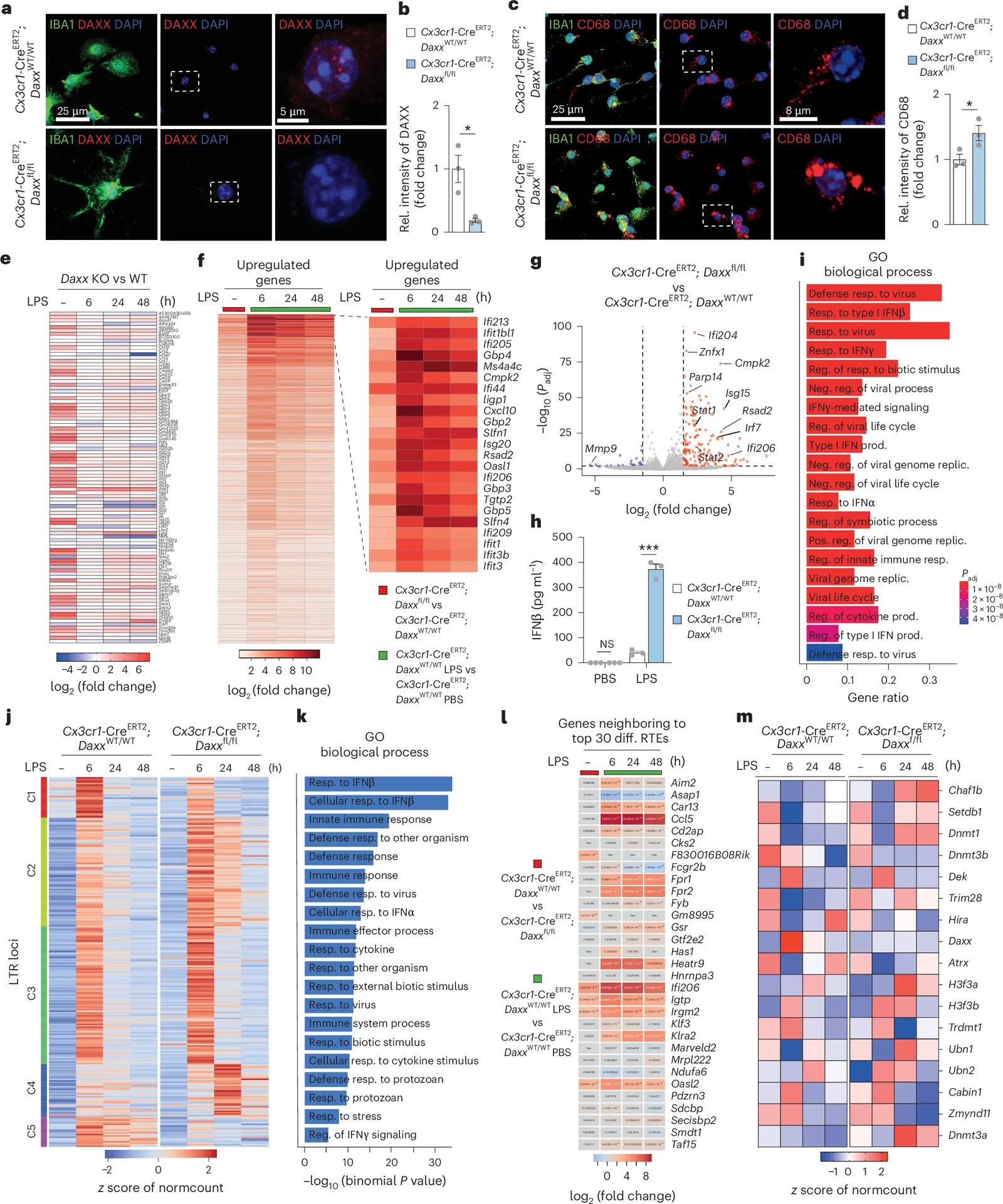
Loss of *Daxx* in primary microglia triggers RTE activation and ISGs at steady state and prolongs expression upon stimulus. **a**,**b**, Representative confocal images of IBA1, DAXX and DAPI immunostaining (**a**) and quantification (**b**) of DAXX intensity in primary microglia isolated from *Cx3cr1*-Cre^ERT2^; *Daxx*^fl/fl^ and *Cx3cr1*-Cre^ERT2^; *Daxx*^WT/WT^ mice (*n* = 3 independent preparations per genotype; data were analyzed by two-tailed unpaired Student’s *t*-test, *P* = 0.02); Rel., relative. **c**,**d**, Representative confocal images of IBA1, CD68 and DAPI immunostaining (**c**) and quantification (**d**) of CD68 intensity in primary microglia isolated from *Cx3cr1*-Cre^ERT2^; *Daxx*^fl/fl^ and *Cx3cr1*-Cre^ERT2^; *Daxx*^WT/WT^ mice (*n* = 3 independent preparations per genotype; data were analyzed by two-tailed unpaired Student’s *t*-test, *P* = 0.0467). **e**, Heat map representing the top 100 differentially expressed IFN pathway genes in DAXX-deficient and control primary microglia under basal conditions or following 6-, 24- and 48-h LPS stimulation. **f**, Left, heat map representing the overlap between genes upregulated in DAXX-deficient microglia at steady state and genes induced by LPS stimulation in control microglia. Right, selected inflammatory genes from the overlapping gene set. **g**, Volcano plot showing differentially expressed genes in *Cx3cr1*-Cre^ERT2^; *Daxx*^fl/fl^ versus *Cx3cr1*-Cre^ERT2^; *Daxx*^WT/WT^ primary microglia. Differential expression analysis was performed using DESeq2 with a two-sided Wald test. *P* values were adjusted for multiple testing using the Benjamini–Hochberg procedure. The vertical and horizontal dotted lines show the cutoff of fold change = ±1.5 and adjusted *P* = 0.01, respectively. **h**, IFNβ secretion measured by enzyme-linked immunosorbent assay (ELISA) in ex vivo cultured *Cx3cr1*-Cre^ERT2^; *Daxx*^fl/fl^ microglia and *Cx3cr1*-Cre^ERT2^; *Daxx*^WT/WT^ microglia under basal conditions or following 24 h of LPS treatment (*n* = 3 independent preparations per group; data were analyzed by ordinary two-way ANOVA, *F* = 251.4, *P* < 0.0001; Sidak’s multiple comparisons test, *P*_PBS_ > 0.9999; *P*_LPS_ < 0.0001). **i**, GO analysis for upregulated genes in *Cx3cr1*-Cre^ERT2^; *Daxx*^fl/fl^ microglia compared to control microglia. Enrichment was assessed using a one-sided hypergeometric test with a Benjamini–Hochberg FDR correction for multiple comparisons; Neg., negative; Pos., positive; prod., production; replic., replication; Reg., regulation; Resp., response. **j**, Heat map showing *k*-means clustering (*k* = 5) of LPS-responsive LTR elements according to their expression in *Cx3cr1*-Cre^ERT2^; *Daxx*^fl/fl^ and *Cx3cr1*-Cre^ERT2^; *Daxx*^WT/WT^ primary microglia under basal conditions or following 6, 24 and 48 h of LPS stimulation. Clusters are named C1, C2, C3, C4 and C5 and are indicated on the left. **k**, GO analysis for genes neighboring LTR elements in C2 and C4 identified in **j**. Statistical significance was determined using Genomic Regions Enrichment of Annotations Tool (GREAT), which applies a two-sided binomial test over genomic regions. **l**, Heat map showing gene expression neighboring the top 30 overexpressed intergenic LTRs in DAXX-deficient microglia at steady state and in LPS-stimulated control microglia; Nan, not applicable. **m**, Heat map showing expression of RTE repressor genes in *Cx3cr1*-Cre^ERT2^; *Daxx*^fl/fl^ and *Cx3cr1*-Cre^ERT2^; *Daxx*^WT/WT^ primary microglia under basal conditions or following 6, 24 and 48 h of LPS stimulation. Data are presented as mean ± s.e.m. Asterisks (*) indicate statistical significance: *P < 0.05 and ***P < 0.001; NS, not significant. Source data

**Extended Data Fig. 3:**
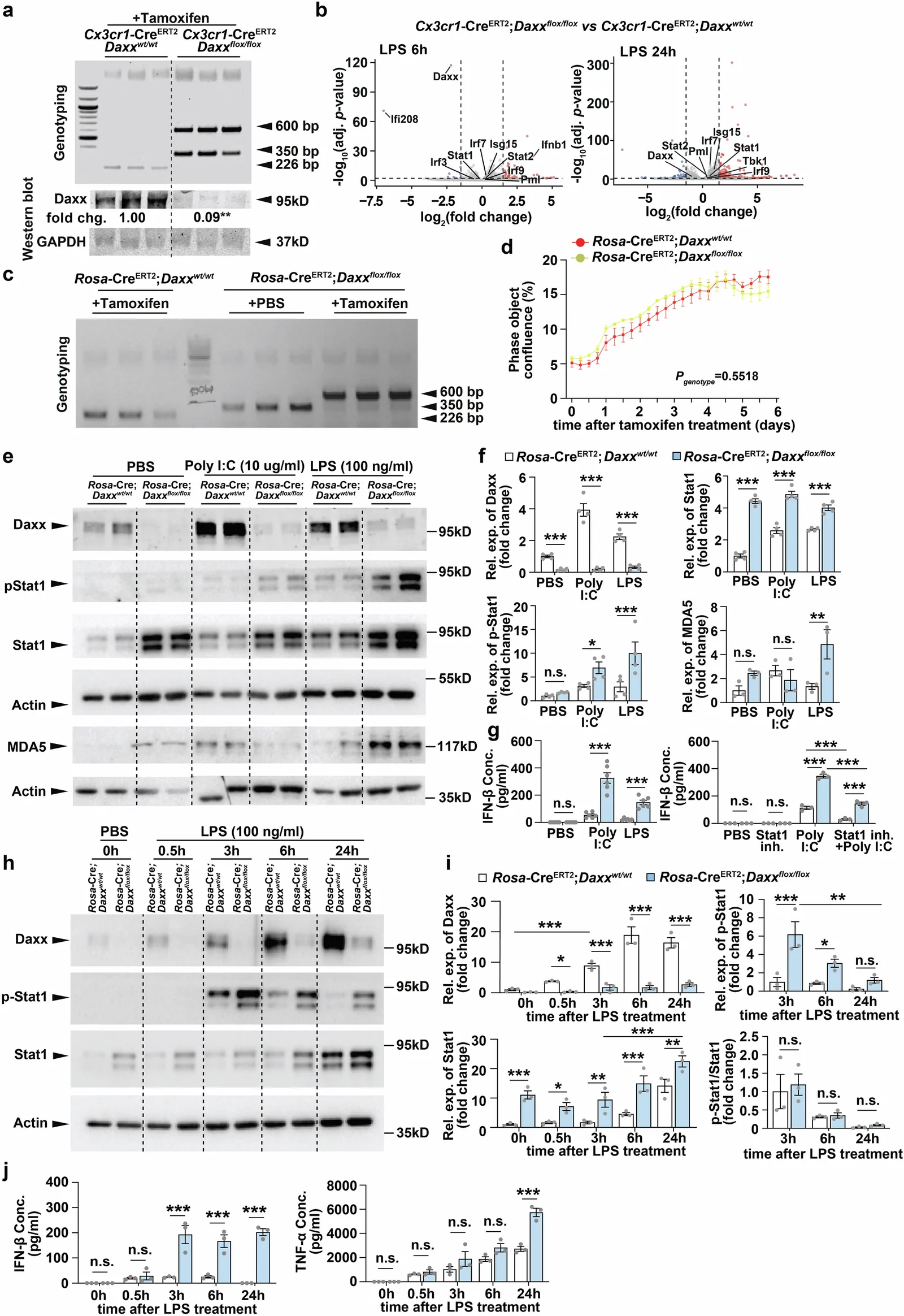
Genetic deletion of *Daxx* results in Stat1 induction and cytokine secretion in primary microglia and BMDMs. **a**, Genotyping and immunoblot in primary microglia from *Cx3cr1*-Cre^ERT2^;*Daxx*^*wt/wt*^ and *Cx3cr1*-Cre^ERT2^;*Daxx*^*flox/flox*^ (*n* = 3 mice per genotype). Arrows indicate the wild-type (226 bp), floxed (350 bp) and recombined (600 bp) alleles. Dayy protein levels were quantified by western blots (Comparison by two-tailed unpaired Student’s *t* test, *P* = 0.0056). **b**, Volcano plot showing differentially expressed genes in Daxx-deficient versus control primary microglia following 6 h or 24 h LPS stimulation. Differential expression analysis was assessed using two-sided Wald tests implemented in DESeq2, *P* values were adjusted for multiple testing using Benjamini–Hochberg correction. **c**, Genotyping PCR confirming *ex vivo* Daxx recombination in *Rosa*-Cre^ERT2^;*Daxx*^*flox/flox*^ BMDMs following 24 h 4’-OH-tamoxifen treatment. **d**, Real-time proliferation of *Rosa*-Cre^ERT2^;*Daxx*^*flox/flox*^ and *Rosa*-Cre^ERT2^;*Daxx*^*wt/wt*^ BMDMs monitored for 6 days using IncuCyte S3 system (*n* = 3 independent preparations per group, comparison by two-way RM ANOVA with Sidak’s multiple comparisons test, *F*_*genotype*_ (1,4) = 0.4210, *P*_*genotype*_ = 0.5518). **e**, **f**, Western blot and quantification of Daxx, Stat1, p-Stat1 and MDA5 in *Rosa*-Cre^ERT2^;*Daxx*^*flox/flox*^ and *Rosa*-Cre^ERT2^;*Daxx*^*wt/wt*^ BMDMs after 6 h Poly I:C or LPS stimulation (*n* = 4 independent preparations per group, comparison by ordinary two-way ANOVA with Sidak’s multiple comparisons test: for Daxx, *F* = 31.81, *P*_PBS Daxx WT vs Daxx KO_ = 0.0052, *P*_PolyI:C Daxx WT vs Daxx KO_ < 0.0001, *P*_LPS Daxx WT vs Daxx KO_ < 0.0001; for Stat1, *F* = 23.16, *P*_PBS Daxx WT vs Daxx KO_ < 0.0001, *P*_PolyI:C Daxx WT vs Daxx KO_ < 0.0001, *P*_LPS Daxx WT vs Daxx KO_ < 0.0001; for p-Stat1, *F* = 3.52, *P*_PBS Daxx WT vs Daxx KO_ = 0.6584, *P*_PolyI:C Daxx WT vs Daxx KO_ = 0.0354, *P*_LPS Daxx WT vs Daxx KO_ = 0.0005,; for MDA5, *F* = 5.178, *P*_PBS Daxx WT vs Daxx KO_ = 0.1522, *P*_PolyI:C Daxx WT vs Daxx KO_ = 0.4146, *P*_LPS Daxx WT vs Daxx KO_ = 0.003). **g**, IFN-β secretion measured by ELISA in *Daxx* WT and *Daxx* KO BMDMs after 6h-Poly I:C or LPS stimulation (left panel: *n* = 6 independent preparations per group, comparison by ordinary two-way ANOVA with Sidak’s multiple comparisons test: *F* = 30.04, *P*_PBS Daxx WT vs Daxx KO_ > 0.9999, *P*_PolyI:C Daxx WT vs Daxx KO_ < 0.0001);. or following Stat1 inhibitor pretreatment for 24 h before Poly I:C stimulation (right panel, *n* = 3 independent preparations per group, comparison by ordinary two-way ANOVA with Sidak’s multiple comparisons test: *F* = 93.72, *P*_PBS Daxx WT vs Daxx KO_ > 0.9999, *P*_Stat1 inh. Daxx WT vs Daxx KO_ > 0.9999, *P*_PolyI:C Daxx WT vs Daxx KO_ < 0.0001, *P*_PolyI:C+Stat1 inh. Daxx WT vs PDaxx KO_ < 0.0001). **h**, **i**, Western blot and quantification analysis of Daxx, Stat1, and p-Stat1 in *Rosa*-Cre^ERT2^;*Daxx*^*flox/flox*^ and *Rosa*-Cre^ERT2^;*Daxx*^*wt/wt*^ BMDMs following LPS stimulation for the indicated times (*n* = 3 independent preparations per group, comparison by two-way RM ANOVA with Tukey’s multiple comparisons test: for Daxx, *F* = 24.98, *P*_0h Daxx WT vs Daxx KO_ = 0.6021, *P*_0.5h Daxx WT vs Daxx KO_ = 0.0433, *P*_3h Daxx WT vs Daxx KO_ = 0.0002, *P*_6h Daxx WT vs Daxx KO_ < 0.0001, *P*_24h Daxx WT vs Daxx KO_ < 0.0001, *P*_3h Daxx WT vs 0h Daxx WT_ = 0.0003; for Stat1, *F* = 1.231, *P*_0h Daxx WT vs Daxx KO_ = 0.0003, *P*_0.5h Daxx WT vs Daxx KO_ = 0.027, *P*_3h Daxx WT vs Daxx KO_ = 0.003, *P*_6h Daxx WT vs Daxx KO_ = 0.0002, *P*_24h Daxx WT vs Daxx KO_ = 0.0017; for p-Stat1, *F* = 5.179, *P*_3h Daxx WT vs Daxx KO_ = 0.0001, *P*_6h Daxx WT vs Daxx KO_ = 0.034, *P*_24h Daxx WT vs 2Daxx KO_ = 0.3201; for p-Stat1/Stat1, *F* = 0.05768, *P*_3h Daxx WT vs Daxx KO_ = 0.5658, *P*_6h Daxx WT vs Daxx KO_ = 0.9001, *P*_24h Daxx WT vs Daxx KO_ = 0.8529). **j**, IFN-β and TNF-α concentrations in the BMDM supernatants measured by ELISA follwing LPS stimulation for the indicated times (*n* = 3 independent preparations per group, comparison by ordinary two-way ANOVA with Sidak’s multiple comparisons test: for IFN-β, *F* = 18.17, *P*_0h Daxx WT vs Daxx KO_ > 0.9999, *P*_0.5h Daxx WT vs Daxx KO_ = 0.9975, *P*_3h Daxx WT vs Daxx KO_ < 0.0001, *P*_6h Daxx WT vs Daxx KO_ < 0.0001, *P*_24h Daxx WT vs Daxx KO_ < 0.0001; for TNF-α, *F* = 9.169, *P*_0h Daxx WT vs Daxx KO_ > 0.9999, *P*_0.5h Daxx WT vs Daxx KO_ = 0.9917, *P*_3h Daxx WT vs Daxx KO_ = 0.1903, *P*_6h Daxx WT vs Daxx KO_ = 0.129, *P*_24h Daxx WT vs Daxx KO_ < 0.0001). Data are presented as mean ± s.e.m. Asterisks (*) indicate statistical significance: *P < 0.05, **P < 0.01 and ***P < 0.001; n.s. indicates not significant. Source data

To assess the impact of *Daxx* loss, we generated a tamoxifen (TAM)-inducible conditional knockout mouse line (*Cx3cr1*-Cre^ERT2^; *Daxx*^fl/fl^) for *Daxx* ablation in tissue-resident macrophages and microglia^39^. Incubation with 4-hydroxytamoxifen for 24 h led to *Daxx* recombination and reduced protein levels in *Cx3cr1*-Cre^ERT2^; *Daxx*^fl/fl^ primary microglia (Fig. 2a,b and Extended Data Fig. 3a), associated with induction CD68, a phagolysosome marker indicative of a microglia reactive state (Fig. 2c,d). RNA-seq analysis showed pronounced changes in gene expression in unstimulated cells (Fig. 2e and Extended Data Fig. 3b), with most deregulated genes representing loci upregulated by LPS in control microglia (Fig. 2f,g). IFNβ levels were higher in DAXX-deficient microglia following LPS treatment, but not at steady state (Fig. 2h), suggesting that gene expression alterations are IFNβ independent or rely on undetectable IFNβ levels in the absence of a stimulus. Gene Ontology (GO) analysis revealed that genes upregulated in DAXX*-*deficient microglia at steady state were enriched in antiviral response and immune IFNβ processing terms (Fig. 2i).

*Daxx* loss in *Rosa*-Cre^ERT2^; *Daxx*^fl/fl^ BMDMs also led to increased expression of ISGs, such as *Stat1* and *Mda5*, the latter a gene encoding an RNA-sensing protein, without discernable effects on viability (Extended Data Fig. 3c–f). As in microglia, IFNβ secretion was more pronounced in *Rosa*-Cre^ERT2^; *Daxx*^fl/fl^ BMDMs following poly(I:C) or LPS treatment (Extended Data Fig. 3g). Pretreatment with a STAT1 pharmacological inhibitor reduced IFNβ secretion in both genotypes (Extended Data Fig. 3g). While STAT1 protein expression was higher in *Rosa*-Cre^ERT2^; *Daxx*^fl/fl^ BMDMs at both steady state and following stimulation, there was no net induction of STAT1 phosphorylation (Extended Data Fig. 3h,i). IFNβ secretion remained higher in DAXX-deficient cells than in control cells up to 24 h (Extended Data Fig. 3j). Finally, TNF was augmented only at 24 h, suggesting an effect of DAXX loss on NF-κB-mediated transcription (Extended Data Fig. 3j).

Next, we assessed the impact of DAXX depletion on RTE expression. While LTR-RTEs peaked at 6 h and then returned to steady state levels in 4-hydroxytamoxifen-treated *Cx3cr1*-Cre^ERT2^; *Daxx*^WT/WT^ microglia (Fig. 2j), in *Cx3cr1*-Cre^ERT2^; *Daxx*^fl/fl^ microglia, a subset of RTEs displayed prolonged expression (C2 and C4 clusters; Fig. 2j). By combining locus-specific analysis and GO analysis, we revealed that RTEs in C2 and C4 displayed coordinated induction with ISGs in response to inflammatory stimuli (Fig. 2k). Interestingly, a number of the RTE–ISG pairs induced by LPS in *Cx3cr1*-Cre^ERT2^; *Daxx*^WT/WT^ cells were derepressed at steady state following *Daxx* loss (Fig. 2k,l). Finally, DAXX depletion correlated with the induction of other RTE repressors, including *Chaf1b*, *Setdb1*, *Dnmt1*, *Dnmt3a*, *H3f3a* and *H3f3b* (Fig. 2m), suggesting potential compensation for RTE silencing in *Cx3cr1*-Cre^ERT2^; *Daxx*^fl/fl^ microglia.

Together, these findings suggest that DAXX ensures RTE and ISG repression in unstimulated cells, while its IFNβ-dependent induction contributes to resolution of the inflammatory response.

### Loss of *Daxx* in vivo causes altered microglial morphology, microglia depletion and astrogliosis

**Fig. 3:**
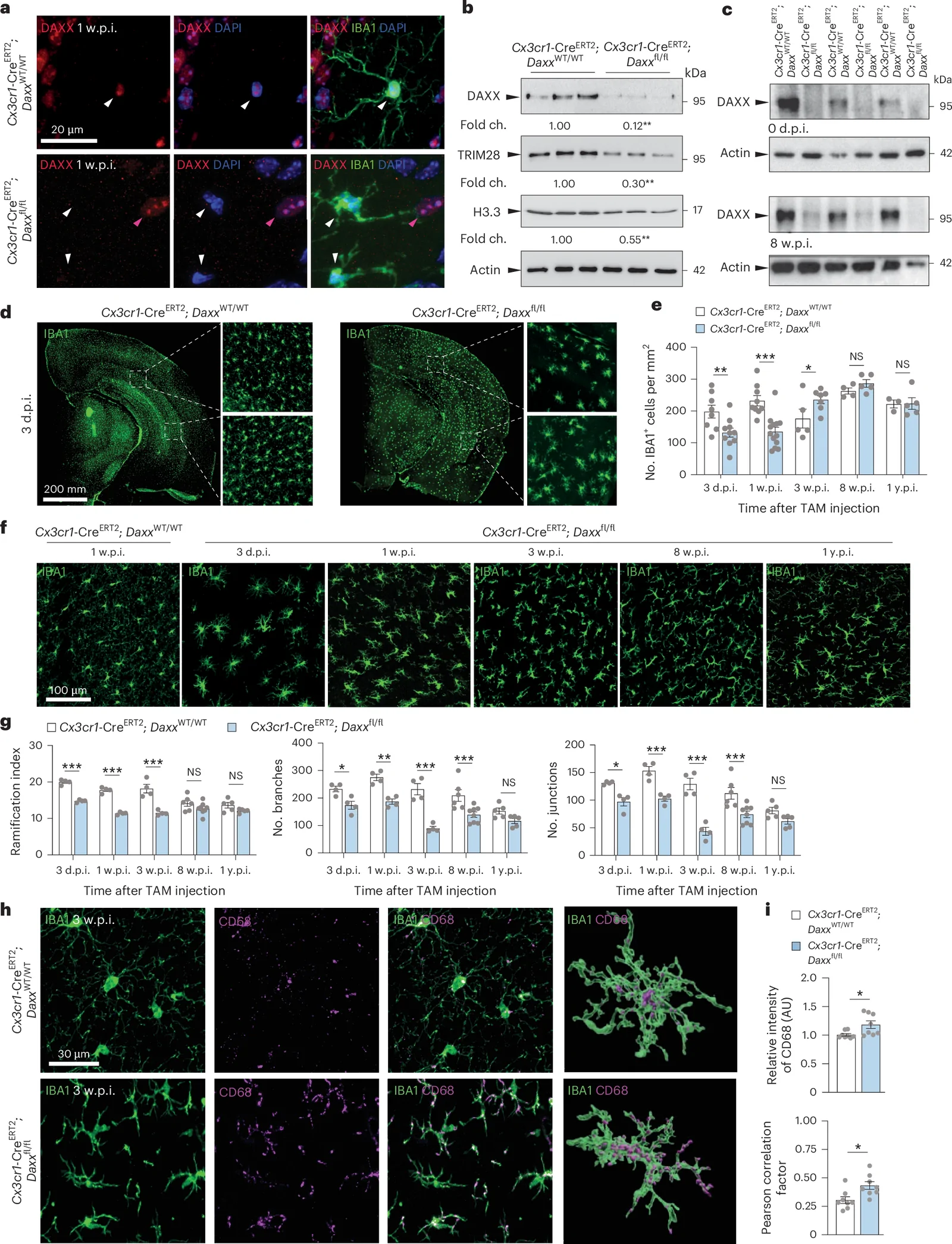
Deletion of *Daxx* in microglia leads to a reactive phenotype in vivo. **a**, Representative confocal images of DAXX and IBA1 double immunostaining in brain sections from *Cx3cr1*-Cre^ERT2^; *Daxx*^WT/WT^ and *Cx3cr1*-Cre^ERT2^; *Daxx*^fl/fl^ mice (*n* = 3 mice for each genotype), at 1 w.p.i. **b**, Western blot and quantification of DAXX, TRIM28 and H3.3 in microglia isolated from *Cx3cr1*-Cre^ERT2^; *Daxx*^WT/WT^ and *Cx3cr1*-Cre^ERT2^; *Daxx*^fl/fl^ mice at 1 w.p.i. β-Actin served as a loading control (*n* = 3 mice for each genotype; data were analyzed by two-tailed unpaired Student’s *t*-test, *P*_DAXX_ = 0.0094, *P*_TRIM28_ = 0.0011, *P*_H3.3_ = 0.0029). **c**, Western blots for DAXX expression in microglia isolated from *Cx3cr1*-Cre^ERT2^; *Daxx*^WT/WT^ and *Cx3cr1*-Cre^ERT2^; *Daxx*^fl/fl^ mice at 0 d.p.i. and 8 w.p.i. (*n* = 3 mice for each genotype). β-Actin served as a loading control. **d**, Representative confocal images of IBA1 immunostaining in brain sections from *Cx3cr1*-Cre^ERT2^; *Daxx*^WT/WT^ and *Cx3cr1*-Cre^ERT2^; *Daxx*^fl/fl^ mice at 3 d.p.i. **e**, Quantification of IBA1^+^ cells in the hippocampus of *Cx3cr1*-Cre^ERT2^; *Daxx*^WT/WT^ and *Cx3cr1*-Cre^ERT2^; *Daxx*^fl/fl^ mice at 3 d.p.i. (*n* = 8 mice for *Cx3cr1*-Cre^ERT2^; *Daxx*^WT/WT^ and *n* = 10 mice for *Cx3cr1*-Cre^ERT2^; *Daxx*^fl/fl^), 1 w.p.i. (*n* = 9 mice for *Cx3cr1*-Cre^ERT2^; *Daxx*^WT/WT^ and *n* = 13 mice for *Cx3cr1*-Cre^ERT2^; *Daxx*^fl/fl^), 3 w.p.i. (*n* = 5 mice for *Cx3cr1*-Cre^ERT2^; *Daxx*^WT/WT^ and *n* = 7 mice for *Cx3cr1*-Cre^ERT2^; *Daxx*^fl/fl^), 8 w.p.i. (*n* = 4 mice for *Cx3cr1*-Cre^ERT2^; *Daxx*^WT/WT^ and *n* = 5 mice for *Cx3cr1*-Cre^ERT2^; *Daxx*^fl/fl^) and 1 y.p.i. (*n* = 3 mice for *Cx3cr1*-Cre^ERT2^; *Daxx*^WT/WT^ and *n* = 5 mice for *Cx3cr1*-Cre^ERT2^; *Daxx*^fl/fl^; data were analyzed by ordinary two-way ANOVA, *F* = 7.177, *P* < 0.0001; Sidak’s multiple comparisons test *P* values for *Cx3cr1*-Cre^ERT2^; *Daxx*^fl/fl^ versus *Cx3cr1*-Cre^ERT2^; *Daxx*^WT/WT^: *P*_3 d.p.i._ = 0.0037, *P*_1 w.p.i._ < 0.0001, *P*_3 w.p.i._ = 0.0318, *P*_8 w.p.i._ = 0.4425, *P*_1 y.p.i._ = 0.9669). **f**,**g**, Representative confocal images of IBA1 immunostaining (**f**) and morphological quantification (**g**) of IBA1^+^ cells in the CA1 region of the hippocampus from *Cx3cr1*-Cre^ERT2^; *Daxx*^WT/WT^ and *Cx3cr1*-Cre^ERT2^; *Daxx*^fl/fl^ mice at 3 d.p.i. (*n* = 4 mice per group), 1 w.p.i. (*n* = 4 mice per group), 3 w.p.i. (*n* = 4 mice per group), 8 w.p.i. (*n* = 6 mice for *Cx3cr1*-Cre^ERT2^; *Daxx*^WT/WT^ and *n* = 8 mice for *Cx3cr1*-Cre^ERT2^; *Daxx*^fl/fl^) and 1 y.p.i. (*n* = 5 mice per group; data were analyzed by ordinary two-way ANOVA with a Sidak’s multiple comparisons test; *P* values for *Cx3cr1*-Cre^ERT2^; *Daxx*^fl/fl^ versus *Cx3cr1*-Cre^ERT2^; *Daxx*^WT/WT^: ramification index: *P*_3 d.p.i._ < 0.0001, *P*_1 w.p.i._ < 0.0001, *P*_3 w.p.i._ < 0.0001, *P*_8 w.p.i._ = 0.2834, *P*_1 y.p.i._ = 0.3752; number of branches: *P*_3 d.p.i._ = 0.0457, *P*_1 w.p.i._ = 0.0011, *P*_3 w.p.i._ < 0.0001, *P*_8 w.p.i._ = 0.0007, *P*_1 y.p.i._ = 0.2958; number of junctions: *P*_3 d.p.i._ = 0.0267, *P*_1 w.p.i._ = 0.0005, *P*_3 w.p.i._ < 0.0001, *P*_8 w.p.i._ = 0.0005, *P*_1 y.p.i._ = 0.2962). **h**,**i**, Representative confocal images (**h**) and quantification (**i**) of IBA1 and CD68 immunostaining in the hippocampus from *Cx3cr1*-Cre^ERT2^; *Daxx*^WT/WT^ and *Cx3cr1*-Cre^ERT2^; *Daxx*^fl/fl^ mice at 3 w.p.i. (*n* = 8 mice for each genotype; data were analyzed by two-tailed unpaired Student’s *t*-test, *P*_CD68 intensity_ = 0.024, *P*_Pearson correlation_ = 0.0125). The images on the far right in **h** show three-dimensional reconstructions of IBA1^+^ microglia with IBA1^+^CD68^+^ double immunostaining. Data are presented as mean ± s.e.m. Asterisks (*) indicate statistical significance: *P < 0.05, **P < 0.01 and ***P < 0.001; AU, arbitrary units. Source data

To investigate the impact of *Daxx* loss in microglia in vivo, *Cx3cr1*-Cre^ERT2^; *Daxx*^fl/fl^ and *Cx3cr1*-Cre^ERT2^; *Daxx*^WT/WT^ mice were treated with TAM and assessed at 0 days post-TAM treatment (d.p.i.), 1 d.p.i., 3 d.p.i., 1 week post-TAM treatment (1 w.p.i.), 3 w.p.i., 8 w.p.i. and 1 year post-TAM treatment (1 y.p.i.). Cre-mediated *Daxx* recombination was observed in column-isolated *Cx3cr1*-Cre^ERT2^; *Daxx*^fl/fl^ microglia at 3 w.p.i., but not in the bone marrow, in accordance with the rapid turnover of peripheral macrophages (Supplementary Fig. 1a,b). DAXX downregulation was demonstrated across the entire time course from TAM treatment, correlating with reduced expression of two DAXX-interacting proteins, H3.3 (Fig. 3a–c), as we have previously shown^27^, and TRIM28 (Fig. 3a–c).

Following *Daxx* loss, the number of IBA1^+^ microglia was reduced at 3 d.p.i. and 1 w.p.i. but then recovered from 3 w.p.i. onward (Fig. 3d,e). Concomitantly, microglia morphology acquired reactive features along with increased CD68 (Fig. 3f–i and Supplementary Fig. 1e), more markedly in the ventral CA1 (Supplementary Fig. 1d,e). Microglia morphology and numbers were unaffected at 0 d.p.i. in both genotypes (Supplementary Fig. 1c), excluding an effect of Cre or TAM treatment. Correlating with decreased microglia cellularity, the percentage of cleaved caspase-3^+^IBA1^+^ cells was increased following *Daxx* loss at 3 d.p.i. but not later (Supplementary Fig. 1f–i). Finally, the number of Ki67^+^IBA1^+^ proliferating microglia was also increased at the early time points (Supplementary Fig. 2a–c).

In accordance with the reported association between microglia activation and astrogliosis^40^, glial fibrillary acidic protein (GFAP) immunoreactivity was increased in *Cx3cr1*-Cre^ERT2^; *Daxx*^fl/fl^ brains from 1 w.p.i. onward, corroborated by western blots on whole-brain lysates (Supplementary Fig. 2d–f). Finally, the number of NeuN^+^ neurons, the area coverage of myelin basic protein (MBP) and the neurofilament marker SMI31 were not altered in the CA1 of *Cx3cr1*-Cre^ERT2^; *Daxx*^fl/fl^ hippocampi at 3 w.p.i. and 8 w.p.i. (Supplementary Fig. 2g–j).

Collectively, *Daxx* loss leads to induction of a microglia reactive state and astrogliosis, which are associated with cell death and entry into the cell cycle.

### Mice lacking microglial *Daxx* display reduced synaptic markers/puncta and behavioral alterations

**Fig. 4:**
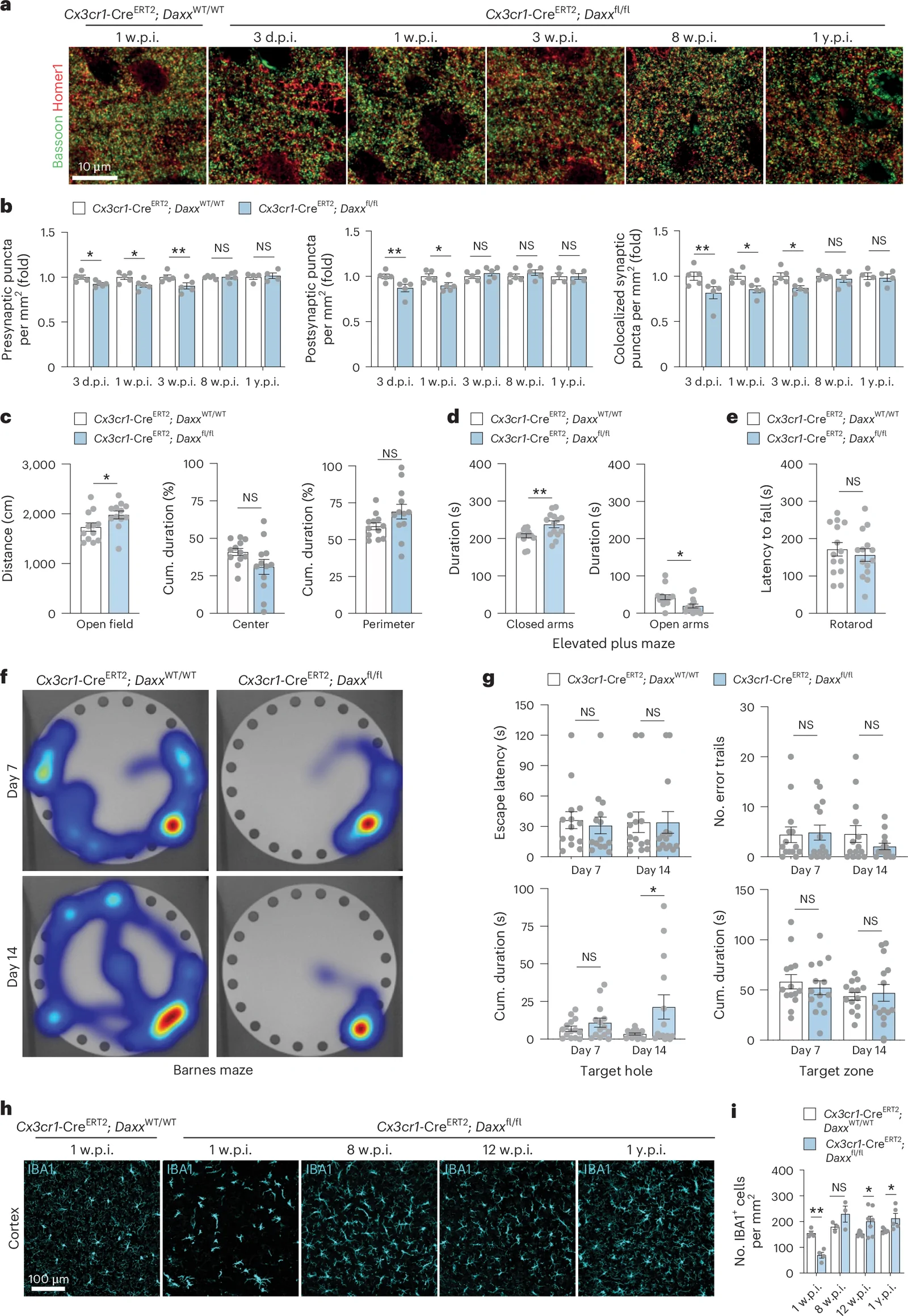
Mice lacking microglial *Daxx* display synapse puncta reduction and alterations of behavior. Behavioral characterization of *Cx3cr1*-Cre^ERT2^; *Daxx*^WT/WT^ and *Cx3cr1*-Cre^ERT2^; *Daxx*^fl/fl^ mice at 8 w.p.i. Each data point represents one animal. **a**,**b**, Representative confocal images (**a**) and quantification (**b**) of Bassoon and Homer1 immunostaining in the prefrontal cortex of *Cx3cr1*-Cre^ERT2^; *Daxx*^fl/fl^ and *Cx3cr1*-Cre^ERT2^; *Daxx*^WT/WT^ mice at 3 d.p.i. (*n* = 5 mice for each genotype), 1 w.p.i. (*n* = 5 mice for each genotype), 3 w.p.i. (*n* = 5 mice for each genotype), 8 w.p.i. (*n* = 5 mice for each genotype) and 1 y.p.i. (*n* = 4 mice for each genotype; data were analyzed by ordinary two-way ANOVA with a Sidak’s multiple comparisons test; *P* values for *Cx3cr1*-Cre^ERT2^; *Daxx*^fl/fl^ mice versus *Cx3cr1*-Cre^ERT2^; *Daxx*^WT/WT^, presynaptic puncta: *P*_3 d.p.i._ = 0.0301, *P*_1 w.p.i._ = 0.0015, *P*_3 w.p.i._ = 0.006, *P*_8 w.p.i._ = 0.9238, *P*_1 y.p.i._ = 0.6803; postsynaptic puncta: *P*_3 d.p.i._ = 0.0049, *P*_1 w.p.i._ = 0.0226, *P*_3 w.p.i._ = 0.408, *P*_8 w.p.i._ = 0.3306, *P*_1 y.p.i._ = 0.9975; colocalized puncta: *P*_3 d.p.i._ = 0.0023, *P*_1 w.p.i._ = 0.0134, *P*_3 w.p.i._ = 0.0254, *P*_8 w.p.i._ = 0.5976, *P*_1 y.p.i._ = 0.7808). **c**, Open-field test with travel distance and quantification of time spent in the center zone and in the perimeter zone (*n* = 12 mice for each genotype; data were analyzed by two-tailed unpaired Student’s *t*-test, *P*_Distance_ = 0.0452, *P*_Duration center_ = 0.1595, *P*_Duration perimeter_ = 0.1595); Cum., cumulative. **d**, Elevated plus maze test showing time spent in the closed and open arms (*n* = 14 mice for each genotype; data were analyzed by two-tailed unpaired Student’s *t*-test, *P*_Closed arms_ = 0.0073, *P*_Open arms_ = 0.0108). **e**, Rotarod test assessed by latency to fall from the accelerating rotarod (*n* = 14 mice for each genotype; data were analyzed by two-tailed unpaired Student’s *t*-test, *P* = 0.5425). **f**, Representative tracking plots from the Barnes maze probe trails on days 7 and 14. **g**, Barnes maze test showing escape latency, number of error trails and time spent at the target hole and target zone (*n* = 14 mice for each genotype; data were analyzed by two-way repeated-measures ANOVA with a Sidak’s multiple comparisons test; *P* values for *Cx3cr1*-Cre^ERT2^; *Daxx*^fl/fl^ mice versus *Cx3cr1*-Cre^ERT2^; *Daxx*^WT/WT^: escape latency, *P*_day 7_ = 0.9069, *P*_day 14_ = 0.9999; number of error trails, *P*_day 7_ = 0.9374, *P*_day 14_ = 0.3888; target hole, *P*_day 7_ = 0.7754, *P*_day 14_ = 0.0115; target zone, *P*_day 7_ = 0.7816, *P*_day 14_ = 0.9218). **h**,**i**, Representative confocal images (**h**) and quantification (**i**) of IBA1^+^ cells in the prefrontal cortex of *Cx3cr1*-Cre^ERT2^; *Daxx*^fl/fl^ and *Cx3cr1*-Cre^ERT2^; *Daxx*^WT/WT^ mice at 1 w.p.i. (*n* = 4 mice for each genotype), 8 w.p.i. (*n* = 3 mice for each genotype), 12 w.p.i. (*n* = 7 mice for each genotype) and 1 y.p.i. (*n* = 5 mice for each genotype; data were analyzed by ordinary two-way ANOVA, *F* = 8.386, *P* = 0.0003; Sidak’s multiple comparisons test *P* values for *Cx3cr1*-Cre^ERT2^; *Daxx*^fl/fl^ versus *Cx3cr1*-Cre^ERT2^; *Daxx*^WT/WT^ mice: *P*_1 w.p.i._ = 0.0011, *P*_8 w.p.i._ = 0.0809, *P*_12 w.p.i._ = 0.0117, *P*_1 y.p.i._ = 0.0333). Data are presented as mean ± s.e.m. Asterisks (*) indicate statistical significance: *P < 0.05 and **P < 0.01. Source data

Because reactive microglia may mediate synapse pruning and remodeling^41^, we assessed Bassoon^+^Homer1^+^ synaptic puncta and found a transient reduction following microglial *Daxx* loss (Fig. 4a,b), correlating with decreased synaptic protein expression in whole-brain lysates (Supplementary Fig. 2f). We then assessed whether these changes correlated with behavioral perturbations, given that dysfunctional microglia have been linked to altered behavior in mice, including anxiety-like phenotypes^42,43^. We then performed a panel of tests to characterize different domains of mouse behavior at 8 w.p.i., when microglia numbers had recovered to control levels. *Cx3cr1*-Cre^ERT2^; *Daxx*^fl/fl^ mice traveled longer distances in the open-field arena and spent slightly reduced time exploring the center of the arena (Fig. 4c), suggesting increased anxiety. This is in agreement with the more pronounced changes in microglial morphology in the prefrontal cortex (Fig. 4h,i), a region implicated in fear and anxiety^42,44^. Results from the elevated plus maze test also indicated anxiety-like behavior (Fig. 4d), with lack of discernible defects in locomotion (Fig. 4e). Finally, Barnes maze tests for spatial learning and memory function suggested that the motivation of *Cx3cr1*-Cre^ERT2^; *Daxx*^fl/fl^ mice to search for another escape of the maze declined, indicating a potential stress-response search strategy or executive dysfunction. While the escape latency as well as the number of serial errors were unchanged (Fig. 4g), *Cx3cr1*-Cre^ERT2^; *Daxx*^fl/fl^ mice spent more time in the target hole at probe day 14 (Fig. 4f,g), suggesting that they remember the target location more effectively, whereas their search strategy may result from anxiety-driven avoidance of alternative holes.

Overall, our findings suggest that altered microglia function following *Daxx* loss has implications for brain function and behavior.

### *Daxx* loss causes early RTE upregulation and a biphasic gene expression response

**Fig. 5:**
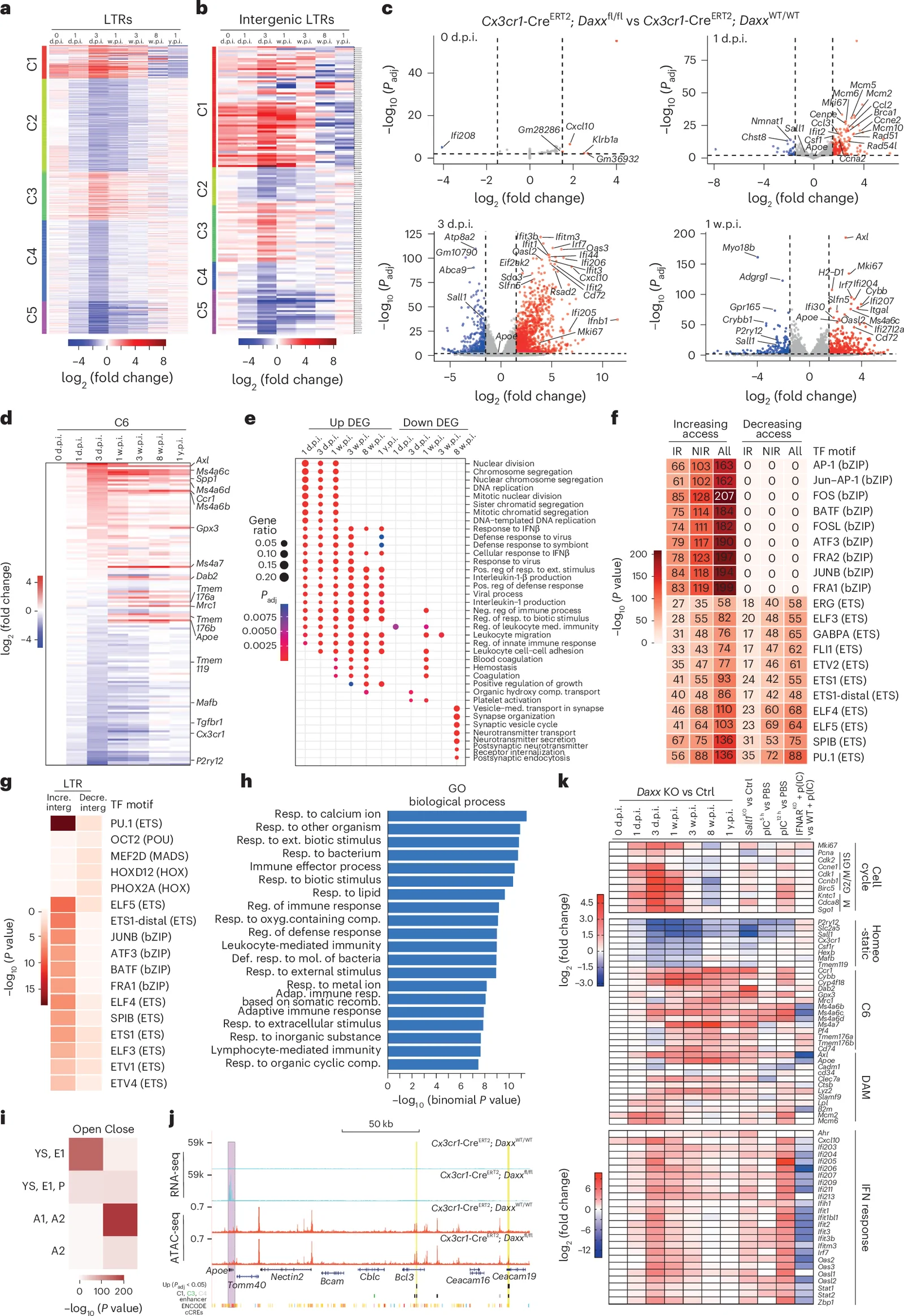
Loss of *Daxx* in microglia in vivo causes derepression of RTEs and marked changes of the microglia transcriptome. **a**,**b**, Heat maps showing *k* component Gaussian mixture model clustering (*k* = 5) of differentially expressed LTRs (**a**) and intergenic LTRs (**b**) in microglia isolated from *Cx3cr1*-Cre^ERT2^; *Daxx*^fl/fl^ mice compared to *Cx3cr1*-Cre^ERT2^; *Daxx*^WT/WT^ mice from 0 d.p.i. to 1 y.p.i. Clusters are indicated on the left. **c**, Volcano plots showing the differentially expressed genes in in vivo microglia isolated from *Cx3cr1*-Cre^ERT2^; *Daxx*^fl/fl^ mice compared to *Cx3cr1*-Cre^ERT2^; *Daxx*^WT/WT^ mice from 0 d.p.i. to 1 w.p.i. Differential expression analysis was performed using DESeq2 with a two-sided Wald test, and *P* values were adjusted for multiple testing using the Benjamini–Hochberg procedure. The vertical and horizontal dotted lines show the cutoff of fold change = ±1.5 and an adjusted *P* value of 0.01, respectively. **d**, Heat map showing changes in gene expression associated with the developing microglial phenotype (named C6, according to published datasets in Hammond et al.^45^) in *Cx3cr1*-Cre^ERT2^; *Daxx*^fl/fl^ microglia compared to in *Cx3cr1*-Cre^ERT2^; *Daxx*^WT/WT^ microglia from 0 d.p.i. to 1 y.p.i. **e**, GO analysis of differentially expressed genes (DEGs) in *Cx3cr1*-Cre^ERT2^; *Daxx*^fl/fl^ microglia compared to *Cx3cr1*-Cre^ERT2^; *Daxx*^WT/WT^ microglia from 1 d.p.i. to 1 y.p.i. Differential expression analysis was performed using a two-sided hypergeometric test via the clusterProfiler::compareCluster package, and *P* values were adjusted for genome-wide multiple comparisons using the Benjamini–Hochberg FDR method; comp., compound; ext., external; med., mediated. **f**, Heat map showing TF motif enrichment in open and closed differentially accessible regions (DARs) identified by ATAC-seq in *Cx3cr1*-Cre^ERT2^; *Daxx*^fl/fl^ microglia at 3 d.p.i. Regions are classified as intergenic (IR) or nonintergenic (NIR). Differential expression analysis was performed using a one-sided hypergeometric test against an automatically generated background set of genomic sequences via HOMER software. **g**, Heat map showing TF motif analysis in LTR-overlapping DARs located in intergenic (interg) regions of *Cx3cr1*-Cre^ERT2^; *Daxx*^fl/fl^ microglia at 3 d.p.i. The color grade represents –log_10_ (*P* value) for each motif. Differential expression analysis was performed using a one-sided hypergeometric test against an automatically generated background set of genomic sequences via HOMER software; Decre., decreased; Incre., increased. **h**, GO analysis of genes near opened chromatin regions at 3 d.p.i. overlapped with LTRs in intergenic regions. Differential expression analysis was performed using a two-sided binomial test over genomic regions; Adap., adaptive; Def., defensive; mol., molecule; oxyg.containing, oxygen-containing; recomb., recombination. **i**, Heat map showing overlap between differential ATAC-seq peaks in *Cx3cr1*-Cre^ERT2^; *Daxx*^fl/fl^ microglia at 3 d.p.i. and enhancer regions associated with developmental microglial states (data from ref. ^30^). Differential expression analysis for each pairwise genomic intersection was performed using a two-sided Fisher’s exact test via the ‘bedtools fisher’ command. **j**, Genome browser tracks showing ATAC-seq and RNA-seq signal at the *Apoe* locus in *Cx3cr1*-Cre^ERT2^; *Daxx*^WT/WT^ and *Cx3cr1*-Cre^ERT2^; *Daxx*^fl/fl^ microglia at 3 d.p.i. Highlighted regions indicate developmental microglial enhancers (yellow) and the *Apoe* gene locus (purple); cCREs, candidate *cis*-regulatory elements. **k**, Heat map showing relative expression of gene signatures associated with distinct microglia states, including homeostatic, cell cycle, developmental (C6)^45^, DAM^74^ and IFN response. Datasets include *Cx3cr1*-Cre^ERT2^; *Daxx*^fl/fl^ (*Daxx* KO) from 0 d.p.i. to 1 y.p.i., *Sall1*-Cre^ERT2^; *Sall1*^fl/fl^ (*Sall1* KO)^51^, poly(I:C)-stimulated wild-type microglia (p(IC)^5 h^, p(IC)^12 h^) and poly(I:C)-stimulated *Ifnar*-KO microglia (*Ifnar* KO + p(IC)). Abbreviations: A1, adult microglia cluster 1; A2, adult microglia cluster 2; E1, early microglia cluster 1; P, premicroglia; YS, yolk sac. Source data

Given the impact of microglial *Daxx* loss on tissue homeostasis and behavior, we decided to explore potential perturbations of the microglia transcriptome and chromatin landscape. Bulk RNA-seq data obtained from column-isolated microglia showed clear separation between control and DAXX-deficient cells by principal component analysis (PCA) across the different time points (Supplementary Fig. 3a). By performing *k*-means clustering, differentially expressed LTR-RTEs were separated into five clusters (C1–C5; Fig. 5a), with LTR-RTEs within C1 and C3 being induced starting from 0 d.p.i. and peaking at 3 d.p.i., while in C2, C4 and C5, LTR-RTEs were gradually inhibited until 3 d.p.i., followed by an increase at later stages. Of note, intergenic C1 and C3 LTR-RTEs were already upregulated at 0 d.p.i. in DAXX-deficient microglia (Fig. 5b). The gradual downregulation of RTEs at later stages after *Daxx* loss correlated with induction of the RTE repressors *Chaf1* and *Dnmt1* (Supplementary Fig. 3c), as also observed in vitro (Fig. 2m).

In contrast to RTE induction, alterations in endogenous gene expression were observed only from 1 d.p.i. and peaked at 3 d.p.i. and 1 w.p.i. (Fig. 5c and Supplementary Fig. 3b), suggesting that the primary effect of *Daxx* loss is on RTE expression. Data integration using published datasets^45^ showed an overlap between differentially expressed genes in DAXX-deficient microglia and signatures characterizing embryonic and early postnatal microglia (C3 and C6; Fig. 5d and Supplementary Fig. 3d). Genes found in C6 embryonic microglia also contained canonical DAM genes, like *Apoe* and *Axl*^45^ (Fig. 5d). Furthermore, differentially expressed genes in DAXX-deficient microglia overlapped with aging-dependent microglia signatures (Supplementary Fig. 3d)^33^. GO analysis showed that cell division/mitosis terms were enriched in DAXX-deficient microglia (Fig. 5e) but were restricted to 3 d.p.i. and 1 w.p.i. (Fig. 5e), in accordance with the peak in Ki67 positivity (Supplementary Fig. 2a–c). By contrast, immune response/inflammation terms remained stable throughout the entire time course (Fig. 5e).

We then addressed whether DAXX-deficient cells would be transcriptionally similar to reconstituting microglia following CSF1R inhibition^33,46^ (Supplementary Fig. 3d). Repopulation-associated signatures were up mostly before the observed replenishment detected at 3 w.p.i., suggesting a potential regenerative state of DAXX-deficient microglia. We also assessed whether *Daxx* loss recapitulated an exhausted microglia phenotype following three rounds of depletion/reconstitution (3×DR) described in a previous study^33^, which, unlike our current work, was run using female mice due to limited changes in the microglia transcriptome in male mice. Most of the differentially expressed genes from 3×DR female microglia displayed an opposite direction of change in DAXX-deficient microglia, while conversely a subset of 3×DR downregulated genes was upregulated in *Daxx*-KO microglia. Finally, differentially expressed genes that followed the same direction of change were mostly enriched at early time points (Supplementary Fig. 3d), before microglia pool replenishment. Finally, the genes from 3×DR that remained up until 8 w.p.i. and 1 y.p.i. mostly belonged to the DAM category. These findings suggest that DAXX-deficient microglia fail to clearly recapitulate exhausted microglia.

Because enzymatic digestion during microglia preparation induces stress and causes an aberrant transcriptional response^47^, we performed microglia isolation using an inhibitor cocktail to prevent transcription and translation^47^ and assessed the expression of a number of homogenization-induced genes and DAM genes (Supplementary Fig. 3e). The inhibitor cocktail downregulated the expression of the artifactual signature genes only in control microglia and not in DAXX-deficient microglia, whereas in untreated DAXX-deficient cells, these genes were as low as in inhibitor-treated wild-type cells. DAM genes were induced following *Daxx* loss, but they were unaffected by the inhibitor cocktail, overall indicating that transcriptome perturbations following *Daxx* depletion are predominantly caused by *Daxx* loss rather than cell isolation-induced artifacts.

Together, our data show that microglial *Daxx* loss leads to early RTE upregulation, followed by loss of homeostatic gene expression, a transient reactive/developmental state and stable induction of DAM genes.

### Increased chromatin accessibility at RTEs as well as developmental and aging-associated enhancers following *Daxx* loss

To assess the effect of *Daxx* depletion on the chromatin landscape, we performed assay for transposase-accessible chromatin using sequencing (ATAC-seq) at 3 d.p.i. to capture early changes in chromatin accessibility. PCA revealed clear clustering according to genotype (Supplementary Fig. 3f), whereas motif enrichment analysis showed that regions that gained accessibility were enriched in AP-1-related motifs (Fig. 5f), consistent with a microglia reactive state^48,49^ (Fig. 5f). By contrast, intergenic LTR-RTEs gaining accessibility were enriched in the PU.1 motif (Fig. 5g), as in DAXX-deficient hematopoietic progenitors^27^. GO analysis indicated that intergenic LTR-RTEs gaining accessibility were in close proximity to immune regulation genes and ISGs (Fig. 5h). By accessing published data^45^, we showed that a subset of developmental enhancers (YS/E1 cluster) showed increased accessibility in DAXX-deficient microglia (Fig. 5i) and were close to deregulated ISGs and DAM genes, including regulatory regions within the *Ceacam19* locus downstream of *Apoe* (Fig. 5j and Supplementary Fig. 3g). By contrast, enhancers losing accessibility were associated with adult microglia (A1/A2 clusters) and in proximity to downregulated homeostatic genes, such as *Sall1*, *P2ry12*, *Cx3cr1* and *Mafb* (Supplementary Fig. 3h), correlating with *Sall1* super enhancer^50^ reduced accessibility (Supplementary Fig. 3h).

By accessing published data on aged microglia^33^, we found that a subset of open promoter regions in aged microglia showed increased accessibility also in DAXX-deficient microglia (Supplementary Fig. 4a), whereas over 60% of intergenic regions gaining accessibility in DAXX-deficient microglia were also open in 24-month-old microglia (Supplementary Fig. 4b). Although ETS motifs were mainly enriched in aged microglia (Supplementary Fig. 4c), DAXX-deficient microglia presented enrichment in AP-1 motifs (Supplementary Fig. 4c), including in the above-mentioned intersected intergenic regions (Fig. 5f).

Collectively, these data suggest that DAXX-deficient microglia acquire a chromatin state compatible with both developmental and aging-associated cell identities.

### Loss of *Sall1* or viral mimicry signaling recapitulates some changes observed in DAXX-deficient microglia

Given the downregulation of homeostatic genes in DAXX-deficient microglia, we sought to examine potential transcriptional similarities between DAXX-deficient microglia and *Sall1* super enhancer-deficient microglia^50^ (germline *Sall1* heterozygous endogenous KO (EKO) and *Sall1* EKO; Supplementary Fig. 4d). Indeed, we could demonstrate a significant overlap in gene expression between DAXX-deficient and *Sall1*-EKO microglia (Supplementary Fig. 4d,e). We then integrated our data with bulk RNA-seq data from *Cx3cr1*-Cre^ERT2^; *Sall1*^fl/fl^ (*Sall1*-KO) mice^51^ and from in-house poly(I:C)-stimulated wild-type or *Ifnar*-KO microglia. We showed that the increase in cell cycle genes (1 d.p.i. to 1 w.p.i.) and C6/DAM signatures (3 d.p.i. to 1 y.p.i.), ISG induction (Fig. 5k) and downregulation of homeostatic genes in *Daxx*-KO microglia were in part recapitulated following deletion of *Sall1* itself^51^ or poly(I:C) treatment. Loss of *Ifnar* rescued homeostatic gene expression and reduced ISGs, whereas cell cycle genes were unaffected, and the gene encoding Ki67 and most C6/DAM genes were further upregulated (apart from *Apoe*, *Axl*, *Cd74* and *Ms4a6*).

Intersection of differentially upregulated and downregulated RTEs in microglia following LPS (ex vivo) or poly(I:C) (in vivo) treatment identified commonly upregulated and downregulated RTEs across conditions and time points (Supplementary Fig. 4f). Notably, a large proportion of these RTEs was upregulated in *Daxx-*KO microglia starting from 1 d.p.i. and peaking at 3 d.p.i. A similar induction was observed in *Sall1*-KO microglia as well as in poly(I:C)-stimulated control microglia (Supplementary Fig. 4g). However, most of these inflammation-related RTEs were not affected by *Ifnar* loss following poly(I:C) treatment.

These data indicate that gene and RTE expression changes caused by *Daxx* loss are in part recapitulated following loss of *Sall1* expression or poly(I:C) treatment.

### *Daxx* loss leads to reduced H3K9me3 at LTR-RTEs but no changes in H3.3 enrichment

**Fig. 6:**
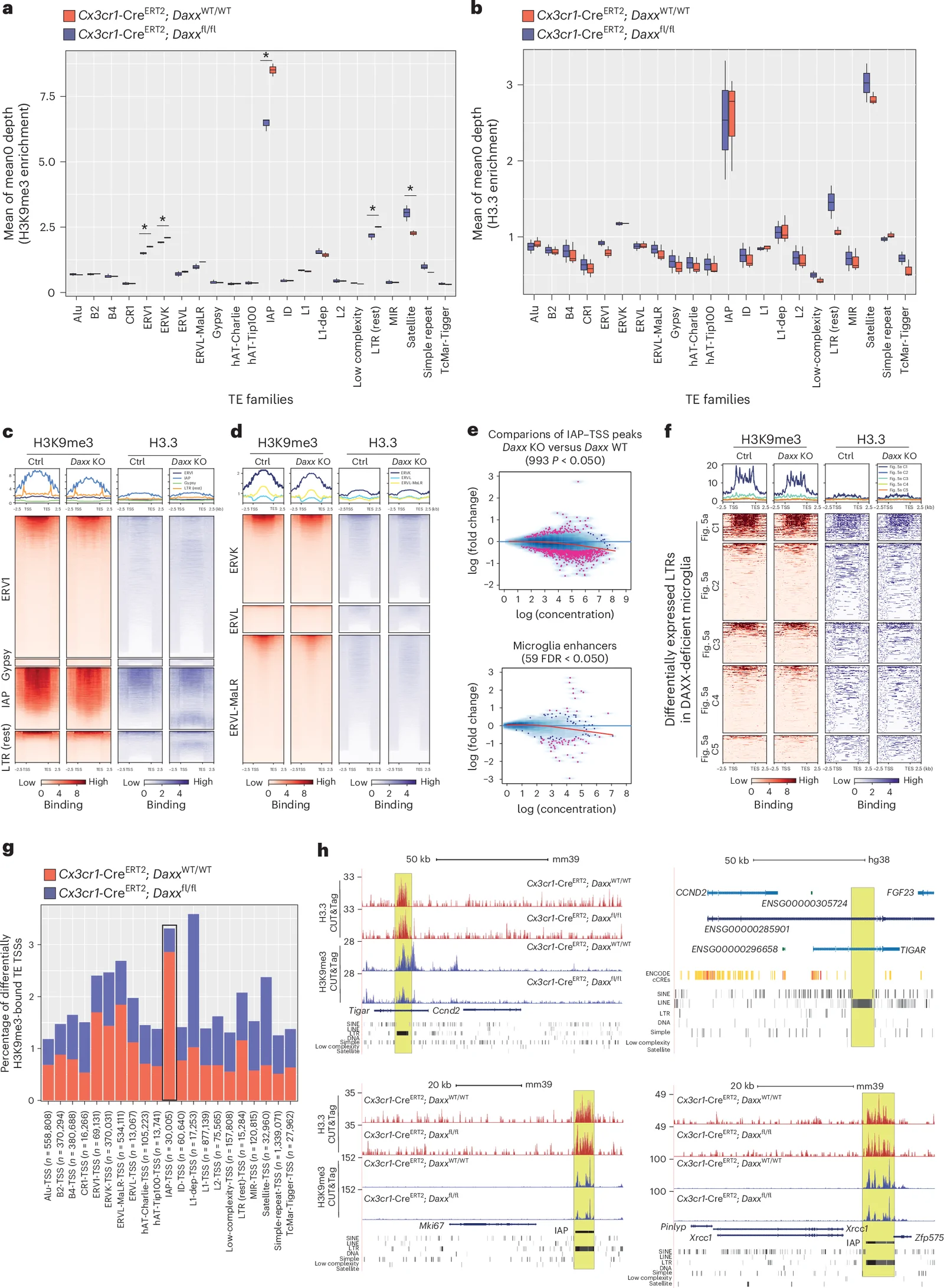
*Daxx* deletion causes reduced H3K9me3 at LTR-RTEs in adult microglia. **a**,**b**, Average H3K9me3 (**a**) or H3.3 (**b**) read depth across transposable element subfamilies in microglia isolated from *Cx3cr1*-Cre^ERT2^; *Daxx*^WT/WT^ mice (*n* = 2 mice for H3K9me3 and 3 mice for H3.3) and *Cx3cr1*-Cre^ERT2^; *Daxx*^fl/fl^ mice (*n* = 3 mice for H3K9me3 and 2 mice for H3.3). Read depth was quantified by 1× normalization (reads per genomic content) using University of California, Santa Cruz (UCSC)-tools bigWigAverageOverBed (v2). For each transposable element subfamily, the mean of mean0 (average over bases, with noncovered bases counting as zeroes) was calculated and visualized as box plots. Analysis included the 20 most abundant transposable element subfamilies in the mm39 UCSC RepeatMasker annotation, together with LTR subfamily Gypsy and IAP elements. Subsequent analysis focused on the transposable element subfamilies that were more prominently altered (in bold; *P* values were calculated using unpaired two-sided Student’s *t*-tests for *Cx3cr1*-Cre^ERT2^; *Daxx*^fl/fl^ versus *Cx3cr1*-Cre^ERT2^; *Daxx*^WT/WT^, *P*_ERV1_ = 0.0075, *P*_ERVK_ = 0.0122, *P*_IAP_ = 0.0273, *P*_LTR (rest)_ = 0.0349 and *P*_Satellite_ = 0.0344). Box plots show the median and first and third quartiles (hinges), and whiskers extend from the hinge to the smallest/largest value at most 1.5× the interquartile range. **c**,**d**, Heat maps and aggregate profiles of H3K9me3 and H3.3 enrichment across LTR subfamilies, including ERV1 (*n* = 69,131), Gypsy (*n* = 3,440), IAP (n = 30,005), ERVK (*n* = 370,031), ERVL (*n* = 113,067), ERVL-MaLR (*n* = 534,111) and LTR (rest; *n* = 15,284). IAP elements exhibited the strongest H3K9me3 enrichment and a marked reduction of H3K9me3 signal in *Cx3cr1*-Cre^ERT2^; *Daxx*^fl/fl^ microglia (*Daxx* KO) compared to *Cx3cr1*-Cre^ERT2^; *Daxx*^WT/WT^ microglia (control (Ctrl)). Different color keys show different marks (for example, H3.3, H3K9me3); TE, transposable element. **e**, MA plots showing binding changes using DiffBind. Top, IAP elements (*n* = 30,005) displaying a reduction in H3K9me3 (not H3.3) in *Daxx*-KO microglia versus control microglia. Bottom, human microglia-specific enhancers and promoters (data from ref. ^54^). Differential binding changes of homologs were assessed using DiffBind (FDR < 0.05; strict settings). Statistical analysis was performed using a Wald test on generalized linear model coefficients (from the DESeq2 package). **f**, Heat maps and profiles of H3K9me3 and H3.3 enrichment across LTR clusters identified in Fig. 5a. **g**, Bar plot showing the proportion of differentially bound H3K9me3 peaks overlapping TSSs of individual transposable element subfamilies. Percentages are calculated relative to the total number of TSSs in each subfamily. **h**, Genome browser views of representative loci showing reduced H3K9me3 enrichment and H3.3 occupancy at RTEs in *Daxx*-KO microglia. Asterisks (*) indicate statistical significance: *P < 0.05. Source data

Previous studies showed that the DAXX–TRIM28–SETDB1 complex plays a critical role in RTE silencing through induction of H3K9me3 (refs. ^4,52,53^) but that binding of DAXX to H3.3, rather than H3.3 deposition itself, appears to be required for this function, potentially via reciprocal regulation of their respective protein levels^4^ (Fig. 3b). CUT&Tag experiments on column-isolated *Cx3cr1*-Cre^ERT2^; *Daxx*^WT/WT^ and *Cx3cr1*-Cre^ERT2^; *Daxx*^fl/fl^ microglia (Fig. 6) revealed that *Daxx* loss reduces H3K9me3 at several LTR-RTEs, most prominently at intracisternal particles (IAPs; Fig. 6a). Although H3.3 was found at LTR-RTEs and often together with H3K9me3, it was mostly unaffected following *Daxx* loss (Fig. 6b–d). MA plot analysis illustrated two groups of differential IAP–transcription start site (IAP–TSS) peaks between DAXX-deficient and control microglia, with a wider distribution in the control group (Fig. 6e). Furthermore, we found differentially reduced H3K9me3 deposition at enhancers conserved between mice and humans^54^ in DAXX-deficient microglia (Fig. 6e). Importantly, by replotting deregulated LTR-RTEs (Fig. 5a), we observed reduced H3K9me3 marks at C1 in DAXX-deficient microglia (Fig. 6f), while H3.3 remained unchanged (Fig. 5a). By assessing the overall percentage of all LTR-RTEs, several subfamilies, including IAPs, displayed reduced H3K9me3 following *Daxx* loss (Fig. 6g). Interestingly, *Ccnd2* (encoding cyclin D2), *Mki67* and *Xrcc1* were upregulated following *Daxx* loss (Fig. 5k) and were found in proximity to H3.3/H3K9me3-marked LTR-RTEs, with H3K9me3 being reduced following *Daxx* loss (Fig. 6h). Motif analysis for IAP peaks with higher H3K9me3 binding showed enrichment in several specific motifs (Supplementary Fig. 5a).

Together, these findings suggest that DAXX regulates RTEs via H3K9 trimethylation without clear effects on H3.3 deposition.

### Gene expression changes caused by *Daxx* loss are in part recapitulated in H3.3- and TRIM28-deficient cells

Our findings suggest that DAXX interaction with H3.3, rather than its loading, is critical for RTE silencing in microglia, as previously reported^4^. Thus, we decided to investigate the overall effects of *Hira* or *H3f3* loss in microglia using TAM-inducible *H3f3a*/*H3f3b* and *Hira* conditional knockout mice. While microglial soma size was increased in the brains of *Rosa*-Cre^ERT2^; *H3f3a*^fl/fl^*H3f3b*^−/−^ mice compared to those in *H3f3a*^fl/fl^*H3f3b*^−/−^ control mice, microglia numbers were unchanged (Supplementary Fig. 5b,c). By contrast, microglia-specific loss of *Hira* had no effect on either microglia morphology or number (Supplementary Fig. 5d,e). Bulk RNA-seq data^55^ from the *H3f3a*/*H3f3b* double knockout (*H3.3*-DKO) RAW264.7 macrophage cell line (Supplementary Fig. 5f–h) showed that a number of genes that were altered in DAXX-deficient microglia were similarly affected in *H3.3*-DKO cells (Fig. 5c,k and Supplementary Fig. 5i,j). Interestingly, a number of ISGs were downregulated in *H3.3*-DKO cells (Supplementary Fig. 5j), suggesting that they may require H3.3 loading for expression. Finally, based on existing datasets^52,53^, TRIM28-deficient neural progenitor cells (NPCs) and lymphocytes also showed a partial overlap with genes altered in DAXX-deficient cells (Fig. 5k and Supplementary Fig. 5k).

In conclusion, our data suggest a functional interaction with H3.3 and the TRIM28–SETDB1 complex, as previously reported in other models.

### DAXX-deficient microglia enter into an aberrant state compatible with increased plasticity and DAM-like/*Apoe*^high^ features

**Fig. 7:**
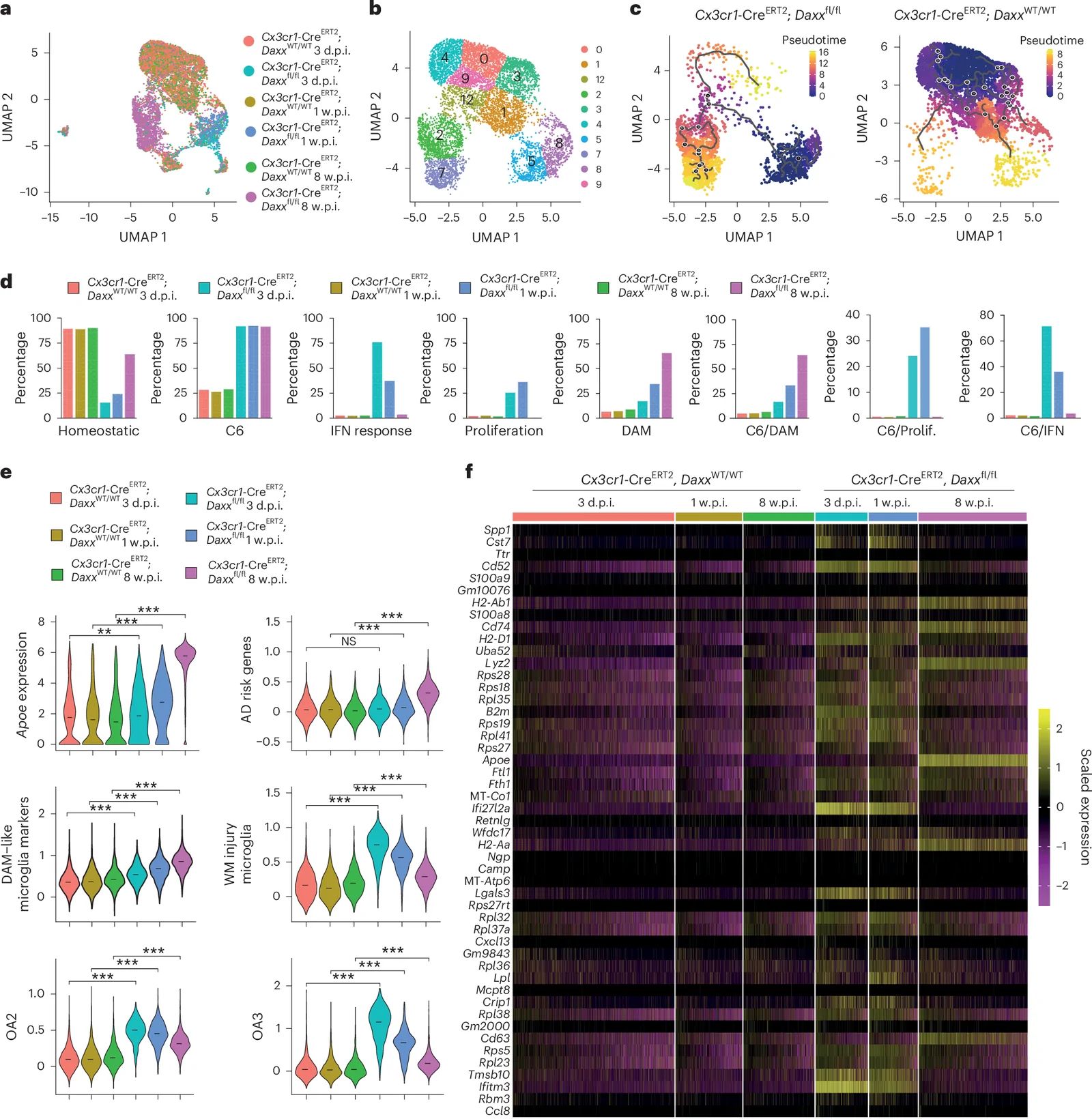
DAXX-deficient microglia enter into a transient state of enhanced plasticity, followed by acquisition of DAM-like features. **a**, Uniform manifold approximation and projection (UMAP) and graph-based clustering of in vivo microglia isolated from *Cx3cr1*-Cre^ERT2^; *Daxx*^WT/WT^ and *Cx3cr1*-Cre^ERT2^; *Daxx*^fl/fl^ mice at 3 d.p.i., 1 w.p.i. and 8 w.p.i. **b**, A microglia trajectory was constructed using 7,878 of the 8,922 total cells, focusing on the 10 most interconnected clusters. Each cluster was identified and color coded. **c**, Trajectory analysis of *Cx3cr1*-Cre^ERT2^; *Daxx*^WT/WT^ and *Cx3cr1*-Cre^ERT2^; *Daxx*^fl/fl^ microglia. **d**, Bar graphs showing the percentage of microglia bearing single or double phenotypes (homeostasis, C6, IFN response, proliferation and DAM) in *Cx3cr1*-Cre^ERT2^; *Daxx*^WT/WT^ and *Cx3cr1*-Cre^ERT2^; *Daxx*^fl/fl^ mice at 3 d.p.i., 1 w.p.i. and 8 w.p.i. **e**, Violin plots showing gene expression of *Apoe*, AD risk, DAM-like, white matter injury and aging microglia signatures (OA2 and OA3)^45^ in in vivo microglia isolated from the brains of *Cx3cr1*-Cre^ERT2^; *Daxx*^WT/WT^ and *Cx3cr1*-Cre^ERT2^; *Daxx*^fl/fl^ mice at 3 d.p.i., 1 w.p.i. and 8 w.p.i. The black line indicates the median expression/module score calculated using the AddModuleScore() function in Seurat. Statistical significance was assessed using pairwise two-sided Wilcoxon rank-sum tests and the geom_signif function (ggsignif R package) for the given comparisons, no correction for multiple testing; for *Apoe*, *P*_3 d.p.i._ = 0.0021, *P*_1 w.p.i._ < 2.22 × 10^−16^, *P*_8 w.p.i._ < 2.22 × 10^−16^; for AD risk genes, *P*_3 d.p.i._ = 0.12, *P*_1 w.p.i._ = 2.9 × 10^−6^, *P*_8 w.p.i._ < 2.22 × 10^−16^; for DAM-like markers, *P*_3 d.p.i._ < 2.22 × 10^−16^, *P*_1 w.p.i._ < 2.22 × 10^−16^, *P*_8 w.p.i._ < 2.22 × 10^−16^; for white matter injury microglia, *P*_3 d.p.i._ < 2.22 × 10^−16^, *P*_1 w.p.i._ < 2.22 × 10^−16^, *P*_8 w.p.i._ < 2.22 × 10^−16^; for OA2, *P*_3 d.p.i._ < 2.22 × 10^−16^, *P*_1 w.p.i._ < 2.22 × 10^−16^, *P*_8 w.p.i._ < 2.22 × 10^−16^; for OA3, *P*_3 d.p.i._ < 2.22 × 10^−16^, *P*_1 w.p.i._ < 2.22 × 10^−16^, *P*_8 w.p.i._ < 2.22 × 10^−16^). **f**, Heat map of the top 50 age-associated markers calculated from a published aging microglia data set^33^ across all cells of our scRNA-seq data set (*n* = 10,172 cells). The overlap of all age-associated markers with the markers from *Cx3cr1*-Cre^ERT2^; *Daxx*^fl/fl^ versus *Cx3cr1*-Cre^ERT2^; *Daxx*^fl/fl^ microglia (*n* = 492 cells; 68.05%; adjusted *P* < 0.05; average log_2_ (fold change > 0)) is highly significant: *P* = 2.2 × 10^−16^; odds ratio = 16.42 (two-sided Fisher’s exact test for count data). Asterisks (*) indicate statistical significance: **P < 0.01 and ***P < 0.001; MT, mitochondrial; WM, white matter. Source data

**Extended Data Fig. 4:**
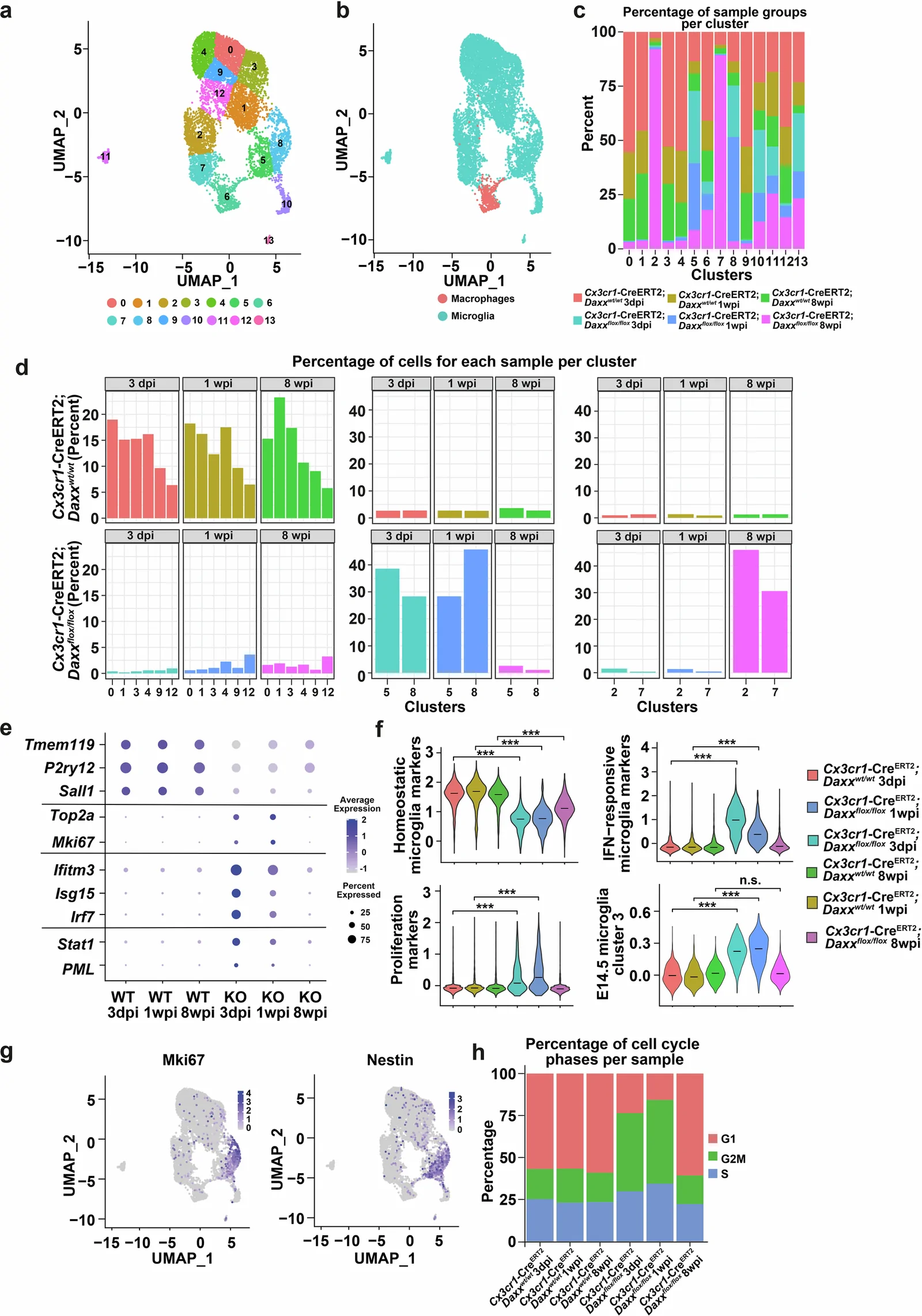
Single cell mRNA sequencing reveals distinct subpopulation of microglia associated with *Daxx* loss. **a**, UMAP plot showing 13 distinct clusters of microglia cells (8922 cells) identified with different colors. **b**, UMAP plot showing the distinct microglia and macrophage clusters isolated from *Cx3cr1*-Cre^ERT2^;*Daxx*^*wt/wt*^ and *Cx3cr1*-Cre^ERT2^;*Daxx*^*flox/flox*^ mice. **c**, Bar plot showing percentage of microglia distribution in each cluster identified in **a**. **d**, Bar plot showing percentage of microglia cells for each sample per cluster identified in **a**; The root clusters were chosen based on the highest percentage of cells at 3dpi – cluster 0 for *Cx3cr1*-Cre^ERT2^;*Daxx*^*wt/wt*^ microglia and cluster 5 for *Cx3cr1*-Cre^ERT2^;*Daxx*^*flox/flox*^ microglia. **e**, Dot plot showing homeostatic, proliferative and INF-response gene signatures for microglia isolated from each genotype. **f**, Violin plots showing gene expression of homeostatic, proliferation, INF-response markers and E14.5 cluster 3 signatures^46^ in *in vivo* microglia isolated from each genotype. The black line indicates the median expression/ module score calculated using the AddModuleScore() function in Seurat; (Statistical significance was assessed by pairwise two-sided Wilcoxon rank-sum tests using the geom_signif function (ggsignif R package) for the given comparisons, no correction for multiple testing; for Homeostatic microglia markers, *P*_3dpi_ < 2.22e-16, *P*_1wpi_ < 2.22e-16, *P*_8wpi_ < 2.22e-16; for IFN-responsive microglia markers, *P*_3dpi_ < 2.22e-16, *P*_1wpi_ < 2.22e-16, *P*_8wpi_ < 2.22e-16; for proliferation markers, *P*_3dpi_ < 2.22e-16, *P*_1wpi_ < 2.22e-16, *P*_8wpi_ = 1.7e-05; for E14.5 microglia cluster 3, *P*_3dpi_ < 2.22e-16, *P*_1wpi_ < 2.22e-16, *P*_8wpi_ < 2.22e-16; for OA2, *P*_3dpi_ < 2.22e-16, *P*_1wpi_ < 2.22e-16, *P*_8wpi_ < 2.22e-16; for OA3, *P*_3dpi_ < 2.22e-16, *P*_1wpi_ < 2.22e-16, *P*_8wpi_ = 0.86). **g**, Feature plots showing *Mki67* and *Nestin* gene expression in the microglial cells across all clusters. **h**, Bar plot showing percentage of cell cycle phases of microglia from *Cx3cr1*-Cre^ERT2^;*Daxx*^*wt/wt*^ and *Cx3cr1*-Cre^ERT2^;*Daxx*^*flox/flox*^ mice at 3dpi, 1wpi and 8wpi. Asterisks (*) indicate statistical significance: ***P < 0.001; n.s. indicates not significant. Source data

To address whether the different signatures arising following *Daxx* loss define specific microglia subpopulations and/or coexist in the same microglia cell, we performed longitudinal single-cell RNA-seq (scRNA-seq) of microglia following *Daxx* loss. We first removed clusters containing either mixed cell populations or nonmicroglial/nonmacrophage gene signatures and retained 13 clusters, of which only C6 showed a nonmicroglial macrophage-like signature (Fig. 7a and Extended Data Fig. 4a,b). DAXX-deficient microglia clustered separately from wild-type microglia at all time points, whereas 3 d.p.i. and 1 w.p.i. DAXX-deficient microglia clustered together but separately from 8 w.p.i. DAXX-deficient microglia (Fig. 7a). To further assess the dynamics of transcriptional changes, we constructed a microglia trajectory focusing on the most interconnected clusters (C0–C5, C7–C9 and C12; Fig. 7b) and built separate trajectories for control and DAXX-deficient cells (Fig. 7c) as the cell distribution within each cluster for the two genotypes, using root clusters based on the highest percentage of cells at 3 d.p.i. (Extended Data Fig. 4c,d). When cells were organized along each trajectory (pseudotime; Fig. 7c), DAXX-deficient microglia followed a trajectory starting from ISG^high^ cells, which first branched off toward Ki67^high^ cells (C8) and then toward *P2ry12*^high^*Sall1*^high^ cells (C1 and C12). This was followed by another bifurcation in C12, with cells either remaining *P2ry12*^high^*Sall1*^high^ (C9, C4, C0 and C3) or becoming *Apoe*^high^*H2*^high^ (C2 and C7). By contrast, control microglia retained mostly homeostatic features (*P2ry12*^high^*Sall1*^high^), with a subset of cells following a trajectory either toward Ki67^high^IFN^high^ (reactive) or *Apoe*^high^*H2*^high^ cells (DAMs). In line with bulk RNA-seq data, 3 d.p.i. and 1 w.p.i. microglia displayed loss of homeostatic markers and gain of ISG and proliferation/regeneration signatures (Extended Data Fig. 4e–g). Finally, 3 d.p.i. and 1 w.p.i. DAXX-deficient microglia resembled embryonic day 14.5 (E14.5) microglia^45^ and were enriched in G2/M phase genes, compatible with cell cycle arrest or enrichment in this phase (Extended Data Fig. 4f,h).

**Extended Data Fig. 5:**
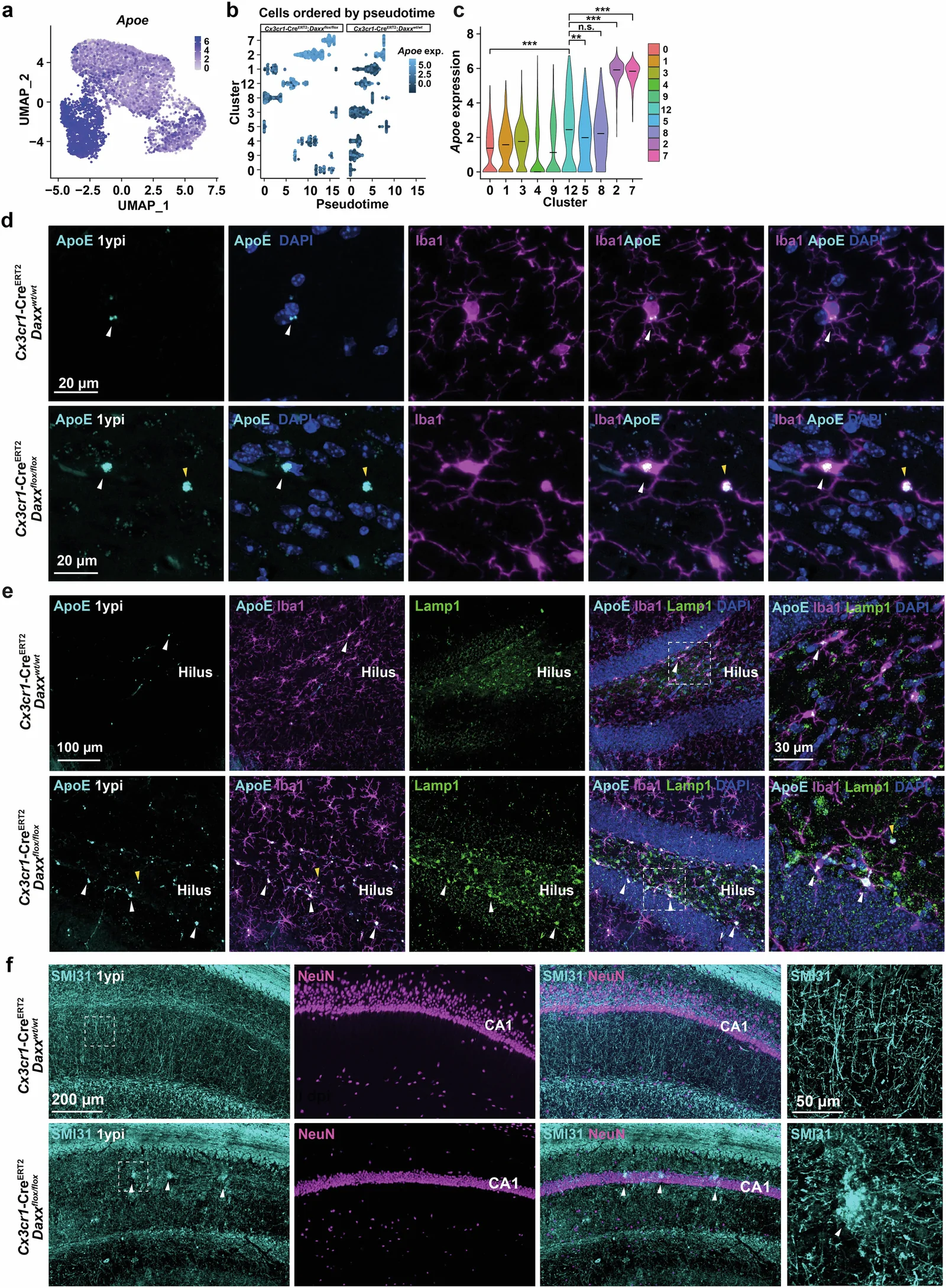
Loss of *Daxx* leads to lysosome-associated ApoE expression and dystrophic neurites in older mice. **a**, UMAP showing *Apoe* gene expression in the microglia across several clusters. **b**, Dot plot showing cells in each cluster ordered across Pseudotime and colored depending on their *Apoe* expression. **c**, violin plot showing *Apoe* gene expression in the microglia across all clusters. The black line indicates the median expression/ module score calculated using the AddModuleScore() function in Seurat; (Statistical significance was assessed by pairwise two-sided Wilcoxon rank-sum tests using the geom_signif function (ggsignif R package) for the given comparisons, no correction for multiple testing; *P*_12 vs 0_ < 2.22e-16, *P*_12 vs 5_ = 0.007, *P*_12 vs 8_ = 0.46, *P*_12 vs 2_ < 2.22e-16, *P*_12 vs 7_ < 2.22e-16). **d**, Representative confocal images of Iba1 and ApoE double immunostaining in the cortex of *Cx3cr1*-Cre^ERT2^;*Daxx*^*wt/wt*^ and *Cx3cr1*-Cre^ERT2^;*Daxx*^*flox/flox*^ mice at 1ypi (*n* = 3 mice for each genotype); White arrowheads indicate accumulated ApoE protein in microglia; yellow arrowheads indicate accumulated ApoE in dendrite of microglia. **e**, Representative confocal images of Iba1, ApoE and Lamp1 triple immunostaining in the hilus of *Cx3cr1*-Cre^ERT2^;*Daxx*^*wt/wt*^ and *Cx3cr1*-Cre^ERT2^;*Daxx*^*flox/flox*^ mice at 1ypi (*n* = 3 mice for each genotype); White arrowheads indicate the colocalization of ApoE and Lamp1 in the cell body of microglia; yellow arrowheads indicate the colocalization of ApoE and Lamp1 in the dendrite of microglia. **f**, Representative confocal images of SMI31 and NeuN double immunostaining in hippocampus of *Cx3cr1*-Cre^ERT2^;*Daxx*^*wt/wt*^ and *Cx3cr1*-Cre^ERT2^;*Daxx*^*flox/flox*^ mice at 1ypi (*n* = 3 mice for each genotype); Dashed boxes in the left panel identify high-magnification images of SMI31 staining (right panel); Arrowheads indicate the axonal aggregates. Asterisks (*) indicate statistical significance: **P < 0.01 and ***P < 0.001; n.s. indicates not significant. Source data

Although most DAXX-deficient microglia displayed reduced expression of homeostatic genes at 3 d.p.i. (84%), which were partially restored at 8 w.p.i., acquisition of the C6 signature was detected in most cells (92%) and was stable over time (Fig. 7d). The percentage of cells positive for the IFN signature was 77% at 3 d.p.i. and 37% at 1 w.p.i. in *Cx3cr1*-Cre^ERT2^; *Daxx*^fl/fl^ mice (Fig. 7d). Approximately 30% of total DAXX-deficient microglia displayed coexistence of C6/proliferation signatures at 3 d.p.i. and 1 w.p.i. (Fig. 7d). Furthermore, we found that the percentage of DAM^+^C6^+^ microglia increased from 17% at 3 d.p.i. to nearly 66% at 8 w.p.i. in *Cx3cr1*-Cre^ERT2^; *Daxx*^fl/fl^ mice, with 8 w.p.i. DAXX-deficient microglia also showing upregulation of major histocompatability complex class II (MHC II)-related genes (Fig. 7d and Supplementary Fig. 6a). Via integration of published data^45^, we showed that 3 d.p.i. and 1 w.p.i. DAXX-deficient microglia resembled injured white matter microglia and aged microglia (OA2 and OA3; Fig. 7e). Additionally, DAXX-deficient microglia also expressed C6 embryonic microglia genes, which comprise DAM and brain border-associated macrophage progenitor markers (Supplementary Fig. 6b). Of note, DAXX-deficient microglia progressively upregulated *Apoe* expression and the expression of other AD risk genes (Fig. 7e and Extended Data Fig. 5a). Based on pseudotime analysis (Extended Data Fig. 5b,c), reduced *Apoe* expression drove the trajectory toward *P2ry12*^high^*Sall1*^high^ cells (C0), whereas higher *Apoe* expression drove the trajectory toward *H2*^high^ cells (C2 and C7; Extended Data Fig. 5c). Finally, we found that the top 50 microglial genes from 24-month-old mice^33^ were enriched in the 3 d.p.i. and 1 w.p.i. DAXX-deficient microglia, of which some (for example, *Apoe* and *H2*) remained up until 8 w.p.i. (Fig. 7f).

Overall, these data suggest that DAXX deficiency leads to acquisition of an aberrant state displaying coexistence of reactive and DAM signatures, followed by the emergence of a stable DAM-like/*Apoe*^high^ state.

### *Daxx* downregulation leads to enhanced lysosomal ApoE expression and dystrophic neurites in older mice

To validate APOE induction at the protein level, we performed immunostaining and found that 1 y.p.i. DAXX-deficient microglia showed accumulation of structures containing ApoE and LAMP1 in both the cell body and processes (Extended Data Fig. 5d,e and Supplementary Fig. 7a), indicating alterations in phagocytosis. ApoE accumulation was not observed in GFAP^+^ cells (Supplementary Fig. 7b). We also showed SMI31^+^ aggregated neurites in the CA1 at 1 y.p.i. (Extended Data Fig. 5f). Finally, DAXX-deficient microglia displayed a transcriptional profile similar to amyloid plaque-associated microglia (XO4^+^)^56^ and late-onset AD microglia (Supplementary Fig. 7c).

**Extended Data Fig. 6:**
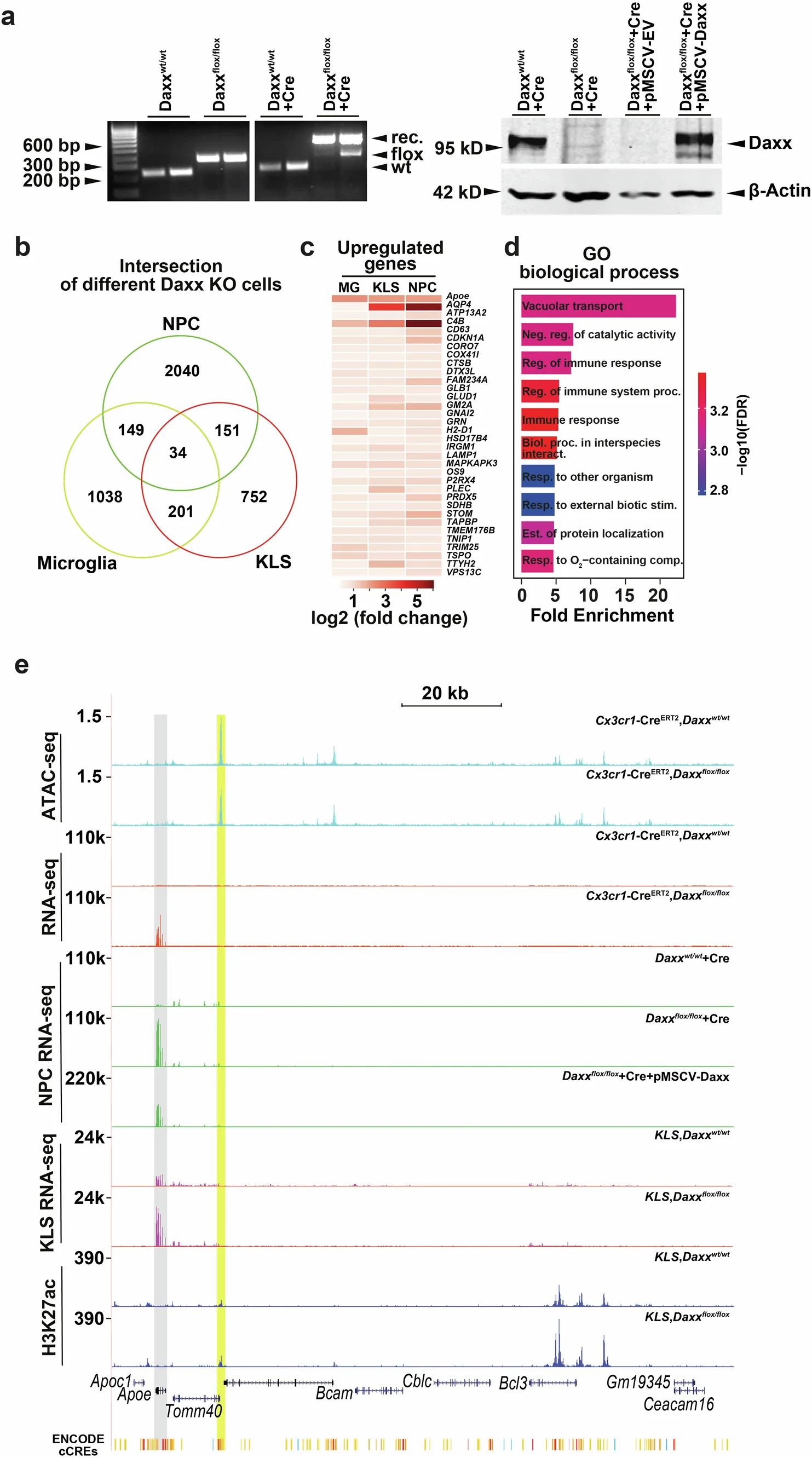
Changes in gene expression upon *Daxx* loss in microglia, NPC and KLS cells. **a**, Left panel: Genotyping of wild type and *Daxx*-floxed neuron progenitor cells (NPC) after transfection with the pMSCV-Cre construct (Cre recombinase) *ex vivo*. Arrows indicate the wild-type (226 bp), floxed (350 bp) and recombined alleles (600 bp); right panel: western blots showing Daxx protein expression after *Daxx* deletion or restoration of *Daxx* expression in *Daxx*-floxed NPCs (*n* = 3 independent preparations for each group). **b**, Venn diagram showing the overlap of differentially upregulated genes (DEGs) in *Cx3cr1*-Cre^ERT2^, *Daxx*^*flox/flox*^ microglia, *Daxx* knockout neuron progenitor cells (NPC) and *Daxx*-deficient bone marrow multipotent progenitors^27^ (KLS: c-kit^+^, Lin^−^, Sca-1^+^); The overlap of gene sets was evaluated using two-sided generalized hypergeometric test implemented in the *SuperExactTest* R package (*P* = 2.29e-15 for 34 upregulated genes). **c**, **d**, Heatmap and gene ontology analysis (GO) for 34 overlapping genes in **c**; Statistical significance of GO analysis was assessed using a two-sided hypergeometric test implemented in the clusterProfiler R package, and *P* values were adjusted for multiple testing using the Benjamini–Hochberg False Discovery Rate (FDR) procedure. **e**, Genome browser visualization of ATAC-seq, RNA-seq and ChIP-seq in *Daxx*-deficient cells and control cells around *Apoe* loci; Yellow region highlights the differential ATAC peaks and ChIP peaks overlapping with candidate cis-Regulatory Elements (cCREs); Brown region highlights *Apoe* loci. Source data

We found that 34 upregulated genes were shared across DAXX-deficient microglia, NPCs and bone marrow (KLS) progenitors^27^ (Extended Data Fig. 6a,b), including *Apoe*, *Lamp1* and *Cdkn1a* (Extended Data Fig. 6c), supported by GO analysis revealing vacuolar transport, cell cycle and immune terms (Extended Data Fig. 6d). Reintroduction of wild-type *Daxx* into DAXX-deficient NPCs led to reduced *Apoe* expression (Extended Data Fig. 6e). Finally, a number of *cis*-regulatory elements in the *Apoe* locus displayed increased acetylated H3K27 following *Daxx* loss in KLS progenitors (Extended Data Fig. 6e).

Together, these results suggest a potential direct role for DAXX in regulation of *Apoe* expression and a phagocytic gene program across cell types.

### *Daxx* loss causes induction of DNA damage markers, p53 targets and a senescence program

**Fig. 8:**
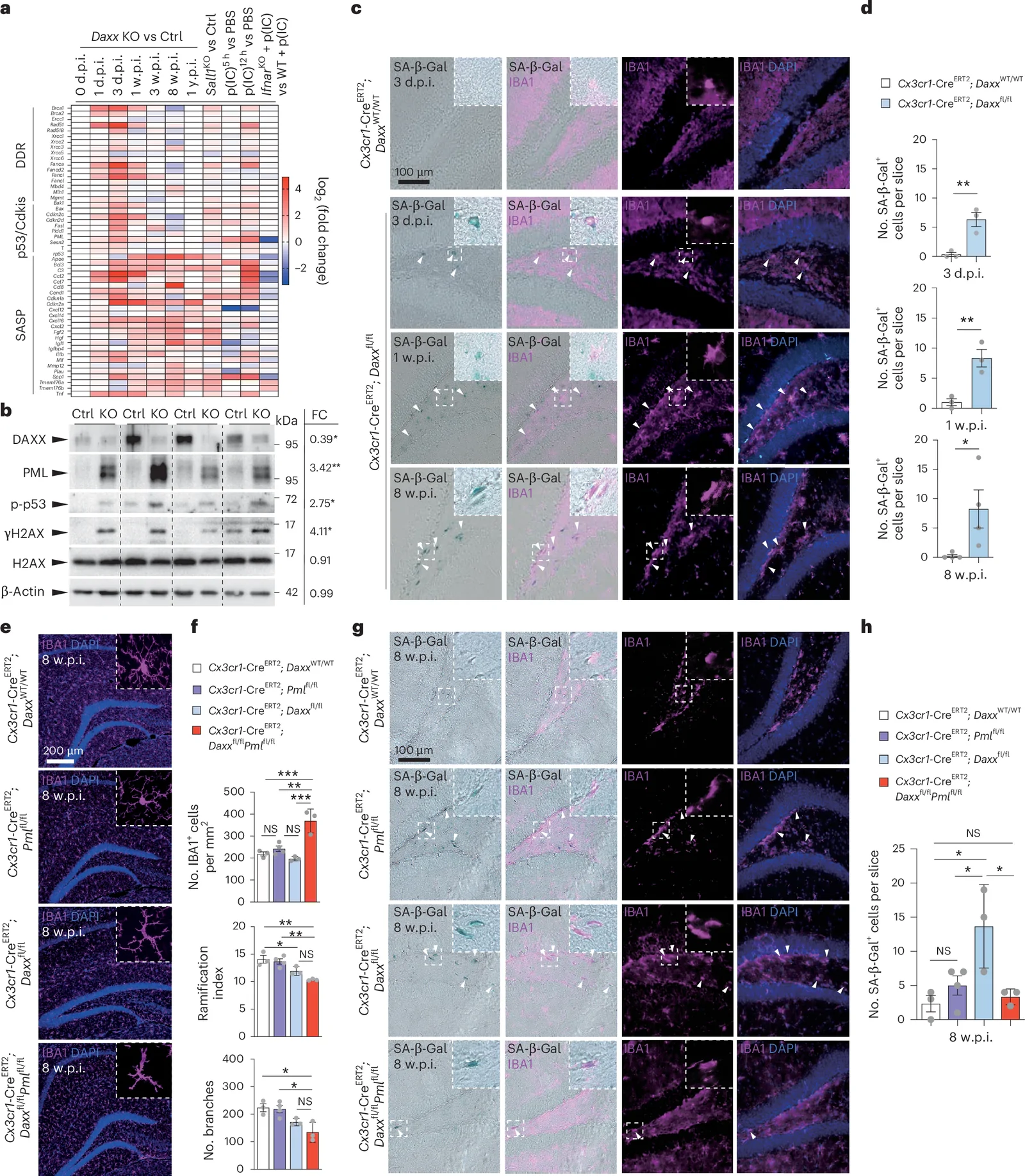
DAXX-deficient microglia present DNA damage and PML-dependent cellular senescence. **a**, Heat map showing the relative expression of gene signatures associated with distinct microglia states, including DDR, p53/Cdkis and senescence-associated secretory phenotype. Datasets include *Cx3cr1*-Cre^ERT2^; *Daxx*^fl/fl^ (*Daxx* KO) from 0 d.p.i. to 1 y.p.i., *Sall1*-Cre^ERT2^; *Sall1*^fl/fl^ (*Sall1* KO)^51^, poly(I:C)-stimulated wild-type microglia (p(IC)^5 h^, p(IC)^12 h^) and poly(I:C) stimulated *Ifnar*-KO microglia (*Ifnar* KO + p(IC)). **b**, Western blot and quantification of DAXX, PML, phospho-p53 (p-p53), γH2AX and H2AX in microglia isolated from *Cx3cr1*-Cre^ERT2^; *Daxx*^WT/WT^ (control) and *Cx3cr1*-Cre^ERT2^; *Daxx*^fl/fl^ (*Daxx*-KO) mice at 3 d.p.i. β-Actin served as the loading control (*n* = 4 mice per group; data were analyzed by two-tailed unpaired Student’s *t*-test, *P*_DAXX_ = 0.024, *P*_DAXX_ = 0.0054, *P*_p-p53_ = 0.0365, *P*γ_H2AX_ = 0.0197, *P*_H2AX_ = 0.2824, *P*_β-actin_ = 0.9935); FC, fold change. **c**,**d**, Representative confocal images (**c**) and quantification (**d**) of co-staining for SA-β-Gal activity and IBA1 in the hippocampus of *Cx3cr1*-Cre^ERT2^; *Daxx*^WT/WT^ and *Cx3cr1*-Cre^ERT2^; *Daxx*^fl/fl^ mice from 3 d.p.i. to 8 w.p.i. (3 d.p.i. and 1 w.p.i.: *n* = 3 mice for each genotype; 8 w.p.i.: *n* = 4 mice for each genotype; data were compared by two-tailed unpaired Student’s *t*-test, *P*_3 d.p.i._ = 0.0086, *P*_1 w.p.i._ = 0.0094, *P*_8 w.p.i._ = 0.0495). **e**,**f**, Representative confocal images of IBA1 immunostaining (**e**) and morphological quantification (**f**) of IBA1^+^ cells in the hippocampus of *Cx3cr1*-Cre^ERT2^; *Daxx*^WT/WT^ mice (*n* = 3 mice), *Cx3cr1*-Cre^ERT2^; *Pml*^fl/fl^ mice (*n* = 4 mice), *Cx3cr1*-Cre^ERT2^; *Daxx*^fl/fl^ mice (*n* = 3 mice) and *Cx3cr1*-Cre^ERT2^; *Daxx*^fl/fl^*Pml*^fl/fl^ mice (*n* = 3 mice) at 8 w.p.i. (data were analyzed by ordinary one-way ANOVA with a Tukey’s multiple comparisons test; for number of IBA1 cells: *F* = 19.10, *P*_WT versus *Pml* KO_ = 0.9207, *P*_WT versus *Daxx*/*Pml* DKO_ = 0.0012, *P*_*Pml* versus *Daxx*/*Pml* DKO_ = 0.0025, *P*_*Daxx* KO versus *Daxx*/*Pml* DKO_ = 0.0012; for ramification: *F* = 14.62, *P*_WT versus *Daxx* KO_ = 0.0281, *P*_WT versus *Daxx*/*Pml* DKO_ = 0.0011, *P*_*Pml* versus *Daxx*/*Pml* DKO_ = 0.0023, *P*_*Daxx* KO versus *Daxx*/*Pml* DKO_ = 0.1563; for number of branches: *F* = 8.073, *P*_WT versus *Daxx*/*Pml* DKO_ = 0.0114, *P*_*Pml* versus *Daxx*/*Pml* DKO_ = 0.0107, *P*_*Daxx* KO versus *Daxx*/*Pml* DKO_ = 0.3785). **g**,**h**, Representative confocal images (**g**) and quantification (**h**) of co-staining for β-galactosidase activity and IBA1 in the hippocampus of *Cx3cr1*-Cre^ERT2^; *Daxx*^WT/WT^ mice (*n* = 3 mice), *Cx3cr1*-Cre^ERT2^; *Pml*^fl/fl^ mice (*n* = 4 mice), *Cx3cr1*-Cre^ERT2^; *Daxx*^fl/fl^ mice (*n* = 3 mice) and *Cx3cr1*-Cre^ERT2^; *Daxx*^fl/fl^*Pml*^fl/fl^ mice (*n* = 3 mice) at 8 w.p.i. (data were analyzed by ordinary one-way ANOVA, *F* = 6.6, *P* = 0.0119; Tukey’s multiple comparisons test: *P*_WT versus *Pml* KO_ = 0.754, *P*_WT versus *Daxx* KO_ = 0.0142, *P*_WT versus *Daxx*/*Pml* DKO_ = 0.9843, *P*_*Daxx* KO versus *Pml* KO_ = 0.0415, *P*_*Daxx* KO versus *Daxx*/*Pml* DKO_ = 0.0237). Data are presented as mean ± s.e.m. Asterisks (*) indicate statistical significance: *P < 0.05, **P < 0.01 and ***P < 0.001. Source data

**Extended Data Fig. 7:**
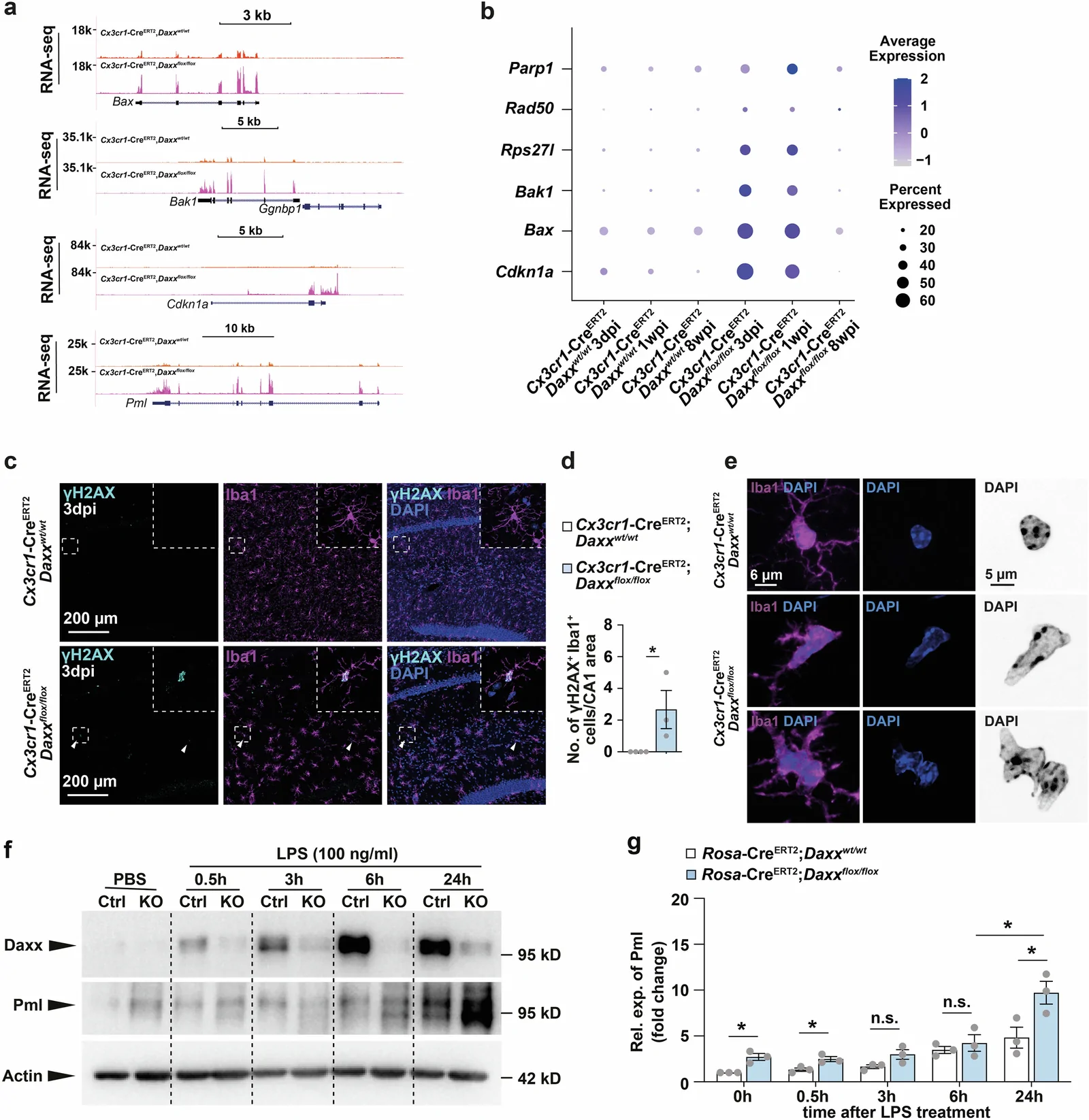
*Daxx* deficiency causes upregulation of apoptotic genes, elevated γH2AX and abnormal nuclear morphology in microglia. **a**, Genome browser tracks of RNA-seq at gene loci for *Bax*, *Bak1*, *Cdkn1a*, *Pml* in microglia isolated from *Cx3cr1*-Cre^ERT2^;*Daxx*^*wt/wt*^ and *Cx3cr1*-Cre^ERT2^;*Daxx*^*flox/flox*^ mice at 3dpi. **b**, Dot plot showing gene expression of *Parp1*, *Rad50*, *Rps27l*, *Bak1*, *Bax* and *Cdkn1a* in microglia isolated from each genotype. **c**, **d**, Representative confocal images and quantification of Iba1 and γH2AX double immunostaining in brain sections of *Cx3cr1*-Cre^ERT2^;*Daxx*^*flox/flox*^ (*n* = 4 mice) and *Cx3cr1*-Cre^ERT2^;*Daxx*^*wt/wt*^ mice (*n* = 3 mice) at 3dpi (comparison by two-tailed unpaired Student’s *t* test, *P* = 0.0453). **e**, Representative confocal images of DAPI and Iba1 immunostaining display abnormal nucleus morphology in *Cx3cr1*-Cre^ERT2^;*Daxx*^*flox/flox*^ microglia (*n* = 3 mice for each genotype). **f**, **g**, Western blot and quantification analysis of Daxx and Pml in *Rosa*-Cre^ERT2^;*Daxx*^*flox/flox*^ BMDMs (KO) and *Rosa*-Cre^ERT2^;*Daxx*^*wt/wt*^ BMDMs (Ctrl) after 0.5h-, 3h-, 6h- and 24h-LPS stimulation (*n* = 3 independent preparations per group, comparison by two-way RM ANOVA *F* = 4.174, *P* = 0.0167, Tukey’s multiple comparisons test: *P*_0h Daxx WT vs 0h Daxx KO_ = 0.0448, *P*_0.5h Daxx WT vs 0.5h Daxx KO_ = 0.0307, *P*_3h Daxx WT vs 3h Daxx KO_ = 0.1152, *P*_6h Daxx WT vs 6h Daxx KO_ = 0.5129, *P*_24h Daxx WT vs 24h Daxx KO_ = 0.0449, *P*_6h Daxx KO vs 24h Daxx KO_ = 0.0174). β-actin was used as an internal loading control. Data are presented as mean ± s.e.m. Asterisks (*) indicate statistical significance: *P < 0.05, **P < 0.01 and ***P < 0.001; n.s. indicates not significant. Source data

We sought to explore the potential association between the cell death phenotype and loss of genomic integrity. Indeed, *Cx3cr1*-Cre^ERT2^; *Daxx*^fl/fl^ microglia displayed increased DNA damage response (DDR)/p53/Cyclin-dependent kinase inhibitors (Cdkis) signatures between 1 d.p.i. and 1 w.p.i. (Fig. 8a), whereas p53 target genes involved in apoptosis (*Bax* and *Bak*), growth arrest/senescence (*Cdkn1a* and *Pml*) and the senescence-associated secretory phenotype were induced from 1 d.p.i. and remained higher until 1 y.p.i. (Fig. 8a and Extended Data Fig. 7a). Similar changes were observed following *Sall1* loss or poly(I:C) stimulation, but most of the DDR genes and p53 targets were not affected by *Ifnar* loss. Accordingly, our scRNA-seq data also showed higher levels of cell cycle, apoptosis and DNA repair genes (Extended Data Fig. 7b). Increased expression of γH2AX, phosphorylated p53 and the prosenescence and type 1 IFN/p53 target PML protein were detected in 3 d.p.i. DAXX-deficient microglia (Fig. 8b), correlating with an increased percentage of γH2AX^+^IBA1^+^ cells and abnormal microglial nuclear morphology (Extended Data Fig. 7c–e). No DNA damage or DDR was observed in either Cre^+^ wild-type controls or DAXX-deficient cells at 0 d.p.i. (Fig. 8a), arguing against Cre or TAM artifactual effects. Finally, *Daxx* loss led to an increase in senescence-associated β-galactosidase^+^ (SA-β-Gal^+^) microglia, substantiating our gene expression data (Fig. 8c,d).

### Induction of senescence following *Daxx* ablation is dependent on the nuclear scaffold protein PML

**Extended Data Fig. 8:**
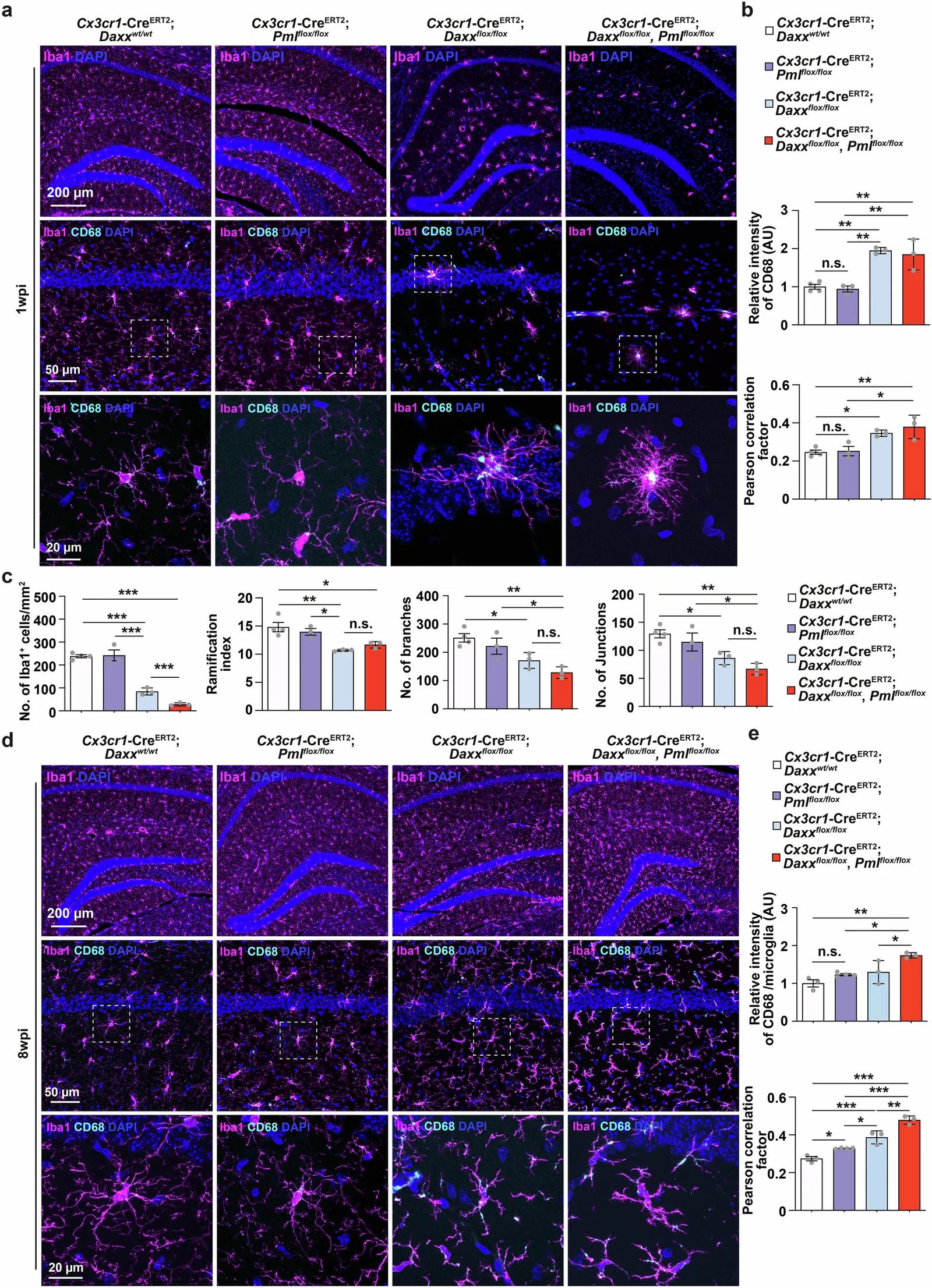
Effect of combined loss of *Daxx* and *Pml* on microglia morphology and activation markers. **a**-**c**, Representative confocal images, quantification of Iba1 and CD68 immunostaining and morphological quantification of Iba1^+^ cells in the hippocampus of *Cx3cr1*-Cre^ERT2^;*Daxx*^*wt/wt*^ (WT, *n* = 4 mice), *Cx3cr1*-Cre^ERT2^;*Pml*^*flox/flox*^ (Pml KO, *n* = 3 mice), *Cx3cr1*-Cre^ERT2^;*Daxx*^*flox/flox*^ (Daxx KO, *n* = 3 mice) and *Cx3cr1*-Cre^ERT2^;*Daxx*^*flox/flox*^,*Pml*^*flox/flox*^ (DKO, *n* = 3 mice) at 1wpi (Comparison by ordinary one-way ANOVA with Tukey’s multiple comparisons test: for CD68 intensity, *F* = 19.57, *P*_WT vs Daxx KO_ = 0.0013, *P*_WT vs DKO_ = 0.0029; for Pearson correlation, *F* = 9.74, *P*_WT vs Daxx KO_ = 0.0337, *P*_WT vs Daxx/Pml DKO_ = 0.0066); for Iba1^+^ cells, *F* = 76.04, *P*_WT vs Daxx KO_ < 0.0001, *P*_Daxx KO vs DKO_ < 0.0001; for Ramification, *F* = 9.952, *P*_WT vs Daxx KO_ = 0.0042, *P*_Daxx KO vs Pml KO_ = 0.0245; for branches, *F* = 8.925, *P*_WT vs Daxx KO_ = 0.0485, *P*_Pml KO vs DKO_ = 0.0313; for junctions, *F* = 9.137, *P*_WT vs Daxx KO_ = 0.037, *P*_Pml KO vs DKO_ = 0.0305). **d**, **e**, Representative confocal images and quantification of Iba1 and CD68 immunostaining in the hippocampus of *Cx3cr1*-Cre^ERT2^;*Daxx*^*wt/wt*^ (*n* = 4 mice), *Cx3cr1*-Cre^ERT2^;*Pml*^*flox/flox*^ (*n* = 3 mice), *Cx3cr1*-Cre^ERT2^;*Daxx*^*flox/flox*^ (*n* = 3 mice), and *Cx3cr1*-Cre^ERT2^;*Daxx*^*flox/flox*^,*Pml*^*flox/flox*^ (*n* = 3 mice) at 1wpi. Bottom panels showing high-magnification images in dashed box. (Comparison by ordinary one-way ANOVA with Tukey’s multiple comparisons test: for CD68 intensity, *F* = 9.915, *P*_WT vs Pml KO_ = 0.3467, *P*_Daxx KO vs DKO_ = 0.0471; for Pearson correlation, *F* = 51.14, *P*_WT vs Pml KO_ = 0.0361, *P*_WT vs Daxx KO_ = 0.0006, *P*_Daxx KO vs DKO_ = 0.0024, *P*_Pml KO vs DKO_ < 0.0001). Data are presented as mean ± s.e.m. Asterisks (*) indicate statistical significance: *P < 0.05, **P < 0.01 and ***P < 0.001; n.s. indicates not significant. Source data

We next sought to investigate potential mechanisms underlying induction of cellular senescence following *Daxx* loss. PML, a DAXX interactor involved in regulation of senescence and the essential component of PML nuclear bodies^57–59^, was induced following *Daxx* ablation in microglia and BMDMs (Fig. 8b and Extended Data Fig. 7a,f,g). While *Cx3cr1*-Cre^ERT2^-mediated deletion of *Pml* alone did not alter microglial number or morphology (Fig. 8e,f and Extended Data Fig. 8a,b), the hippocampus of TAM-treated *Cx3cr1*-Cre^ERT2^; *Daxx*^fl/fl^; *Pml*^fl/fl^ (*Daxx*/*Pml* DKO) mice at 8 w.p.i. showed increased numbers of microglia and CD68 expression and reduced ramification index, suggesting a more reactive phenotype (Fig. 8e,f and Extended Data Fig. 8a,c–e). Moreover, Ki67^+^ microglia were increased in *Daxx*/*Pml* DKO mice compared to in other genotypes at 1 w.p.i., but not later (Supplementary Fig. 8a–d). Finally, at 8 w.p.i., the number of SA-β-Gal^+^ cells in *Daxx*/*Pml* DKO mice was reduced compared to in *Cx3cr1*-Cre^ERT2^; *Daxx*^fl/fl^ mice (Fig. 8e–h).

These findings suggest that PML limits the expansion of replenishing microglia following *Daxx* loss via maintenance of a senescence phenotype.

### *Daxx* overexpression inhibits ApoE expression and cellular senescence in a replicative senescence model

**Extended Data Fig. 9:**
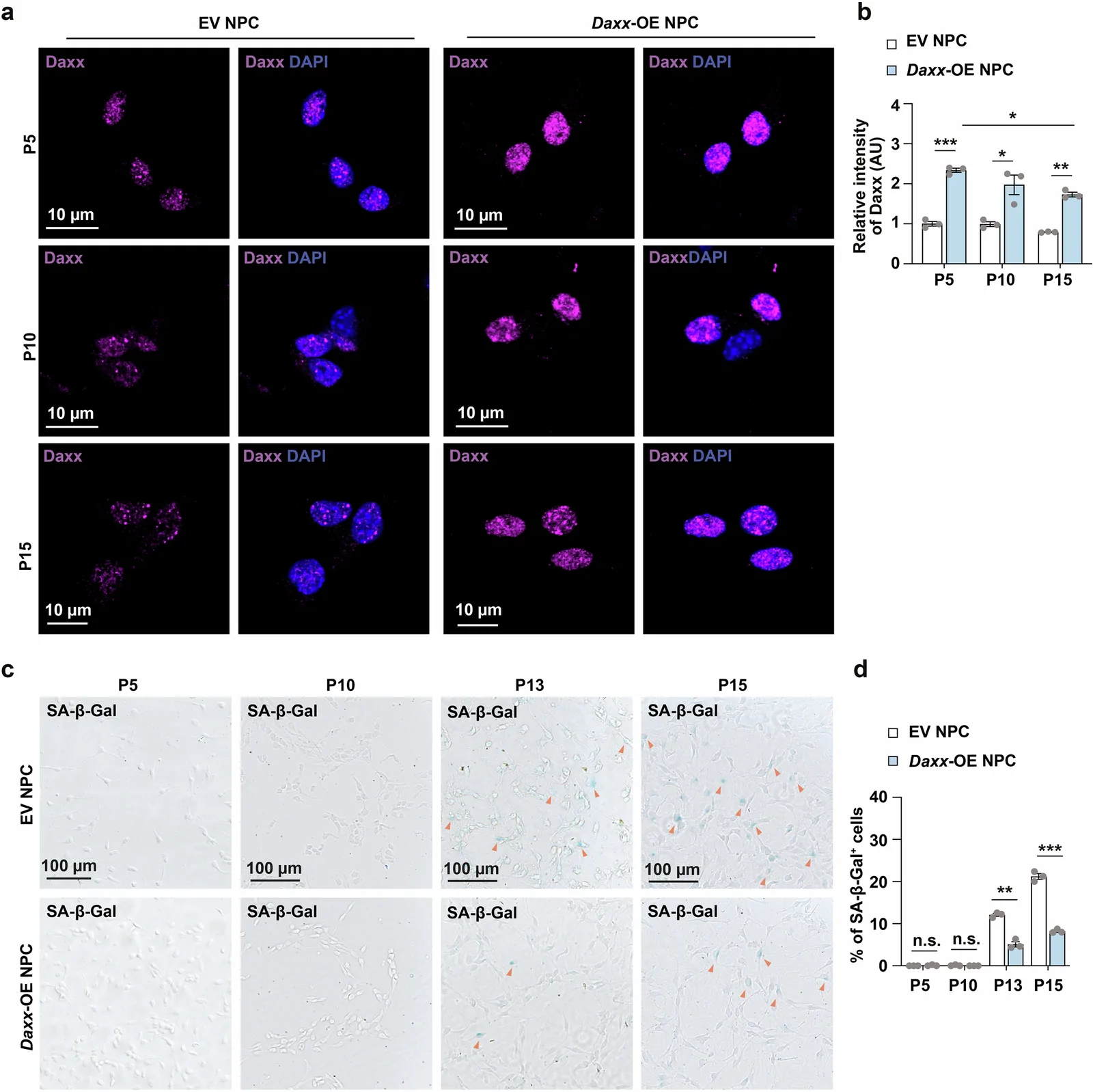
Elevating Daxx expression in neural stem/progenitor cells inhibits cell senescence. **a**, **b**, Representative confocal images of Daxx and quantification of Daxx intensity in the cultured neural stem/progenitor cells (NPCs) transfected with empty vector (EV NPCs) or flag-*Daxx* lentiviruses (*Daxx*-overexpression (OE) NPCs) at passage 5 (P5), P10 and P15 (*n* = 3 independent preparations per group, comparison by two-way RM ANOVA *F* = 2.574, *P* = 0.1371; Sidak’s multiple comparisons test:, *P*_P5 EV vs P5 Daxx-OE_ < 0.0001, *P*_P10 EV vs P10 Daxx-OE_ = 0.0497, *P*_P15 EV vs P15 Daxx-OE_ = 0.0034, *P*_P5 Daxx-OE vs P15 Daxx-OE_ = 0.036). **c**, **d**, Representative confocal images and quantification of beta-galactosidase activity in the NPCs at P5, P10, P13 and P15 (*n* = 3 independent preparations per group, comparison by two-way RM ANOVA, *F* = 117.7, *P* < 0.0001; Sidak’s multiple comparisons test: *P*_P5 EV vs P5 Daxx-OE_ = 0.4226, *P*_P10 EV vs P10 Daxx-OE_ = 0.4226, *P*_P13 EV vs P13 Daxx-OE_ = 0.002, *P*_P15 EV vs P15 Daxx-OE_ = 0.0009). Data are presented as mean ± s.e.m. Asterisks (*) indicate statistical significance: *P < 0.05, **P < 0.01 and ***P < 0.001; n.s. indicates not significant. Source data

**Extended Data Fig. 10:**
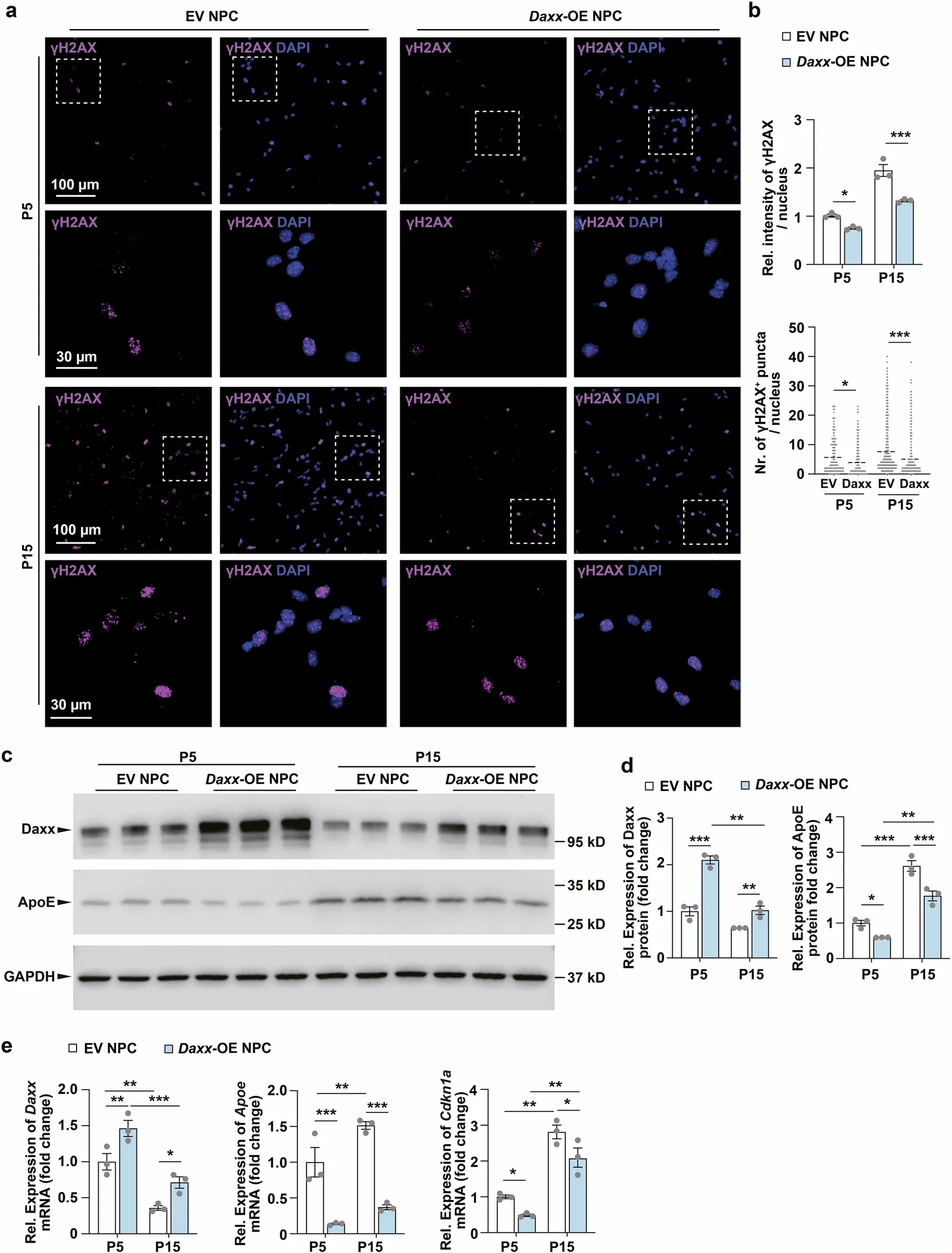
Exogenous Daxx reduces γH2AX, *Apoe* and *Cdkn1a* expression in neural stem/progenitor cells. **a**, **b**, Representative confocal images of γH2AX and quantification of γH2AX signal in the cultured neural progenitor cells (NPCs) transfected with empty vector (EV NPCs) or flag-*Daxx* lentiviruses (*Daxx*-OE NPCs) at passage 5 (P5) and P15 (for intensity of γH2AX, *n* = 3 independent preparations per group, comparison by two-way RM ANOVA with Sidak’s multiple comparisons test: *F* = 12.84, *P*_P5 EV vs Daxx-OE_ = 0.0443, *P*_P15 EV vs Daxx-OE_ = 0.0003; for γH2AX puncta, analysis included 172 nucleus (P5 EV), 331 nucleus (P5 *Daxx*-OE), 1081 nucleus (P15 EV), and 433 nucleus (P15 *Daxx*-OE) from 3 independent preparations per group, comparison by two-way RM ANOVA with Tukey’s multiple comparisons test: *F* = 35.89, *P*_P5 EV vs Daxx-OE_ = 0.0407, *P*_P15 EV vs Daxx-OE_ < 0.0001). **c**, **d**, Western blot analysis and quantification of Daxx and ApoE proteins expression in lysates of cultured NPCs at P5 and P15 (*n* = 3 independent preparations per group, comparison by two-way RM ANOVA with Fisher’s LSD multiple comparisons test, for Daxx: *F* = 13.82, *P*_P5 EV vs Daxx-OE_ < 0.0001, *P*_P15 EV vs Daxx-OE_ = 0.0098; for ApoE: *F* = 5.853, *P*_P5 EV vs Daxx-OE_ = 0.031, *P*_P15 EV vs Daxx-OE_ = 0.0006). **e**, qPCR for genes expressions in NPCs at P5 and P15 (*n* = 3 independent preparations per group, comparison by two-way RM ANOVA with Fisher’s LSD multiple comparisons test, for *Daxx*: *F* = 36.28, *P*_P5 EV vs Daxx-OE_ = 0.0069, *P*_P15 EV vs Daxx-OE_ = 0.001; for *Apoe*: *F* = 1.709, *P*_P5 EV vs Daxx-OE_ = 0.0005, *P*_P15 EV vs Daxx-OE_ < 0.0001; for *Cdkn1a*: *F* = 0.56, *P*_P5 EV vs Daxx-OE_ = 0.0481, *P*_P15 EV vs Daxx-OE_ = 0.0101). Data are presented as mean ± s.e.m. Asterisks (*) indicate statistical significance: *P < 0.05, **P < 0.01 and ***P < 0.001; n.s. indicates not significant. Source data

We decided to explore whether DAXX is sufficient to inhibit cellular senescence using a replicative senescence model based on extended culturing of embryonic NPCs infected with a *Daxx* lentiviral vector (*Daxx*-OE NPCs) or an empty vector (EV NPCs; Extended Data Fig. 9a,b). DAXX levels decreased with increasing passages in both EV and *Daxx*-OE NPCs but remained higher in *Daxx*-OE cells (Extended Data Fig. 9a,b). While the percentage of SA-β-Gal^+^ cells, γH2AX intensity and ApoE expression gradually increased in EV NPCs through passaging, these markers were reduced in *Daxx*-OE NPCs from postnatal day 13 (P13) onward (Extended Data Figs. 9c,d and 10a–d), along with *Apoe* and *Cdkn1a* (Extended Data Fig. 10e).

Collectively, these data indicate that overexpression of *Daxx* is sufficient to inhibit cellular senescence in NPCs and to suppress *Apoe* expression in a replicative senescence model.

## Discussion

In the present study, we link maintenance of chromatin compaction at RTEs by the H3.3 chaperone DAXX to regulation of microglia identity and function in the adult brain. An inverse correlation between DAXX and RTE expression was demonstrated in peripheral blood samples of a large group of community-dwelling individuals, validating the RTE-repressive role of DAXX in human settings. Longitudinal assessment of these individuals displaying increased RTE levels would be an obvious future development.

We advance a model whereby DAXX represses RTE repression at steady state in microglia, whereas following exposure to activating stimuli, DAXX is induced in an IFN-dependent manner to re-silence RTEs and restrict the inflammatory response. In agreement with this model, loss of DAXX causes chronic RTE derepression and aberrant proinflammatory signaling, with implications for immune cell function and tissue homeostasis. Furthermore, we propose that DAXX contributes to the epigenetic barrier insulating microglia cell identity in the adult. In this respect, *Daxx* loss leads to a transient state, where multiple gene expression signatures coexist within the same cell, potentially reflecting enhanced plasticity. In agreement with our findings, recent studies suggest that replenishment of microglia in depletion models is due to reversion to a state permissive for expansion^60^. How proliferation and cell death are functionally related in adult microglia remains poorly understood. Based on our findings, we envision that microglia carrying chromatin decompaction lose homeostatic features via lowering of the epigenetic barrier and entry into a developmental-like state characterized by cell cycle entry. However, most of these microglia would be unable to complete cell division due to induction of DNA damage, leading to cell death or cellular senescence, as observed in the context of replication/mitotic stress following oncogenic activation^61^. One could also speculate that due to aging-associated loss of heterochromatin, microglia would be more susceptible to DNA damage, especially when they respond to stimuli such as inflammation, which would promote cell cycle entry. Notably, cellular senescence has been associated with microglia dysfunction during aging also in other studies^31,33^, whereas loss of heterochromatin itself can induce cellular senescence^62^. Finally, HERVK elements have been suggested to reinforce cellular senescence during aging^8^. What remains to be understood is what the source of replenishment of the microglia pool is following *Daxx* loss. In this respect, we cannot exclude that migration of microglia from other regions contributes to this phenomenon, as suggested by others with respect to border-associated macrophages^63^.

We then showed that the DAXX-interacting and IFN-responsive factor PML is responsible for sustained maintenance of the senescence phenotype following *Daxx* deletion. In agreement with this, concomitant DAXX and PML inactivation in microglia led to increased microglia cell numbers after the replenishment phase. However, further, potentially intertwined, mechanisms might also be involved. In this respect, recent studies showed that secreted ApoE induces senescence in neurons and neutrophils, potentially via a TREM2-dependent mechanism^64,65^. Notably, ApoE promotes degradation of TRIM28 and destabilization of heterochromatin for induction of senescence^66^. Further investigation is required to explore the functional involvement of ApoE in regulation of microglial heterochromatin, as well as its potential interplay with PML.

Would the acquisition of DAM-like and senescence features in our model have a detrimental or protective impact on brain homeostasis? On one hand, microglial DAXX loss leads to changes in mouse behavior, which are compatible with an anxiety-like phenotype. This is consistent with a recent report implicating DAXX as risk factor for anxiety disorders in humans^67^. With respect to aging and aging-associated diseases, ApoE protein colocalized with the lysosomal marker LAMP1 in DAXX-deficient microglia, a phenotype also observed around plaques in an AD model. These structures may also constitute phagocytosed dystrophic neurites, which have been shown to be positive for LAMP1 in AD models^68^. These changes are associated with the presence of what appear to be axonal aggregates positive for phosphorylated neurofilament, suggesting altered protein quality control and/or phagocytosis. Furthermore, a previous study showed that microglia positive for ARG1 and ApoE also display phagocytic inclusions, like in DAXX-deficient cells^69^. Interestingly, we observed induction of LAMP1 along with genes involved in vesicle trafficking following *Daxx* loss also in NPCs and hematopoietic progenitors, suggesting that altered phagocytic function is a key consequence of DAXX impairment. A DAXX–lysosome molecular axis is of interest considering that alterations of lysosomes are fundamental events in aging and aging-associated conditions^70^. Finally, the emerging role of both DAXX and PML^71–73^ in inhibition of protein aggregation might also contribute to maintenance of (nuclear) proteostasis in microglia during aging, a further line of research arising from our study.

Overall, our work provides evidence for heterochromatin maintenance and RTE silencing as epigenetic barriers against perturbations of microglial cell identity and induction of senescence, with implications for our understanding of microglia biology in health and disease.

## Methods

### Measurements of *DAXX* and RTEs levels in humans

#### Study population

We used data from participants of the Rhineland Study, an on-going population-based cohort study in Bonn, Germany. Invitations to participate in the Rhineland Study are extended to all inhabitants of two geographically defined areas of Bonn who meet the inclusion criteria: aged 30 years and older and sufficient command of the German language to give informed consent. The mean (s.d.) age was 55.40 (14.48) years, and 45.4% were men (see Source Data Fig. 1). The study was approved by the ethics committee of the University of Bonn, Medical Faculty. The Rhineland Study is carried out in accordance with the recommendations of the International Conference on Harmonization Good Clinical Practice standards. Written informed consent was obtained from all participants in accordance with the Declaration of Helsinki.

#### Whole-blood RNA isolation and sequencing

Total RNA was isolated from 3,480 peripheral blood samples. The blood was collected in PAXgene Blood RNA tubes (PreAnalytix/Qiagen) and processed in accordance with the manufacturer’s instructions. Total RNA was extracted following the manufacturer’s guidelines using a PAXgene Blood miRNA kit. The integrity and quantity of RNA were assessed using a TapeStation RNA assay on a 4200 TapeStation system (Agilent Technologies). We used 750 ng of total RNA to generate next-generation sequencing libraries using the TruSeq stranded total RNA kit (Illumina) following the manufacturer’s instructions with Ribo-Zero Globin reduction. Library size distribution was assessed via D1000 ScreenTape assay on a Tapestation 4200 system (Agilent), and library quantification was performed using a Qubit HS dsDNA assay (Invitrogen). The libraries were clustered at a final concentration of 250 pM on a NovaSeq6000 instrument using NovaSeq S2 v1 chemistry (Illumina) in XP mode for the first batch of 3,000 samples and NovaSeq S4 v1.5 chemistry for the last batch of 480 samples. Paired-end sequencing with 2 × 50 cycles was conducted before demultiplexing using bcl2fastq2 v2.20. Quality control of the sequencing data was performed through FastQC (v0.11.9).

#### RTE subfamily and loci profiling

Expression levels of RTEs in the RNA-seq data were quantified using the Software for Quantifying Interspersed Repeat Elements (SQuIRE) pipeline^75^. The sequencing reads were aligned to the hg38 human reference genome obtained from UCSC, and the RTEs were annotated using RepeatMasker. The SQuIRE pipeline generates read counts and fragments per kilobase transcript per million reads (FPKM) for RTE subfamilies. In our analysis, we included RTE subfamilies expressed in at least 5% of the participants with an average of mapped reads equal or greater than 15. FPKM counts were log transformed to conform the data to a normal distribution.

#### Gene expression profiling

We obtained gene expression levels in the RNA-seq data by aligning the sequencing reads to the GRCh38.p13 human reference genome provided by Ensembl using STAR v2.7.1. We obtained the count matrix through the STAR command –quantMode GeneCounts using the GRCh38.101 human gene annotation. Genes expressed in at least 5% of the participants and with an overall mean expression greater than 15 reads were included in the analysis. Last, we applied the varianceStabilizingTransformation function from DESeq2 v1.30.1 on the counts to normalize for library size and to log transform the data.

#### Demographic and biochemical variables

Age, sex and smoking status data were derived from self-reports. Smoking status was categorized as a binary variable (for example, current smoker and not-current smoker). We imputed missing smoking status values using cotinine metabolite levels, measured through the Metabolon HD4 platform^76^. Differential blood cell counts were measured at the Central Laboratory of the University Hospital in Bonn using EDTA-whole-blood samples on a hematological analyzer Sysmex XN9000.

#### Statistical analysis

Before conducting statistical analyses, we *z* standardized all continuous variables to enable comparisons among variables with different units of measurement. To assess the association between *DAXX* (main independent variable) and each RTE subfamily (dependent variable), we performed a multivariable regression analysis, while adjusting for age, sex, smoking status and batch effect (Model 1) and additionally for white and red blood cell counts (Model 2). The FDR method was used to correct for multiple comparisons, with the threshold for statistical significance set at FDR < 0.05. A sensitivity analysis was performed on a subset of the Rhineland Study population. This subset comprised participants with the lowest 20% of *DAXX* expression levels (low*-DAXX* group) and those with the highest 20% of *DAXX* expression levels (high*-DAXX* group). Subsequently, we assessed the association between *DAXX* groups and each RTE subfamily, while controlling for the same covariates as described above.

To interpret the biological role of *DAXX*, we first assessed its correlation with all genes available in the Rhineland Study dataset. Next, we conducted a rank-based gene set enrichment analysis, ranking the genes in descending order according to the Spearman’s correlation of their expression levels with that of *DAXX*. Specifically, a GO analysis for the correlation of *DAXX* expression with the expression levels of 12,167 other genes measured in the Rhineland Study (*N* = 2,966) was performed. All genes were first ranked in descending order according to the Spearman’s correlation of their expression levels with that of *DAXX*, followed by a rank-based gene set enrichment analysis, applying FDR correction to account for multiple comparisons (all *P* values are two-sided and based on a permutation test using a weighted Kolmogorov–Smirnov-like statistic^77^, as implemented in the clusterProfiler R package v. 3.18.1).

### Animals

All experimental procedures were approved by Landesamt für Verbraucherschutz und Ernährung Nordrhein-Westfalen (formerly Landesamt für Natur, Umwelt und Verbraucherschutz Nordrhein-Westfalen) under protocols 81-02.04.2016.A486, 81-02.04.2020.A346 and 81-02.04.2021.A019.

Mice were maintained on a 12-h light/12-h dark cycle with ad libitum access to regular chow food (ssniff Spezialdiäten). The following mice were used in this study. *Daxx*-floxed mice^27^ were generated by Taconic Artemis. The targeting vector contained a neomycin (*NeoR*) gene surrounded by flipase sequences (*FRT*) and a puromycin (*PuroR*) gene surrounded by F3 sites, which were removed by in vivo Flp-mediated recombination. In the resulting strain, exons 2–7 of *Daxx* were flanked by *loxP* sequences. C57BL/6N floxed *Hira* mice (*Hira*^fl/fl^) were provided by P. Adams (Sanford Burnham Prebys Medical Discovery Institute)^78^. The brain sections of *Rosa26*-Cre^ERT2^; *H3.3A*^fl/fl^*H3.3B*^−/−^ mice were provided by S. Rafii (Department of Medicine, Weill Cornell Medicine)^79^. *Ifnar*-KO mice (028228), *Cx3cr1-*Cre^ERT2^ mice (021160), *Rosa26*-Cre^ERT2^ mice (008463), *Mx1*-Cre mice (003556) and APP/PS1 mice (034823) were purchased from The Jackson Laboratory. The following genotyping primers for the *Daxx*-floxed allele were used: 5′-AGATCCTGTCTCTCCTGTCTATCC-3′ (*Daxx* forward) and 5′-CACTGGGTAGACTAGACTG-TGGC-3′ (*Daxx* reverse). To check for recombination induced by Cre recombinase, we used the following primers: 5′-GGAGGGAGTCGAAGAGTTGG-3′ (*Daxx* forward), 5′-TGCGTTTCCTGTCTTTCGG-3′ (*Daxx* reverse) and 5′-GCTCACGCCTTTAGTCCGAA-3′ (*Daxx* recombined). To induce Cre activation, *Rosa26*-Cre^ERT2^- or *Cx3cr1*-Cre^ERT2^-positive mice were treated with 80 mg per kg (body weight) per day TAM (T5648, Sigma-Aldrich) via oral gavage for five consecutive days. Both sexes were tested and used in most experiments. Behavioral analyses were performed on 4- to 5-month-old male mice. The *n* values in the figure legends reflect the number of mice analyzed in each experiment.

### Behavioral tests

#### Open field

To assess their exploratory and locomotor behavior, mice were placed in a lighted 25-cm-wide box with no escape for a single 10-min session. The box itself was divided into a central zone and a peripheral zone. The distance and/or the movement within the zones was recorded to calculate the fear versus exploratory behavior^80^.

#### Elevated plus maze

To investigate anxiety- and fear-associated behavior^81^, the animals were placed for 5 min in the center of a cross-shaped maze composed of two open arms (no walls) and two closed arms (with walls). Their anxiety was triggered by the height of the 1-m-high platform, prompting the animals to seek protection in the closed arms. As a measure of this anxiety, the time spent in the closed arms was recorded.

#### Rotarod

To assess their motor coordination and balance, the mice were placed on an accelerating rotarod for 5 min, which increased in speed from 4 to 40 rpm over time. The test was repeated four times per day for 4 consecutive days. The duration of their stay on the rod was recorded^82^.

#### Barnes maze

Spatial learning and memory were tested using the Barnes maze test^83^. Briefly, animals were trained to locate a 1-m-high circular platform with 20 equally sized holes along the perimeter. An escape platform (circle, 10 cm in diameter) was placed 1.0 cm below the water surface (hidden) in a pool 120 cm in diameter. Each mouse was placed in the center of the maze covered by a dark start box for 10 s. After 10 s, the mouse was allowed to freely explore the platform for a limited time period (90 to 180 s). The test consisted of three different phases: (1) habituation (the first day with one trial per mouse), (2) acquisition (5 consecutive days with two trials per mouse) and (3) probe test (on days 7 and 14 with one trial per mouse). During the habituation phase, the mouse could explore the platform for only 1 min and was then led to the escape hole, where it remained in the escape box for another 2 min. However, during the acquisition phase, the mouse had 3 min per trial to explore the maze and find the escape hole/box. In case of failure, the mouse was led again to the escape hole, where it remained for an additional 2 min. Last, the final probe tests were performed 24 h and 7 days after the last acquisition. During the probe tests, the escape hole was plugged, and the mouse was allowed to explore the maze for a total time period of 120 s. The latency to reach the plugged escape hole and the cumulative duration spent in the target area were measured for the 2 probe days.

### Primary cell culture

For primary microglia culture, cortical hemispheres from newborn mice (P2) were cleared of meninges and homogenized in ice-cold DMEM supplemented with 20% FBS and 1% penicillin/streptomycin. After filtration through a 70-µm cell strainer, the cell suspensions were centrifuged at 700*g* for 5 min, followed by resuspension. The cells were then plated in a poly-L-lysine-coated T25 flask and maintained in a 37 °C incubator with 5% CO_2_. After 24 h, half of the culture medium was replaced with fresh medium supplemented with 10% L929 supernatant. The medium was changed every second day for 7–10 days, until the coculture of microglia and astrocytes had reached the desired confluence. The cultured microglia were detached from the astrocyte layer by ‘shaking’ the flask and were replated for further experiments.

For primary BMDM cultures, the hind legs of 3-month-old mice were collected. After separating the tibiae and femurs, the bone marrow was flushed through a 70-µm cell strainer. In total, 1.4 × 10^6^ cells were plated per 150-mm nontreated dish into DMEM (11960, Gibco) supplemented with 10% FBS, 1% penicillin/streptomycin, 1× GlutaMAX (35050061, Gibco) and 20% freshly added L929 supernatant. After 4 days in cell culture, half of the medium was replaced with fresh 10% supplemented medium. At day 6, the cells were detached using cell dissociation buffer (13151-014, Gibco), counted and replated for further experiments.

To induce *Daxx* deletion in *Cx3cr1*-Cre^ERT2^; *Daxx*^fl/fl^ microglia or *Rosa26*-Cre^ERT2^; *Daxx*^fl/fl^ BMDMs, the cultured cells were treated with 5 µM 4-hydroxytamoxifen (SML1666-1ML, Sigma-Aldrich) for 24 h. The culture medium was then replaced with fresh medium, and the cells stayed in culture for a total period of 5 days to obtain a completed *Daxx* deletion.

NPCs were isolated from E13.5 mouse cortex. Cells were cultured in Neurobasal medium containing 10% supplement (proliferation), 1% penicillin/streptomycin, 10 ng ml^−1^ epidermal growth factor and 10 ng ml^−1^ fibroblast growth factor in a 37 °C incubator with 5% CO_2_. To overexpress *Daxx* in NPCs, 150,000 cells per well were seeded in six-well plates with 2 ml of DMEM and incubated overnight at 37 °C. On the second day, the medium was replaced with fresh medium 1 h before transduction. The volume of the virus per well was calculated at a multiplicity of infection of 7 (volume = (multiplicity of infection × number of cells / total). Empty vector (pHIV-dTomato) and Flag-DAXX (Flag-DAXX-pHIV-dTomato) lentiviruses were mixed into the medium and added to the cells for lentiviral transfection, followed by an 8-h incubation at 37 °C. After 8 h, the medium was replaced with fresh medium, and the cells were further incubated for another 48 h. Transduced NPCs were then prepared for fluorescence-activated cell sorting in a concentration of 10 × 10^6^ cells per ml. The tdTomato^+^ cells were sorted and plated in 24-well plates and T25 flasks for further expansion. The expanded cells were subsequently used for immunohistology and immunoblotting assays.

### ELISA

For the quantification of secreted proinflammatory cytokines (IFNβ and TNF) in BMDM supernatants, ELISAs were performed using Mouse IFN-β DuoSet (DY8234, R&D Systems) and Mouse TNF ELISA Set II (558534, BD OptEIA) ELISA kits, according to the manufacturers’ guidelines.

### Microglia isolation from adult mouse brains

Microglia were isolated from adult mouse brains using the Adult Brain Dissociation kit from Miltenyi Biotech according to the manufacturer’s instructions. In brief, after euthanasia, mouse brains were mechanically and enzymatically digested at 4 °C. Fully digested tissue suspensions were filtered with a 70-µm cell strainer, followed by subsequent removal of myelin, cell debris and erythrocytes. The cell suspension was then incubated with CD11b MicroBeads (Miltenyi Biotech) for 15 min at 4 °C, followed by a separation in MS columns (Miltenyi Biotech) using a magnetic field on a MACS MultiStand and QuadroMACS (Miltenyi Biotech). The isolated microglia were collected for further experiments, such as protein extraction or RNA isolation.

### Immunohistochemistry and image analysis

Brains were collected from mice and fixed overnight in 4% paraformaldehyde in 0.1 M PBS. The fixed brains were placed in a mixture of 20% glycerol and 2% dimethyl sulfoxide in 0.4 M PBS for 24 h for cryoprotection. Brain sections (40 µm) were collected in 0.1 M PBS. Primary mouse or rat monoclonal, rabbit, guinea pig or chicken polyclonal antibodies to ApoE (ab183597, Abcam), bassoon (ADI-VAM-PS003-F, Enzo), CD68 (137001, Biolegend), cleaved caspase-3 (9661, Cell Signaling), DAXX (sc-7152, Santa Cruz), GFAP (ab4674, Abcam), γH2AX (9718, Cell Signaling), Homer1 (160006, Synaptic Systems), IBA1 (234004, Synaptic Systems), Ki67 (ab16667, Abcam), LAMP1 (ab25245, Abcam), MBP (ab7349, Abcam), NeuN (ab134014, Abcam), SMI31 (801602, Biolegend) and SOX9 (ab185230, Abcam) were used. Sections were washed three times in PBS (3 × 15 min each), followed by washing several times in PBS containing 0.3% Triton X-100 (9 × 20 min each). Sections were preincubated with PBS containing 5% normal goat serum and 0.3% Triton X-100 for 1 h to block nonspecific binding and subsequently incubated with primary antibody at 4 °C for 48 h. Sections were then washed nine times for 20 min each in 0.3% Triton X-100 in 0.1 M PBS and incubated with Alexa-conjugated secondary antibodies (1:500) for 2 h using standard techniques. Finally, sections were mounted on gelatin-coated glass slides and coverslipped in Immu-Mount. Co-staining of SA-β-Gal activity and IBA1 protein in brain sections was performed by staining for SA-β-Gal first using the Senescence β-Galactosidase Staining kit (9860, Cell Signaling). Subsequently, sections were processed for immunofluorescence staining as described previously. Fluorescence was imaged under a confocal microscope (Zeiss, LSM900). Digital images were analyzed with Fiji/ImageJ.

For microglial morphology analysis, fluorescence emission sections were scanned at a resolution of 1,024 × 1,024 pixels with 8-bit sampling in sequential scanning frame-by-frame mode using a ×20 Plan-Apochromat objective. Analysis of microglial morphology was performed using the ImageJ MotiQ plugin, as previously described^84,85^. For high-resolution three-dimensional analysis, samples were scanned using a Plan-Apochromat ×63/1.32-NA oil DIC objective at a resolution of 1,024 × 1,024 pixels with 8-bit sampling in sequential scanning frame-by-frame mode. Single optical sections were acquired using identical acquisition settings, with a pinhole of 1 Airy unit. Stacks of 20–29 optical sections yielded voxel dimensions between 100 and 400 nm for the *x*, *y* and *z* planes. Three-dimensional reconstructions were generated with Imaris v9.8 software (BitPlane, Oxford Instruments).

### Western blotting

Total protein from brain tissue or microglia was lysed with freshly prepared modified RIPA buffer (50 mM Tris pH 8, 0.5 mM EDTA, 150 mM NaCl, 0.5% SDS, 1% Triton X-100). The lysates were quantitated using a Pierce BCA Protein Assay kit (23225, Thermo Fisher Scientific). Equal amounts of protein (10–30 μg) were separated by SDS–PAGE electrophoresis, transferred to a PVDF membrane and blocked in 2.5% skim milk in Tris-buffered saline with Tween-20 (TBST). Membranes were then incubated overnight with primary antibody solution at 4 °C followed by a 2-h incubation with secondary antibody at room temperature. Proteins were detected using the Odyssey Infrared Imaging System (LI-COR Biosciences). The following primary and secondary antibodies were used in this study: ApoE (ab183597, Abcam), β-actin (A5541, Sigma-Aldrich), CD68 (137001, Biolegend), DAXX (sc-7152, Santa Cruz), DAXX (ab32140, Abcam), GAPDH (ab8245, Abcam), GFAP (ab4674, Abcam), H2AX (2595, Cell Signaling), γH2AX (9718, Cell Signaling), H3.3 (09-898, Millipore), TRIM28 (ab10484, Abcam), MDA5 (PA5-20337, Thermo Fisher), MBP (ab7349, Abcam), p53 (NCL-L-P53-CM5P, Leica Biosystems), p-p53 (9284, Cell Signaling), PML (05-718, Millipore), STAT1 (9172S, Cell Signaling), p-STAT1 (9167S, Cell Signaling), synaptophysin (4329, Cell Signaling) and Alexa Fluor 680- or IRDye 800-labeled goat anti-rabbit, mouse, rat and chicken IgG antibodies. GAPDH or β-actin was used as a loading control. Blots were quantified using ImageJ (National Institutes of Health, https://imagej.nih.gov/ij/).

### RNA extraction and gene expression analysis

Total RNA was extracted from brain-isolated cells or primary cultured cells using the RNeasy Mini kit (Qiagen) according to the manufacturer’s instructions. RNA concentration was quantified using an ND1000 Spectrophotometer (NanoDrop), followed by reverse transcription using the High-Capacity cDNA Reverse Transcription kit (Applied Biosystems). Quantitative real-time PCR was performed on a 7500 Fast Real-Time PCR system (Applied Biosystems) using Fast SYBR Green Master Mix (Applied Biosystems). Relative abundance of the specific transcripts was normalized to *Gapdh* mRNA and calculated using the ${2}^{-\Delta \Delta {C}_{{\rm{t}}}}$ method. The housekeeping gene *Gapdh* was used as an internal control for mRNA expression. The following primer sequences were used: 5′-GATGACTATAGGCCAGGCGT-3′ (*Daxx* forward), 5′-TCGTCTCTTCTGTCTCTCGC-3′ (*Daxx* reverse), 5′-CTCCACTCACGGCAAATTCAA-3′ (*Gapdh* forward), 5′-GATGACAAGCTTCCCATTCTCG-3′ (*Gapdh* reverse), 5′-ACAGATCAGCTCGAGTGGCAAA-3′ (*Apoe* forward), 5′-ATCTTGCGCAGGTGTGTGGAGA-3′ (*Apoe* reverse), 5′-CCTGGTGATGTCCGACCTG-3′ (*Cdkn1a* forward) and 5′-CCATGAGCGCATCGCAATC-3′ (*Cdkn1a* reverse).

### Bulk RNA-seq data processing and analysis

#### Bulk RNA-seq library preparation

For RNA-seq, the extracted RNA was resuspended in RNase-free water, and RNA quantity and quality (RINe) were assessed via the HS RNA analysis ScreenTape assay on a 4200 TapeStation system (Agilent Technologies). Libraries were prepared using the SMARTer Stranded Total RNA-Seq Kit v3 according to the manufacturer’s instructions (Takara Bio). Briefly, total RNA was reverse transcribed into cDNA using N6 random primers, and adapters for Illumina sequencing were added through PCR via a limited-cycle PCR program. The PCR products were purified using AMPure XP beads and further amplified via a limited-cycle PCR program to generate multiplexed sequencing libraries. Library concentration was measured using the Qubit dsDNA HS Assay kit (Thermo Fisher Scientific), and size distribution was assessed using the HS D5000 ScreenTape assay on a 4200 TapeStation system (Agilent Technologies). Libraries were sequenced on an Illumina NextSeq 500 or Illumina NovaSeq 6000 according to the manufacturer’s instructions.

#### RNA-seq analysis

For coding genes, FASTQ files were adaptor trimmed using TrimGalore with default parameters, aligned to genome build mm10 by STAR, filtered by mapq > 10 using SAMtools and deduplicated using Picard (https://broadinstitute.github.io/picard/). A count matrix was assembled using featureCounts. DESeq2 was then used to perform rlog/vst transformation, generate PCA plots, acquire the normalized counts and identify differentially expressed genes.

For repetitive elements, SQuIRE^75^ was used to handle genome alignment, count matrix generation and identify differentially expressed repetitive elements. Subfamily counts from all samples were assembled, rounded to integers and supplied to DESeq2 for normalization. The adjusted *P* values from DESeq2 were used for volcano plots and selection of differentially expressed genes.

#### Clustering of RTE subfamily expression in GSE79819

Normalized subfamily counts were averaged across replicates, log_2_ transformed and split into two tables for LTRs and non-LTRs. Subfamily expression in each table was *z*-score transformed across samples, and subfamilies containing any ‘NA’ values were removed before clustering using the KMeans method from the sklearn Python package.

#### Clustering of RTE subfamily expression in GSE208386

Samples from both sexes were selected. RTE analysis and normalization followed the same procedures as described earlier. Subfamilies were filtered out if their normalized expression had a value of 0 in more than half of the total samples. Normalized subfamily counts were averaged across replicates without distinction of sex. The averaged counts were log_2_ transformed, split into two tables for LTRs and non-LTRs, *z*-score transformed across all ages and clustered by *k*-means = 5 using the sklearn package.

#### Selection of RTE copies that responded to LPS treatment at 6 h then subsided at later time points

Differentially expressed RTE copies in wild-type mice after LPS treatment were obtained using the following comparison settings: (1) LPS 6 h versus untreated (Comp. A), (2) LPS 24 h versus untreated (Comp. B) and (3) LPS 48 h versus untreated (Comp. C). A preselected set of RTE copies was identified as any RTE copy having an adjusted *P* value of <0.05 in at least one comparison among Comp. A, Comp. B or Comp. C. The preselected RTE copies were further filtered by (log_2_ (fold change) Comp. A > 0) AND (log_2_ (fold change) Comp. B < log_2_ (fold change) Comp. A) AND (log_2_ (fold change) Comp. C < log_2_ (fold change) Comp. B) to get the targeted RTE copies.

#### Heat map of RTE expression in LPS-treated ex vivo dataset

Locus-specific RTE counts for LPS-treated ex vivo wild-type and *Daxx*-KO samples were normalized using the same procedure applied for subfamily counts. The normalized transposable element counts for targeted RTE copies were extracted, averaged across replicates, *z*-score transformed, split into two tables for LTRs and non-LTRs and clustered using the KMeans method from the sklearn package. Clusters of RTE copies that did not show reduced expression at 24 h compared to 6 h in *Daxx*-KO mice were extracted, and their loci were supplied to GREAT (http://great.stanford.edu/) for GO analysis using default settings.

#### Analysis of neighboring genes for the top 30 overexpressed intergenic LTRs in ex vivo LPS treatment

Locus-specific differential RTE expression from SQuIRE was filtered to keep intergenic LTRs. Intergenic LTRs were identified by the locus not overlapping with known genic regions (downloaded by Table Browser from the UCSC GenomeBrowser), which included upstream 5-kb and downstream 1-kb extensions. The intergenic locus-specific SQuIRE results in each comparison were first filtered by an adjusted *P* value of <0.05, and the loci in the top 30 log_2 _(fold change) were collected from each comparison. The top 30 loci from each comparison were pooled, and their neighboring genes were obtained by using the annotatePeakInBatch function from the ChIPpeakAnno package with maxgap = 20,000. The log_2_ (fold change) and adjusted *P* values for the neighboring genes in the *Daxx*-KO versus control DESeq2 results were retrieved for PBS and 6-, 24- and 48-h LPS treatment. A heat map was produced by color coding log_2_ (fold change) and printing the adjusted *P* value inside the grid.

#### Clustering of locus-specific RTEs in in vivo samples

Differentially expressed RTEs between *Cx3cr1*-Cre^ERT2^; *Daxx*^WT/WT^ and *Cx3cr1*-Cre^ERT2^; *Daxx*^fl/fl^ microglia at various time points were selected by an adjusted *P* value of <0.1 and pooled. Unique RTEs were identified from the pooled set, and the log_2_ (fold change) values from various time points were assembled for the unique RTEs. The assembled log_2_ (fold change) table was split according to LTRs and non-LTRs. Each table for LTRs/non-LTRs was clustered using a Gaussian mixture model from the sklearn library into five clusters.

#### Statistical analysis of overlaps among peak sets or gene sets

The R package SuperExactTest^86^ was used to calculate the significance of overlapping among multiple gene sets, and the fisher function from bedtools was used for overlapping between two peak sets.

### ATAC-seq data processing and analysis

#### ATAC-seq library preparation

For ATAC-seq, approximately 50,000 to 100,000 isolated microglia were processed immediately for library construction. The cells were spun down at 500*g* for 5 min and washed with cold PBS. Thereafter, cells were resuspended into a mix of nuclease-free water, 1% digitonin and 10% Tween-20, mixed by vortexing and spun down briefly. The tagmentation reaction was performed by adding Nextera’s Tn5 Transposase (TDE1) in the reaction buffer. The transposition reaction mix was incubated for 30 min at 37 °C, followed by DNA purification using a MinElute PCR purification kit (Qiagen). ATAC-seq libraries were generated from the tagmented DNA by PCR amplification using Nextera index primers (Illumina) according to the manufacturer’s instructions. ATAC-seq libraries were then purified using a PCR purification MinElute kit (Qiagen) and quantified using KAPA library quantification kits (Kapa Biosystems) and a D1000 ScreenTape assay on a Tapestation 4200 system (Agilent). Libraries were sequenced on an Illumina NovaSeq 6000 according to the manufacturer’s instructions.

#### ATAC-seq analysis

The FASTQ files were directly submitted to the atacseq pipeline (version 1.2.2) from nf-core (https://github.com/nf-core/atacseq) with arguments ‘–genome GRCm38’, ‘–keep_multi_map’ and ‘–min_reps_consensus 2’.

#### Motif search over DARs and overlap with microglia developmental enhancers

DARs were defined as an adjusted *P* value of <0.05 or <0.1 in some situations, which were later split into increased/decreased accessible regions according to log_2_ (fold change) and annotated using the ‘annotatePeak’ function from the ChIPseeker R package with ‘TxDb = TxDb.Mmusculus.UCSC.mm10. knownGene’ and ‘tssRegion = c(–3000,3000)’. DARs were defined as being intergenic if ChIPseeker annotated it as ‘Distal Intergenic’ and being nonintergenic otherwise. Motif search using findMotifsGenome.pl from HOMER was performed on DARs with feature combinations between (open; close) and (intergenic; nonintergenic; intergenic and nonintergenic combined). The *P* values from HOMER ‘knownResults’ files were used in heat map plotting.

Loci of LTRs in RepeatMasker data were downloaded from Table Browser on the UCSC Genome Browser. LTRs that overlapped with intergenic DARs were supplied to ‘findMotifsGenome.pl’ from HOMER for motif discovery. LTR loci overlapping with intergenic open regions were supplied to GREAT for GO analysis with default settings.

To calculate the significance of overlapping between differentially increased/decreased accessibility and microglia developmental enhancers, the differentially increased/decreased accessibility regions (adjusted *P* < 0.05) from *Cx3cr1*-Cre^ERT2^; *Daxx*^fl/fl^ microglia at 3 d.p.i. were used as input to the ‘bedtools fisher’ command together with mouse microglia developmental enhancer regions identified in Matcovitch-Natan et al.^30^.

### CUT&Tag data processing and analysis

#### CUT&Tag library preparation

CUT&Tag for H3K9me3 was performed according to previously described protocols^87,88^. In brief, around 50,000 nuclei per reaction were extracted from isolated microglia using NE1 buffer and incubated with activated Concanavalin A beads. Beads were then incubated with anti-H3K9me3 (Active Motif 39062, 1:50 dilution) at 4 °C overnight, followed by incubation with secondary antibody for 60 min. The transposase reaction was performed with pA(G)-Tn5 (1:20 dilution) in 300-wash buffer for 60 min at room temperature. Tagmentation was performed for 1 h in 300-wash buffer supplemented with 10 mM MgCl_2_ in a 50-µl volume. After tagmentation, the beads were washed in 50 µl of TAPS buffer and resuspended in 5 µl of SDS release buffer for 60 min at 58 °C. Transposed DNA fragments were amplified using NEBNext High-Fidelity PCR Master Mix (New England Biolabs) and dual-indexed primers from IDT for Illumina Nextera DNA UD Indexes Set A (Illumina). Gap filling and 14 cycles of PCR were performed, followed by clean-up with 65 µl of SPRIselect beads (Beckman Coulter).

CUT&Tag for H3.3 was performed using an Active Motif CUT&Tag-IT Assay kit (53160, Active Motif). Briefly, 150,000–200,000 isolated microglia per reaction were bound to activated Concanavalin A beads. After successive incubations with H3.3 (Millipore 09-898, 1:20, overnight at 4 °C) and secondary (60 min) antibodies in Dig-wash buffer, the beads were washed and resuspended in pA-Tn5 transposomes (1:100 dilution) in Dig-300 buffer for 60 min at room temperature. The samples were then incubated in Tagmentation buffer for 60 min at 37 °C. After tagmentation, DNA fragments were further extracted and purified using a DNA purification kit. Subsequently, PCR amplification was performed using i7 and i5 primers for 14 cycles, followed by clean-up with 65 µl of SPRIselect beads (Beckman Coulter).

Quality check and measurement of the CUT&Tag libraries were performed using an HS D1000 DNA assay on a 4200 TapeStation system (Agilent Technologies). Libraries were sequenced as paired-end 2 × 75 bp reads on a NovaSeq X Plus system (Illumina). The experiments were run in duplicate or triplicate.

#### Data preprocessing

CUT&Tag reads were aligned using Bowtie2 (v2.2.1)^89^ to the GRCm39/mm39 (or mm10 for the cluster analysis in Fig. 5a,b) reference genome with settings ‘--local --very-sensitive-local --no-unal --no-mixed --no-discordant --phred33 -I 10 -X 700’. BAM files were then created and sorted using SAMtools (v1.11)^90^, before removing PCR duplicates using Picard (v2.20.4; http://broadinstitute.github.io/picard/) subroutine MarkDuplicates. ATAC-seq reads were aligned using BWA-MEM (v0.7.8-r455)^91^ to the GRCm39/mm39 reference genome with default settings. BAM files were then created and sorted using SAMtools (v1.11) before removing PCR duplicates using Picard (v2.20.4) subroutine MarkDuplicates. Merged samples were created using the SAMtools (v1.11) subroutine merge. Heat maps and profiles were plotted using deepTools (v3.5.3). Transposable element coordinates were extracted from the UCSC mm39 RepeatMasker (rmsk) file.

#### Differential binding

Human microglia-specific enhancers and promoters were extracted from Nott et al.^54^. The coordinates were first converted to the mm10 genome and subsequently to the mm39 genome using UCSC-tools liftOver with a minimum 50% sequence identity. Binding changes were assessed using DiffBind (http://bioconductor.org/packages/release/bioc/html/DiffBind.html) with FDR < 0.05. While for microglia-specific enhancers, strict settings were applied (bUseSummarizeOverlaps = TRUE; plotMA_bUsePval = FALSE), for IAP elements, more lenient settings were used (bUseSummarizeOverlaps = FALSE, plotMA_bUsePval = TRUE).

#### H3.3^+^ subclustering

Profile plots of LTR subfamilies were assessed to obtain potential subclusters of H3.3^+^ sites following *Daxx* loss. To deduct the small set of binding sites that exhibit higher H3.3 binding in *Cx3cr1*-Cre^ERT2^; *Daxx*^f^^l/fl^ microglia, we used the *k*-means clustering approach (nondeterministic; no seed) implemented in deepTools (v3.5.3). For the IAP and ‘LTR (rest)’ subfamilies, we initialized with increasing cluster sizes of 4, 6 and then 8 to observe whether the pattern remained/narrowed down candidates far enough. For *k*-means = 8, we obtained the clusters 8 (*n* = 10,821; 36.06%) for IAP and 7 (*n* = 2,293; 15.00%) for ‘LTR (rest)’.

#### Motif search

Enriched de novo motifs were screened with HOMER (v5.1)^92^ using findMotifsGenome.pl with default settings (‘-size 200 -mask’) and the following input peaks: IAP–TSS peaks that were found to be more differentially bound in wild-type microglia than in *Cx3cr1*-Cre^ERT2^; *Daxx*^fl/fl^ microglia (using DiffBind; *P* < 0.05; fold change > 0).

#### LTR proximity analysis

To assess the statistical significance of deregulated LTRs (belonging to ERV1, ERVK, ERVL, ERVL-MaLR, the IAP subgroup or the ‘LTR (rest)’ subgroup) within a specified range (<50 kb) of gene TSSs, we randomly selected 50 unique gene names associated with a TSS (Ensembl GRCm39.v114) using the UNIX function shuf and repeated this 100 times. The resulting mean value of enriched TSSs was used in a contingency table for Fisher tests. For enrichment analysis, we compared it to a preselected ‘Cell cycle’ gene list for strongly deregulated LTRs within 50 kb. We also used the human cell cycle KEGG pathway hsa04110 (accessed 2 October 2025; *n* = 158 genes, 154 mouse homologs) using the same approach.

### scRNA-seq data processing and analysis

#### BD Rhapsody pipeline to prepare scRNA-seq libraries

Isolated microglia were labeled with unique oligonucleotide sample barcodes conjugated to antibodies to mouse CD45 (BD Mouse Immune Single-Cell Multiplexing kit, 633793), pooled in equivalent amounts and loaded on a BD Rhapsody cartridge (BD Bioscience, 400000847) using a BD Rhapsody Express Single-Cell Analysis System. Cell counting was performed using a Countess 3 FL automated cell counter (Invitrogen). Pooled samples were diluted using BD Sample Buffer to a final volume of 650 µl. Approximately 60,000 cells in total were loaded on a BD Rhapsody cartridge. Cell and bead loading, cell lysis, recovery of mRNA-bound beads, reverse transcription and exonuclease treatment were performed according to the manufacturer’s instructions (BD Biosciences). Whole-transcriptome library preparation, based on the random-priming strategy, and index PCR steps were performed using the BD Rhapsody mRNA Whole Transcriptome Analysis and Sample Tag Library Preparation Protocol, as per the manufacturer’s instructions (BD Biosciences), optimized based on ref. ^93^. Quantification of the final libraries was performed using a Qubit Fluorometer with the Qubit dsDNA HS kit (Thermo Fisher Scientific), whereas the size distribution was assessed using the Agilent High-Sensitivity D5000 assay on a TapeStation 4200 system (Agilent Technologies). Paired-end sequencing (2 × 75 bp reads) was performed on a NovaSeq 6000 System (Illumina) using NovaSeq S1 Reagent Kit (200 Cycles) chemistry.

#### Preprocessing, quality assessment and control of single-cell transcriptome data

Raw bcl files were demultiplexed using Bcl2fastq2 V2.20 from Illumina, and the resulting paired-end scRNA-seq reads were filtered after quality control and matching cell barcodes with the barcode whitelist provided by BD. Subsequently, adaptors were trimmed using Cutadapt 1.16, and reads with a PHRED score of ≥20 were retained^94^. Filtered reads were aligned using STAR 2.6.1b^95^ to a customized genome that contained both the reference genome (mm10, Gencode: GRCm38.p6) and multiplexing tag sequences for aligning the transcriptome read and the sample barcode (hashtag oligonucleotide (HTO)). Quantification of gene expression and unique molecular identifier (UMI) count data matrix generation were performed using the Drop-seq-tools 2.0.0 pipeline (https://github.com/broadinstitute/Drop-seq/). Cells were assigned to the sample of origin by following the HTO-demultiplexing workflow of Seurat4 (refs. ^96,97^).

#### Normalization, cell clustering and cell filtering

In contrast to the Chromium 10x system, which uses water-in-oil droplets, the BD Rhapsody system applied here uses microfluidic chips consisting of microwells to create a plate array where cells can be captured and lysed in the presence of beads coated in oligonucleotides containing a poly(T) region, cell-specific barcodes, UMIs and unique sequence regions that facilitate molecular amplification. This results in lower doublet rates at the same cell capture number^98^.

Our analysis implemented robust doublet correction through a dual approach. First, the BD Rhapsody system includes imaging quality control during the capture process, which directly identifies potential doublets at the hardware level. Second, we used molecular-based doublet detection using sample-specific HTOs. Specifically, we used the HTODemux function with a stringent positive.quantile threshold of 0.9999 to classify cells as singlets, doublets or negatives based on HTO enrichment patterns. This approach resulted in the identification and removal of doublets from our dataset, leaving only high-confidence singlets for downstream analysis. Additionally, standard quality control metrics were applied, removing cells with fewer than 250 genes or more than 500 UMIs per cell and cells with high mitochondrial content, further ensuring the exclusion of low-quality cells and potential technical artifacts. This comprehensive approach to doublet identification is more direct and reliable than computational inference methods used in non-tagged single-cell systems.

scRNA-seq data were analyzed in R (4.1.1) using Seurat v4 (ref. ^96^). The scRNA-seq data matrix was normalized using the NormalizeData function, followed by identification of the top 2,000 most variable features (genes) using the vst method in the FindVariableFeatures function. All genes were scaled and centered using the ScaleData function. PCA was performed on this centered expression matrix. The top 15 principal components (PC) were used to build a shared nearest neighbor cell graph, which was then clustered using the Louvain algorithm (resolution = 0.8) implemented in the FindClusters function. The PCs were then mapped onto two dimensions using the UMAP algorithm. Marker genes from Gerrits et al. were used to manually assign cell-type annotations to each cluster^99^. Clusters not assigned as microglia or macrophage, including a mixed cluster, were removed from further analysis. In addition, cells in clusters with a ‘microglia’ annotation were removed if they expressed high levels of *Ccr2* and *Ly6c2*. Finally, the DecontX function of the Bioconductor package celda (1.10.0) was used to estimate and remove contamination with ambient RNA. The resulting decontaminated count matrix was used to rerun normalization, feature selection, PCA, UMAP and clustering as described above.

Gene signatures for homeostatic, DAM-like, IFN, MHC and proliferating microglia were obtained from Chen and Colonna^100^. Additional gene signatures for microglia subtypes were obtained from Hammond et al.^45^. Gene signature enrichment scores were calculated and added to the Seurat v4 object using the AddModuleScore function.

Trajectories of microglia cells were reconstructed separately for wild-type and DAXX-deficient samples using monocle3 (1.0.0) and its learn graph function with use partition = F. Ordering of cells along pseudotime was done in a semisupervised manner by rooting the trajectory in the cluster where the majority of wild-type cells from the 3-d.p.i. time point fell (C0) or the majority of the DAXX-deficient cells from the 3-d.p.i. time point (C5).

#### Aging/repopulation data sets

The processed scRNA-seq data from Li et al.^33^ and Zhan et al.^46^ were downloaded from GEO (GSE207932 and GSE150169) and respectively merged using Seurat v5.1.0 (ref. ^101^) in R-4.2.1. Cells were slightly filtered for outliers keeping only those with nFeature_RNA > 500, nCount_RNA < 25,000, percent.mt < 20, nFeature_RNA > 500, nCount_RNA < 20,000 and percent.mt < 10, respectively. Data were then log normalized (scale.factor = 10,000) and scaled, and a PCA was conducted. For clustering, only the first 15 PCA dimensions (according to the Elbow plot) were used. Age-associated markers were calculated using the FindMarkers function with default settings and with the F24 versus F03 (one sample with LPS and PBS each) comparison. Repopulation markers were calculated in same manner in comparisons as indicated in Fig. 7e,f.

### Statistical analysis

All statistical analyses were performed using Prism 10 software (GraphPad Software). The numbers of experimental replicates and statistical tests used are indicated in the figure legends. The datasets met the assumptions of the statistical tests used. Sample allocation was random, and mice for experiments were randomly chosen based on availability of the right genotypes. All results are presented as mean ± s.e.m. A *P* value of less than or equal to 0.05 was considered statistically significant. Statistical significance is denoted as follows: *P < 0.05, **P < 0.01 and ***P < 0.001.

During animal studies, mice were labeled by animal identification number, and the experimenter was blinded to genotype during testing. For in vitro studies, genotypes were known to the experimenter due to the limited number of biological replicates. All other analyses were conducted with investigators blinded to the experimental condition.

Sample sizes for behavioral experiments were determined based on the current standard, as the minimum number of mice required to detect significance with an *α* rate set at 0.05 in a standard powered experiment. No statistical methods were used to predetermine sample sizes for other samples, but our sample sizes are similar to those reported in previous publication^27^. No animals and data points were excluded from the analysis. Data distribution was assumed to be normal, but this was not formally tested.

### Reporting summary

Further information on research design is available in the Nature Portfolio Reporting Summary linked to this article.

## Online content

Any methods, additional references, Nature Portfolio reporting summaries, source data, extended data, supplementary information, acknowledgements, peer review information; details of author contributions and competing interests; and statements of data and code availability are available at https://doi.org/10.1038/s41593-026-02404-y.

## Supplementary information

Supplementary Information Supplementary Figs. 1–9.

Reporting Summary

Supplementary Data 1 Statistical source data underlying Supplementary Figs. 1–8.

## Source data

Source Data Fig. 1 Statistical source data.

Source Data Fig. 2 Statistical source data.

Source Data Fig. 3 Statistical source data.

Source Data Fig. 4 Statistical source data.

Source Data Fig. 5 Statistical source data.

Source Data Fig. 6 Statistical source data.

Source Data Fig. 7 Statistical source data.

Source Data Fig. 8 Statistical source data.

Source Data Extended Data Fig. 1 Statistical source data.

Source Data Extended Data Fig. 2 Statistical source data.

Source Data Extended Data Fig. 3 Statistical source data.

Source Data Extended Data Fig. 4 Statistical source data.

Source Data Extended Data Fig. 5 Statistical source data.

Source Data Extended Data Fig. 6 Statistical source data.

Source Data Extended Data Fig. 7 Statistical source data.

Source Data Extended Data Fig. 8 Statistical source data.

Source Data Extended Data Fig. 9 Statistical source data.

Source Data Extended Data Fig. 10 Statistical source data.

Source Data Figs. 1, 3 and 8 and Extended Data Figs. 1–3, 6, 7 and 10 Unprocessed western blots and gels.

## Data Availability

Previously reported data were downloaded from Gene Expression Omnibus and online public resources. Hammond et al.^45^ (GSE121654), Li et al.^33^ (GSE298386, GSE207932), Matcovitch-Natan^30^ (GSE79819), Zhang et al.^34^ (GSE52564), Buttgereit et al.^51^ (E-MTAB-5077), Fixsen et al.^50^ (GSE226092), Gerber et al.^27^ (GSE119309) and Zhan et al.^46^ (GSE150169). Data generated by this study are accessible at GSE255329, GSE267028, GSE276607, GSE272828 and GSE309782. The Rhineland Study’s dataset is not publicly available because of data protection regulations. Access to data can be provided to scientists in accordance with the Rhineland Study’s Data Use and Access Policy. Requests for further information or to access the Rhineland Study’s dataset should be directed to rs-duac@dzne.de. Source data are provided with this paper.

## Code availability

No custom software code was used in the data analysis for this paper.
