# Supplementary material for: Microglia activation by derepression of endogenous retroviruses drives inflammation and cellular senescence
Source: Nat Neurosci. 2026 Aug 17;29(10):2480–98. doi: 10.1038/s41593-026-02404-y (PMC13633122; doi:10.1038/s41593-026-02404-y)
Supplement: Supplementary file 1 [file 41593_2026_2404_MOESM1_ESM.pdf]

nature neuroscience

Supplementary information

https://doi.org/10.1038/s41593-026-02404-y

Microglia activation by derepression of endogenous retroviruses drives inflammation and cellular senescence

In the format provided by the authors and unedited

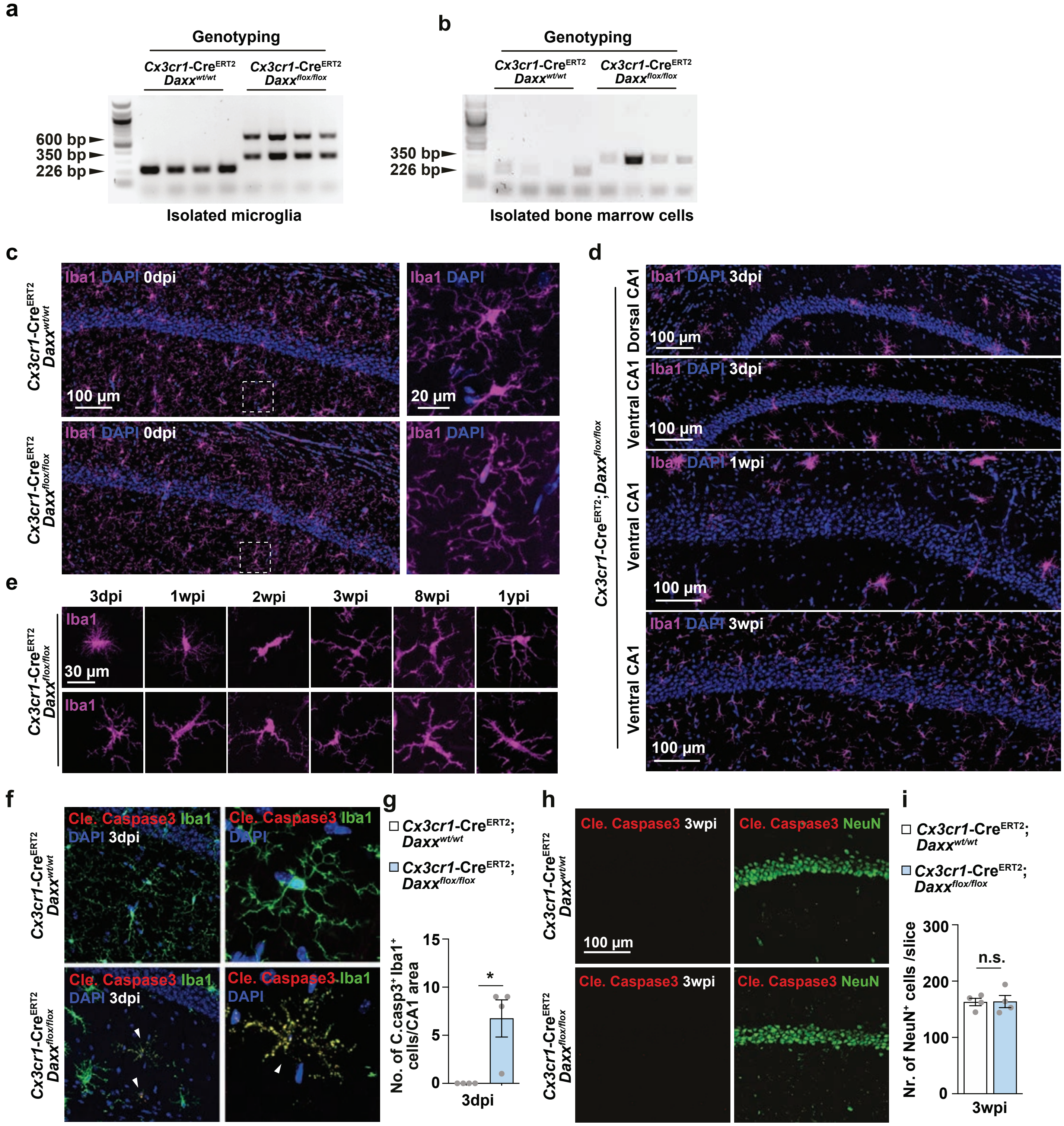

Supplementary Fig. 1. Deletion of *Daxx* leads to morphological changes and induced apoptosis of microglia. **a**, Genotyping of microglia isolated from *Cx3cr1*-Cre^{ERT2};*Daxx*$^{flox/flox}$ and *Cx3cr1*-Cre^{ERT2};*Daxx*$^{wt/wt}$ mice at 3wpi (*n* = 4 mice for each genotype). Arrows indicate the wild-type (226 bp), floxed (350 bp) and recombined alleles (600 bp). **b**, Genotyping of bone marrow cells isolated from *Cx3cr1*-Cre^{ERT2};*Daxx*$^{flox/flox}$ and *Cx3cr1*-Cre^{ERT2};*Daxx*$^{wt/wt}$ mice at 3wpi (*n* = 4 mice for each genotype). Arrows indicate the wild-type (226 bp) and floxed (350 bp). **c**, Representative confocal images of Iba1 immunostaining in the hippocampus of *Cx3cr1*-Cre^{ERT2};*Daxx*$^{flox/flox}$ and *Cx3cr1*-Cre^{ERT2};*Daxx*$^{wt/wt}$ mice at 0dpi (*n* = 3 mice for each genotype). Dashed boxes in the left panel identify high-magnification images of Iba1^{+} microglia (right panel). **d**, Representative confocal images showing Iba1^{+} microglial morphologies in hippocampus of *Cx3cr1*-Cre^{ERT2};*Daxx*$^{flox/flox}$ mice across different time points (*n* = 3 mice per group). **e**, Representative confocal images showing Iba1 immunostaining in the dorsal and ventral CA1 region of the hippocampus of *Cx3cr1*-Cre^{ERT2};*Daxx*$^{flox/flox}$ mice at 3dpi, 1wpi and 3wpi (*n* = 3 mice per group). **f**, **g**, Representative confocal images and quantification of double immunostaining using Iba1 and Cleaved Caspase3 antibodies in the hippocampus of *Cx3cr1*-Cre^{ERT2};*Daxx*$^{flox/flox}$ and *Cx3cr1*-Cre^{ERT2};*Daxx*$^{wt/wt}$ mice at 3dpi (*n* = 4 mice per group, comparison by two-tailed unpaired Student's t test, *P* = 0.0129). **h**, **i**, Representative confocal images and quantification of double immunostaining using NeuN and Cleaved Caspase3 antibodies in the hippocampus of *Cx3cr1*-Cre^{ERT2};*Daxx*$^{flox/flox}$ and *Cx3cr1*-Cre^{ERT2};*Daxx*$^{wt/wt}$ mice at 3wpi (*n* = 4 mice per group, comparison by two-tailed unpaired Student's t test, *P* = 0.9549). Data are presented as mean ± s.e.m. Asterisks (*) indicate statistical significance: *$P < 0.05$, **$P < 0.01$ and ***$P < 0.001$; n.s. indicates not significant.

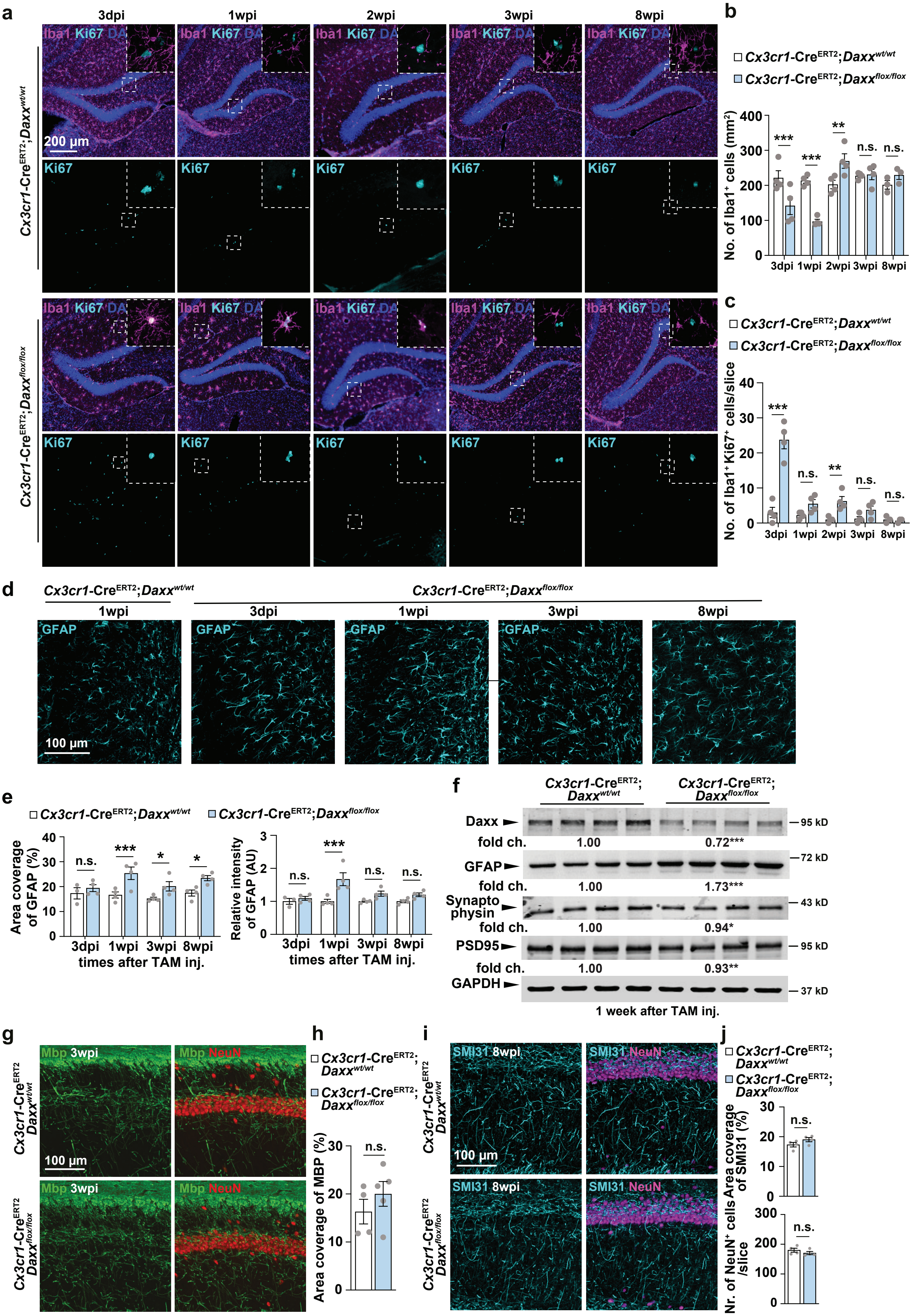
a
3dpi
1wpi
2wpi
3wpi
8wpi
Iba1 Ki67 DA
Ki67
200 μm
Cx3cr1-CreERT2;Daxxwt/wt
Cx3cr1-CreERT2;Daxxflox/flox
b
No. of Iba1+ cells (mm²)
c
No. of Iba1+Ki67+ cells/slice
n.s.
d
GFAP
100 μm
e
Area coverage of GFAP (%)
Relative intensity of GFAP (AU)
times after TAM inj.
f
Daxx
fold ch.
1.00
0.72***
GFAP
1.73***
Synapto physin
0.94*
PSD95
0.93**
GAPDH
95 kD
72 kD
43 kD
37 kD
1 week after TAM inj.
g
Mbp 3wpi
Mbp NeuN
h
Area coverage of MBP (%)
i
SMI31 8wpi
SMI31 NeuN
j
Area coverage of SMI31 (%)
Nr. of NeuN+ cells /slice

Supplementary Fig. 2. *Daxx* deletion in microglia induces microglial proliferation and astrogliosis in mouse brain. **a-c**, Representative confocal images and quantification of double immunostaining using Iba1 and Ki67 antibodies in the hippocampus from *Cx3cr1*-Cre^{ERT2};*Daxx*$^{flox/flox}$ and *Cx3cr1*-Cre^{ERT2};*Daxx*$^{wt/wt}$ mice across different time points (n = 4 mice per group for 3dpi to 3wpi, n = 3 mice per group for 8wpi; for No. of Iba1$^+$ cells, comparison by ordinary two-way ANOVA, F = 12.41, P < 0.0001, Sidak's multiple comparisons test: $P_{3dpi\ Daxx\ WT\ vs\ 3dpi\ Daxx\ KO}$ = 0.0009, $P_{1wpi\ Daxx\ WT\ vs\ 1wpi\ Daxx\ KO}$ < 0.0001, $P_{2wpi\ Daxx\ WT\ vs\ 2wpi\ Daxx\ KO}$ = 0.0041, $P_{3wpi\ Daxx\ WT\ vs\ 3wpi\ Daxx\ KO}$ = 0.87, $P_{8wpi\ Daxx\ WT\ vs\ 8wpi\ Daxx\ KO}$ = 0.2671; for No. of Iba1$^+$Ki67$^+$ cells, comparison by ordinary two-way ANOVA, F = 21.99, P < 0.0001, Sidak's multiple comparisons test: $P_{3dpi\ Daxx\ WT\ vs\ 3dpi\ Daxx\ KO}$ < 0.0001, $P_{1wpi\ Daxx\ WT\ vs\ 1wpi\ Daxx\ KO}$ = 0.0722, $P_{2wpi\ Daxx\ WT\ vs\ 2wpi\ Daxx\ KO}$ = 0.0054, $P_{3wpi\ Daxx\ WT\ vs\ 3wpi\ Daxx\ KO}$ = 0.1617, $P_{8wpi\ Daxx\ WT\ vs\ 8wpi\ Daxx\ KO}$ = 0.8694). Inserted image in the upper-right of each panel showing high-magnification image in white dashed box. **d, e**, Representative confocal images and quantification of GFAP immunostaining in hippocampus of *Cx3cr1*-Cre^{ERT2};*Daxx*$^{wt/wt}$ mice (n = 3 mice for 3dpi and 4 mice for 1wpi, 3wpi and 8wpi) and *Cx3cr1*-Cre^{ERT2};*Daxx*$^{flox/flox}$ mice (n = 4 mice for each age) across different time points (Comparison by ordinary two-way ANOVA with Sidak's multiple comparisons test, for Area coverage: F = 1.41, $P_{Interaction}$ = 0.2562, $P_{3dpi\ Daxx\ WT\ vs\ 3dpi\ Daxx\ KO}$ = 0.3906, $P_{1wpi\ Daxx\ WT\ vs\ 1wpi\ Daxx\ KO}$ = 0.0007, $P_{3wpi\ Daxx\ WT\ vs\ 3wpi\ Daxx\ KO}$ = 0.0357, $P_{8wpi\ Daxx\ WT\ vs\ 8wpi\ Daxx\ KO}$ = 0.0128; for GFAP intensity: F = 21.99, $P_{Interaction}$ = 0.0202, $P_{3dpi\ Daxx\ WT\ vs\ 3dpi\ Daxx\ KO}$ = 0.5089, $P_{1wpi\ Daxx\ WT\ vs\ 1wpi\ Daxx\ KO}$ < 0.0001, $P_{3wpi\ Daxx\ WT\ vs\ 3wpi\ Daxx\ KO}$ = 0.0766, $P_{8wpi\ Daxx\ WT\ vs\ 8wpi\ Daxx\ KO}$ = 0.1271). **f**, Western blots analysis of Daxx, GFAP, Synaptophysin and PSD95 from whole brain lysates of *Cx3cr1*-Cre^{ERT2};*Daxx*$^{flox/flox}$ and *Cx3cr1*-Cre^{ERT2};*Daxx*$^{wt/wt}$ miceat 1wpi (n = 4 mice for each genotype, comparison by two-tailed unpaired Student's t test, P_{Daxx} = 0.0006, P_{GFAP} < 0.0001, $P_{Synaptophysin}$ = 0.0454, P_{PSD95} = 0.0081). GAPDH was used as an internal loading control. **g, h**, Representative confocal images and quantification of myelin basic protein (Mbp) and NeuN immunostaining in hippocampus of *Cx3cr1*-Cre^{ERT2};*Daxx*$^{wt/wt}$ mice(n = 4 mice) and *Cx3cr1*-Cre^{ERT2};*Daxx*$^{flox/flox}$ (n = 5 mice) mice at 3wpi (Comparison by two-tailed unpaired Student's t test, P = 0.3495). **i, j**, Representative confocal images and quantification of SMI31 and NeuN immunostaining in the hippocampus of *Cx3cr1*-Cre^{ERT2};*Daxx*$^{flox/flox}$ and *Cx3cr1*-Cre^{ERT2};*Daxx*$^{wt/wt}$ mice at 8wpi (n = 4 mice per group, comparison by two-tailed unpaired Student's t test, P_{SMI31} = 0.1475, P_{NeuN} = 0.2985). Data are presented as mean ± s.e.m. Asterisks (*) indicate statistical significance: *P < 0.05, **P < 0.01 and ***P < 0.001; n.s. indicates not significant.

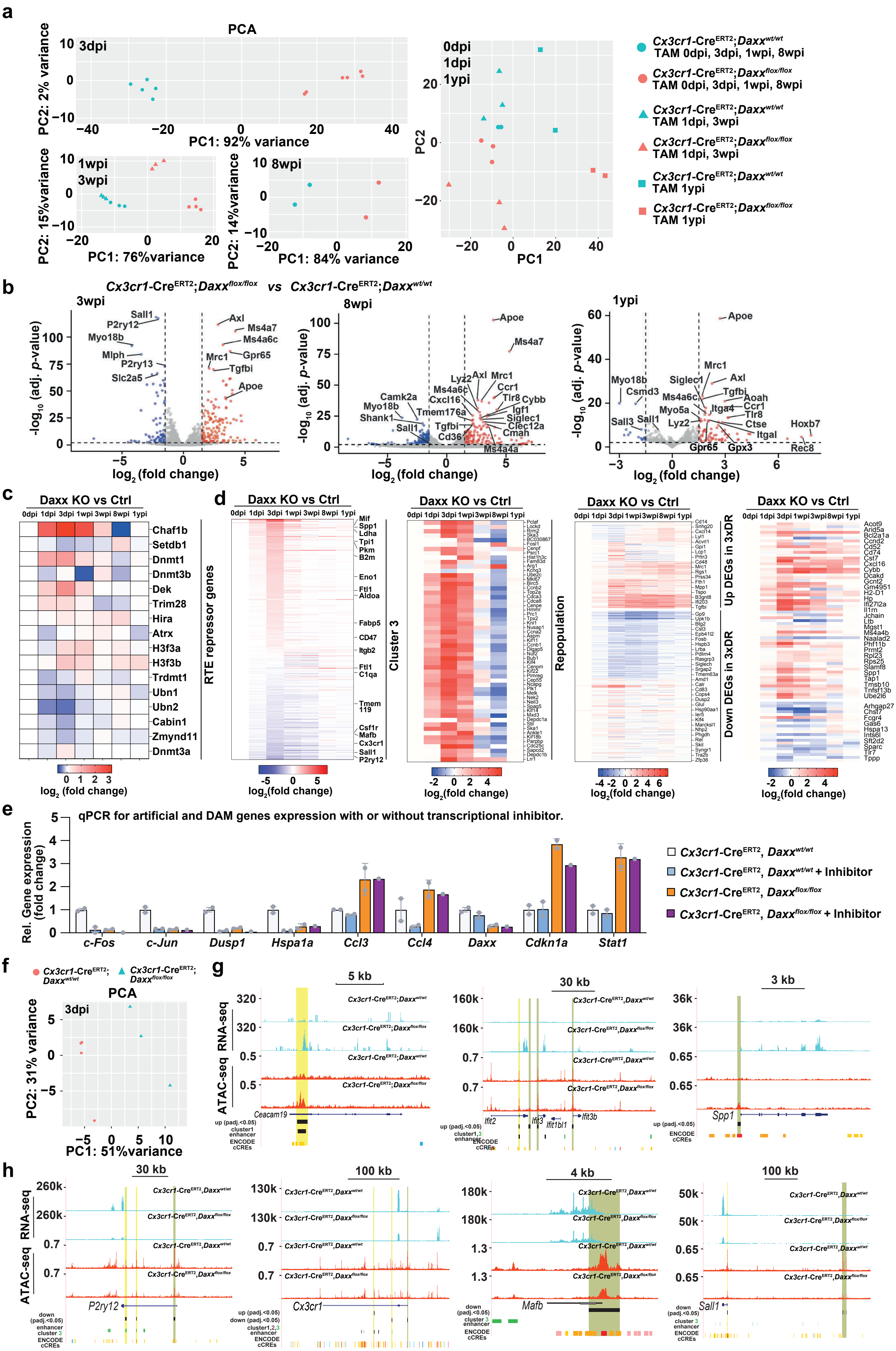

a
PCA
3dpi
PC2: 2% variance
PC1: 92% variance
1wpi
3wpi
PC2: 15%variance
PC1: 76%variance
8wpi
PC2: 14%variance
PC1: 84% variance
0dpi
1dpi
1ypi
PC2
PC1
Cx3cr1-CreERT2;Daxxwt/wt TAM 0dpi, 3dpi, 1wpi, 8wpi
Cx3cr1-CreERT2;Daxxflox/flox TAM 0dpi, 3dpi, 1wpi, 8wpi
Cx3cr1-CreERT2;Daxxwt/wt TAM 1dpi, 3wpi
Cx3cr1-CreERT2;Daxxflox/flox TAM 1dpi, 3wpi
Cx3cr1-CreERT2;Daxxwt/wt TAM 1ypi
Cx3cr1-CreERT2;Daxxflox/flox TAM 1ypi
b
Cx3cr1-CreERT2;Daxxflox/flox vs Cx3cr1-CreERT2;Daxxwt/wt
3wpi
8wpi
1ypi
-log10 (adj. p-value)
log2 (fold change)
c
Daxx KO vs Ctrl
0dpi 1dpi 3dpi 1wpi 3wpi 8wpi 1ypi
Chaf1b
Setdb1
Dnmt1
Dnmt3b
Dek
Trim28
Hira
Atrx
H3f3a
H3f3b
Trdmt1
Ubn1
Ubn2
Cabin1
Zmynd11
Dnmt3a
RTE repressor genes
log2 (fold change)
d
Daxx KO vs Ctrl
Cluster 3
Repopulation
Up DEGs in 3xDR
Down DEGs in 3xDR
P-ADEM
N-ADEM
log2(fold change)
e
qPCR for artificial and DAM genes expression with or without transcriptional inhibitor.
Rel. Gene expression (fold change)
c-Fos
c-Jun
Dusp1
Hspa1a
Ccl3
Ccl4
Daxx
Cdkn1a
Stat1
Cx3cr1-CreERT2, Daxxwt/wt
Cx3cr1-CreERT2, Daxxwt/wt + Inhibitor
Cx3cr1-CreERT2, Daxxflox/flox
Cx3cr1-CreERT2, Daxxflox/flox + Inhibitor
f
Cx3cr1-CreERT2; Daxxwt/wt
Cx3cr1-CreERT2; Daxxflox/flox
PCA
3dpi
PC2: 31% variance
PC1: 51%variance
g
5 kb
30 kb
3 kb
RNA-seq
ATAC-seq
Ceacam19
Ifit2
Ifit3
Ifit1bl1
Ifit3b
Spp1
up (padj.<0.05)
cluster1 enhancer
ENCODE cCREs
h
30 kb
100 kb
4 kb
100 kb
P2ry12
Cx3cr1
Mafb
Sall1
down (padj.<0.05)
ENCODE cCREs

Supplementary Fig. 3. Long-term effects of *Daxx* loss on gene expression and correlation with aging- and repopulation-associated signatures in microglia *in vivo*. **a**, Principal component analysis (PCA) for bulk RNA-seq data of in vivo microglia isolated from *Cx3cr1*-Cre^{ERT2};*Daxx*$^{flox/flox}$ and *Cx3cr1*-Cre^{ERT2};*Daxx*$^{wt/wt}$ mice from 0dpi to 1ypi. **b**, Volcano plot showing differentially expressed genes in microglia isolated from *Cx3cr1*-Cre^{ERT2};*Daxx*$^{flox/flox}$ mice compared to *Cx3cr1*-Cre^{ERT2};*Daxx*$^{wt/wt}$ mice at 3wpi, 8wpi and 1ypi. The significance (adjusted *P*-value) was derived by DESeq2 with a two-sided Wald test and then adjusted for multiple testing using the Benjamini–Hochberg procedure. The adjusted *P*-value and the fold-change are converted to -log10 (adjusted *P*-value) and log2 (fold change), respectively. The vertical and horizontal dotted lines show the cut-off of fold-change = ± 1.5, and of adjusted *P*-value = 0.01, respectively. **c**, Heatmaps showing gene expression change of RTE repressors in microglia isolated from *Cx3cr1*-Cre^{ERT2};*Daxx*$^{flox/flox}$ (*Daxx* KO) compared to *Cx3cr1*-Cre^{ERT2};*Daxx*$^{wt/wt}$ (Ctrl) mice from 0dpi to 1ypi. **d**, Heatmap showing gene expression change for embryonic microglia signature (Clusters 3)[46], microglial repopulation signature[47], aged-like microglial signature with three rounds of depletion–repopulation (3xDR)[33] and age-dependent microglial signature (ADEM)[33] in microglia isolated from *Cx3cr1*-Cre^{ERT2};*Daxx*$^{flox/flox}$ (*Daxx* KO) mice compared to *Cx3cr1*-Cre^{ERT2};*Daxx*$^{wt/wt}$ (Ctrl) mice from 0dpi to 1ypi. **e**, qRT-PCR analysis for the expression of a number of homogenization-induced genes and DAM genes in the microglia isolated from *Cx3cr1*-Cre^{ERT2};*Daxx*$^{flox/flox}$ mice and *Cx3cr1*-Cre^{ERT2};*Daxx*$^{wt/wt}$ mice. During multiple steps of the microglia isolation, cocktail inhibitors were added or not to compare the artificial gene expression (n = 1 mice for *Cx3cr1*-Cre^{ERT2};*Daxx*$^{wt/wt}$ mice with Inhibitor treatment, n = 2 mice for rest groups). **f**, Principal component analysis (PCA) for ATAC-seq data in *in vivo* microglia from *x3cr1*-Cre^{ERT2};*Daxx*$^{flox/flox}$ and *Cx3cr1*-Cre^{ERT2};*Daxx*$^{wt/wt}$ brain at 3dpi. **g, h**, Genome browser visualization of RNA-seq and ATAC-seq signals around *Ceacam19, Ifit2, Ifit3, Ifit3b, Spp1, P2ry12, Cx3cr1, Mafb* and *Sall1* in *x3cr1*-Cre^{ERT2};*Daxx*$^{flox/flox}$ and *Cx3cr1*-Cre^{ERT2};*Daxx*$^{wt/wt}$ microglia at 3dpi. Yellow region highlights the differential ATAC peaks overlapping with different clusters of enhancers in the developing microglia (data from[30]). Green regions highlight the differential ATAC peaks overlapping with candidate cis-Regulatory Elements (cCREs). Data are presented as mean ± s.e.m. Asterisks (*) indicate statistical significance: *P < 0.05, **P < 0.01 and ***P < 0.001; n.s. indicates not significant.

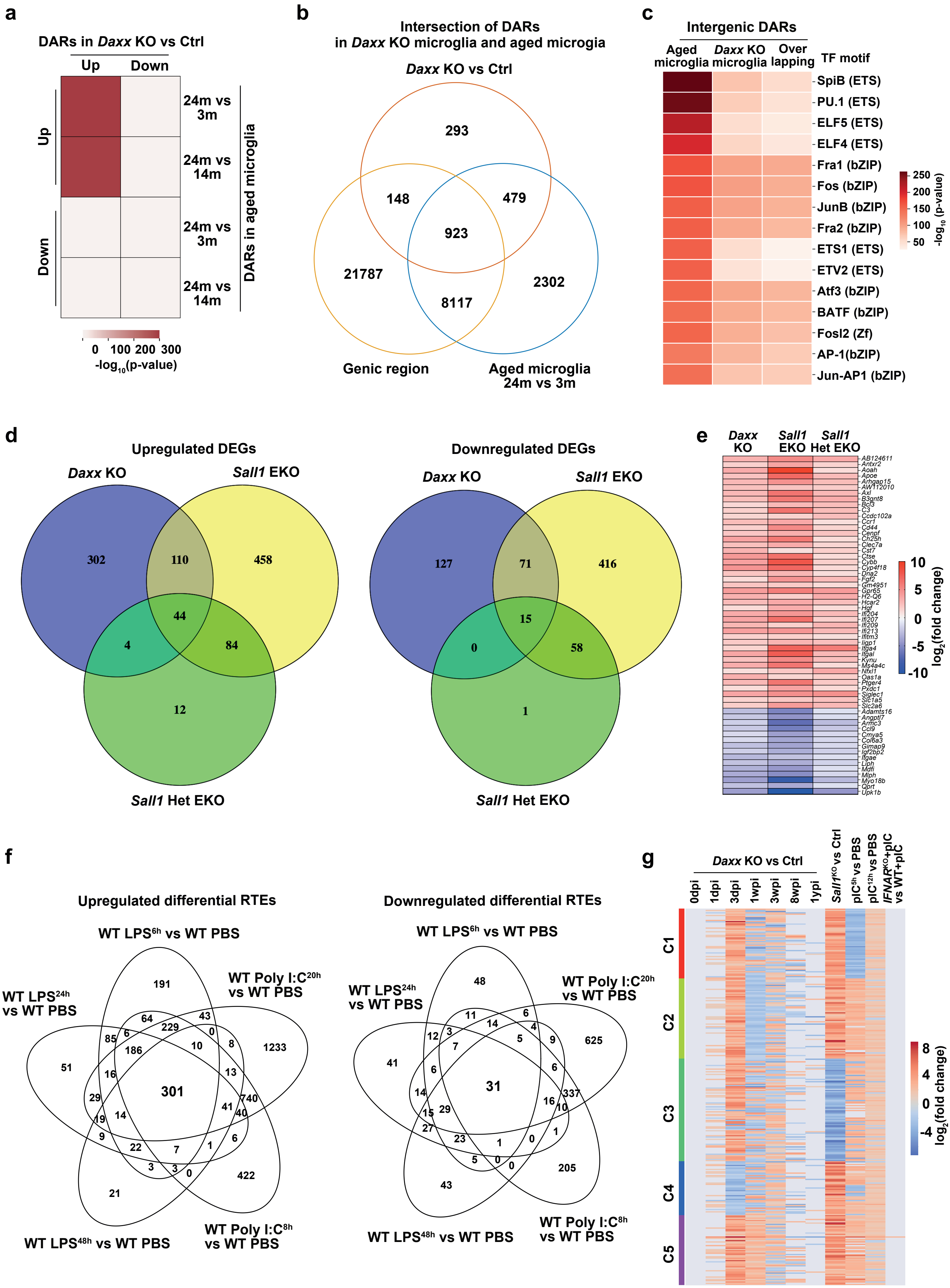

a
DARs in Daxx KO vs Ctrl
Up
Down
24m vs 3m
24m vs 14m
24m vs 3m
24m vs 14m
DARs in aged microglia
0
100
200
300
-log10(p-value)
b
Intersection of DARs in Daxx KO microglia and aged microglia
Daxx KO vs Ctrl
293
148
479
923
21787
2302
8117
Genic region
Aged microglia 24m vs 3m
c
Intergenic DARs
Aged microglia
Daxx KO microglia
Overlapping
TF motif
SpiB (ETS)
PU.1 (ETS)
ELF5 (ETS)
ELF4 (ETS)
Fra1 (bZIP)
Fos (bZIP)
JunB (bZIP)
Fra2 (bZIP)
ETS1 (ETS)
ETV2 (ETS)
Atf3 (bZIP)
BATF (bZIP)
Fosl2 (Zf)
AP-1(bZIP)
Jun-AP1 (bZIP)
250
200
150
100
50
-log10 (p-value)
d
Upregulated DEGs
Daxx KO
Sall1 EKO
302
110
458
44
4
84
12
Sall1 Het EKO
Downregulated DEGs
Daxx KO
Sall1 EKO
127
71
416
15
0
58
1
Sall1 Het EKO
e
Daxx KO
Sall1 EKO
Sall1 Het EKO
AB124611
Antxr2
Aoah
Apoe
Arhgap15
AW112010
Axl
B3gnt8
Bcl3
C3
Ccdc102a
Ccr1
Cd44
Cenpf
Ch25h
Clec7a
Cst7
Ctse
Cybb
Cyp4f18
Dna2
Fgf2
Gm4951
Gpr65
H2-Q6
Hcar2
Hgf
Ifi204
Ifi207
Ifi209
Ifi213
Ifitm3
Iigp1
Itga4
Itgal
Kynu
Ms4a4c
Nfxl1
Oas1a
Ptger4
Pxdc1
Siglec1
Slc1a5
Slc2a6
Adamts16
Angptl7
Armc3
Ccl9
Cmya5
Col6a3
Gimap9
Igf2bp2
Itgae
Liph
Mdfi
Mlph
Myo18b
Qprt
Upk1b
10
5
0
-5
-10
log2(fold change)
f
Upregulated differential RTEs
WT LPS6h vs WT PBS
WT LPS24h vs WT PBS
WT Poly I:C20h vs WT PBS
WT LPS48h vs WT PBS
WT Poly I:C8h vs WT PBS
191
64
43
85
6
229
0
1233
186
10
8
51
16
13
301
29
740
41
19
14
40
9
22
7
1
6
3
3
0
422
21
Downregulated differential RTEs
WT LPS6h vs WT PBS
WT LPS24h vs WT PBS
WT Poly I:C20h vs WT PBS
WT LPS48h vs WT PBS
WT Poly I:C8h vs WT PBS
48
11
6
12
3
14
4
625
7
5
9
41
6
6
31
14
337
16
15
29
10
27
23
1
0
1
5
0
0
205
43
g
Daxx KO vs Ctrl
0dpi
1dpi
3dpi
1wpi
3wpi
8wpi
1ypi
Sall1KO vs Ctrl
pIC6h vs PBS
pIC12h vs PBS
IFNARKO+pIC vs WT+pIC
C1
C2
C3
C4
C5
8
4
0
-4
log2(fold change)

Supplementary Fig. 4. Loss of Daxx affects the chromatin landscape of microglia in vivo and the resulting RTE and gene expression changes are in part recapitulated upon Sall1 loss or viral mimicry. **a**, Heatmap displays the significance of overlapping between differentially accessible regions (DARs) in the *Cx3cr1*-Cre^{ERT2};*Daxx*$^{flox/flox}$ (*Daxx* KO) microglia at 3dpi and DARs in 24-month-old aged microglia (data from[33]); Statistical significance (*P* values) for each pairwise genomic intersection was computed using a two-sided Fisher's exact test via "bedtools fisher" command. **b**, Venn diagrams showing the overlap of open DARs in *Cx3cr1*-Cre^{ERT2};*Daxx*$^{flox/flox}$ (*Daxx* KO) vs *Cx3cr1*-Cre^{ERT2};*Daxx*$^{wt/wt}$ (Ctrl) microglia (24-month-old vs 3-month-old) (data from[33]) as well as genic regions (NCBI RefSeq track on UCSC Genome Browser). **c**, Motif analysis for intergenic open DARs of aging microglia (24-month-old vs. 3-month-old), intergenic open DARs of *Daxx* KO vs Ctrl microglia and the intersecting regions from the two aforementioned regions; Statistical significance (P values) for motif enrichment was determined using a one-sided hypergeometric test against an automatically generated background set of genomic sequences via HOMER software. **d**, Venn diagram showing the overlap of differentially expressed genes (DEGs) in *Daxx* KO microglia at 1wpi, *Sall1* enhance homozygous knockout microglia (*Sall1* EKO) and *Sall1* enhance heterozygous knockout microglia (*Sall1* Het EKO)[51] (*P* = 3.94e-119 for 44 upregulated genes; *P* = 9.06e-119 for 15 upregulated genes; SuperExactTest[88]); Statistical significance (*P* values) for the multi-set intersection among the three independent gene lists was evaluated using a two-sided generalized hypergeometric test implemented via the SuperExactTest R package. **e**, Heatmap showing the expressions of overlap genes in **d** (44 upregulated and 15 downregulated). **f**, Venn diagrams showing the overlap of differentially upregulated and downregulated RTEs in LPS-treated vs PBS-treated wild type microglia (6h), LPS-treated vs PBS-treated wild type microglia (24h), LPS-treated vs PBS-treated wild type microglia (48h), Poly I:C-treated vs PBS-treated wild type microglia (8h), Poly I:C-treated vs PBS-treated wild type microglia (WT, 20h). **g**, Heatmap showing relative expression of 301 upregulated RTEs in microglia isolated from: *Daxx* KO vs Ctrl mice from 0dpi to 1ypi; from *Sall1*-Cre^{ERT2};*Sall1*$^{lox/flox}$ (*Sall1* KO) vs *Sall1*-Cre^{ERT2};*Sall1*$^{wt/wt}$ (Ctrl) mice[52]; from wild type mice treated with the viral mimic Poly I:C (pIC5h, pIC12h) vs PBS-treated; from Poly I:C stimulated IFNAR-KO mice (IFNARKO+pIC) vs Poly I:C stimulated wild type mice (WT+pIC) for 12 hours.

a

IAP TSS peaks with higher H3K9me3 binding in *Daxx* WT vs *Daxx* KO

Motif	P-value	% of Targets	% of Background	Best Match/Details
TACGAGAAAA	1e-7	6.90%	0.25%	PB0032.1_IRC900814_1/Jaspar(0.742)
CGGGACGAGA	1e-6	2.32%	0.00%	ZNF263/MA0528.3/Jaspar(0.672)
GTCAAGGTTT	1e-5	3.45%	0.04%	Prdm15/MA1616.2/Jaspar(0.652)
GGGTTACC	1e-5	10.34%	1.54%	PITX3/MA0714.2/Jaspar(0.730)
AAGAGTTG	1e-3	6.9%	0.85%	ZNF652/MA1657.2/Jaspar(0.775)
TCTTGGCCGC	1e-3	1.15%	0.00%	NFIC/MA0161.3/Jaspar(0.687)
TGGCGTGGCG	1e-3	1.15%	0.00%	CREB3L4/MA1474.2/Jaspar(0.76)
TGTTTGGTCCAG	1e-3	1.15%	0.00%	GLI3(Zf)/Homer(0.645)
GAATTCAT	1e-2	21.84%	10.37%	PB0171.1_Sox18_2/Jaspar(0.775)
GTGCACTGGGTT	1e-2	1.15%	0.00%	PB0091.1_Zbtb3_1/Jaspar(0.632)

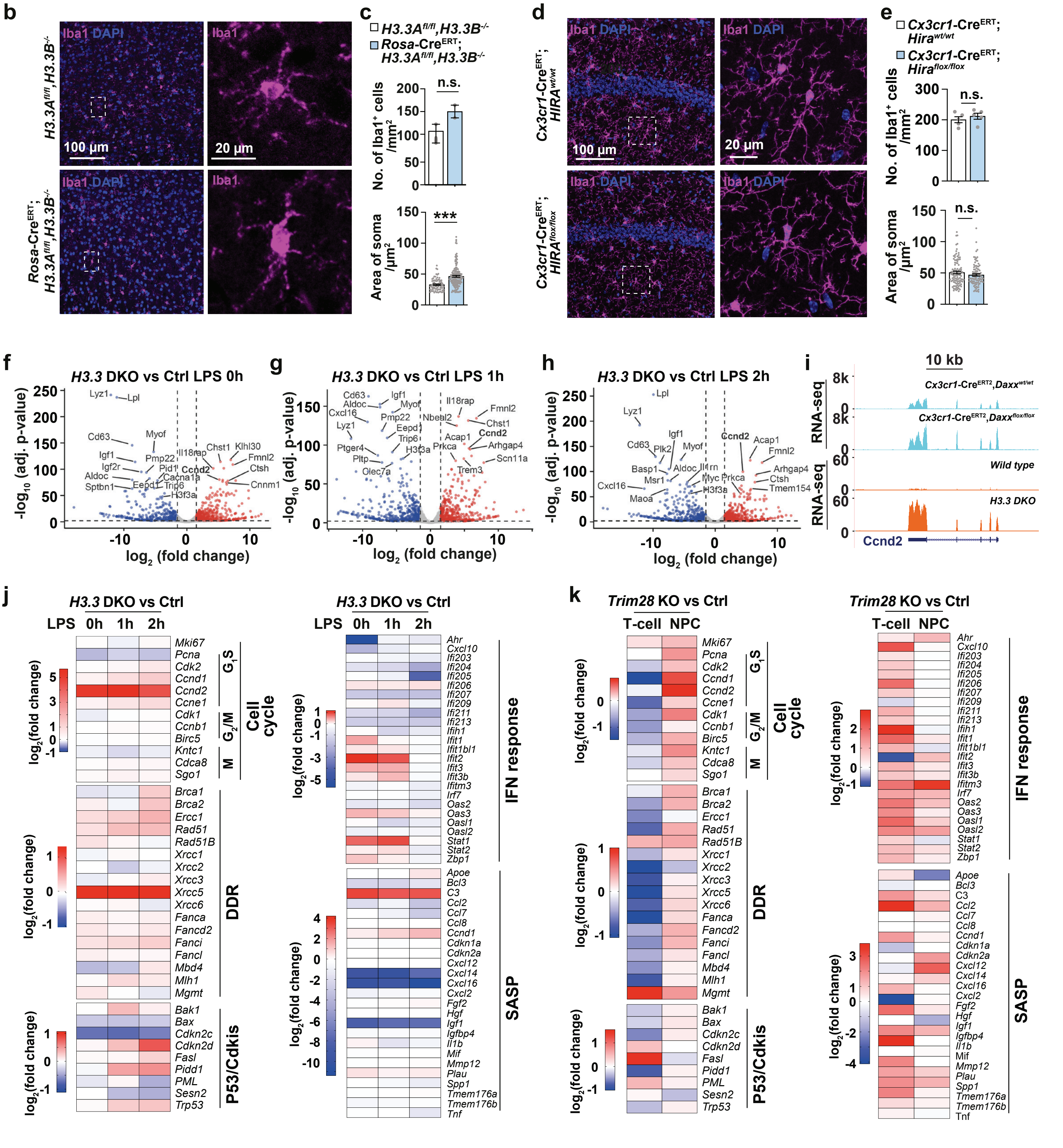

Supplementary Fig. 5. Genetic deletion of *H3.3* genes (*H3f3a/b*) in microglia causes cell hypertrophy and their loss or *Trim28* loss in part recapitulate gene expression changes found in *Daxx*-deficent microglia. a, Top 10 de novo motifs for IAP TSS peaks that were found to be more differentially bound in *Cx3cr1*-Cre^{ERT2};*Daxx*$^{wt/wt}$ (*Daxx* WT) compared to *Cx3cr1*-Cre^{ERT2};*Daxx*$^{flox/flox}$ (*Daxx* KO) microglia (using DiffBind; $P < 0.05$; $fc > 0$). All motifs are marked by software HOMER as "possible false positives". **b, c**, Representative confocal images and quantification of immunostaining using Iba1 in the hippocampus of *H3.3A*$^{flox/flox}$,*H3.3B*$^{-/-}$ (n = 2 mice) and *Rosa*-Cre^{ERT2};*H3.3A*$^{flox/flox}$,*H3.3B*$^{-/-}$ mice (*n* = 2 mice) (Comparison by two-tailed unpaired Student's t test, for No. of Iba1^{+} cells $P = 0.1711$; for Area of some, $n = 77$ cells for *H3.3A*$^{flox/flox}$,*H3.3B*$^{-/-}$ mice and 150 cells for *Rosa*-Cre^{ERT2};*H3.3A*$^{flox/flox}$,*H3.3B*$^{-/-}$ mice, $P < 0.0001$). **d, e**, Representative confocal images and quantification of immunostaining using Iba1 in the hippocampus of *Cx3cr1*-Cre^{ERT2};*Hira*$^{wt/wt}$ (*n* = 5 mice) and *Cx3cr1*-Cre^{ERT2};*Hira*$^{flox/flox}$ mice (*n* = 5 mice) (Comparison by two-tailed unpaired Student's t test, $P_{No.\ of\ Iba1+\ cells} = 0.4314$; for Area of soma, $n = 94$ cells for *Cx3cr1*-Cre^{ERT2};*Hira*$^{wt/wt}$ and 104 cells for *Cx3cr1*-Cre^{ERT2};*Hira*$^{flox/flox}$ mice, $P = 0.1614$). **f-h**, Volcano plot showing differential gene expressions in *H3f3a/H3f3b* double knockout (*H3.3* DKO) RAW264.7 macrophage cell lines in comparison with control RAW264.7 cells after 0h-, 1h-, and 2h-LPS stimulation (data from[56]). The significance (adjusted *P*-value) was derived by DESeq2 with a two-sided Wald test and then adjusted for multiple testing using the Benjamini–Hochberg procedure. The adjusted *P*-value and the fold-change are converted to -log10 (adjusted *P*-value) and log2 (fold change), respectively. The vertical and horizontal dotted lines show the cut-off of fold-change = ± 1.5, and of adjusted *P*-value = 0.01, respectively. **i**, Genome browser visualization of RNA-seq around *Ccnd2* loci in *Cx3cr1*-Cre^{ERT2};*Daxx*$^{wt/wt}$ microglia, *Cx3cr1*-Cre^{ERT2};*Daxx*$^{flox/flox}$ microglia, *H3.3* DKO RAW264.7 cells and wild type control. **j**, Heatmap showing relative expression levels of different gene clusters associated with diverse microglia phenotypes (cell cycle, DNA damage response-DDR, p53/Cdkis, IFN response and senescence-associated secretory phenotype-SASP) in H3.3 DKO RAW264.7 macrophage compared to wild type control cells upon 0h-, 1h-, and 2h-LPS stimulation (data from GSE: 125159)[56]. **k**, Heatmap showing relative expression levels of different gene clusters associated with diverse microglia phenotypes (cell cycle, DNA damage response-DDR, p53/Cdkis, IFN response and senescence-associated secretory phenotype-SASP) in *Trim28* knockout CD8+ T cells (T-cell, data from[54]) or neural progenitor cells (NPC, data from[53]) vs control cells. Data are presented as mean ± s.e.m. Asterisks (*) indicate statistical significance: *$P < 0.05$, **$P < 0.01$ and ***$P < 0.001$; n.s. indicates not significant.

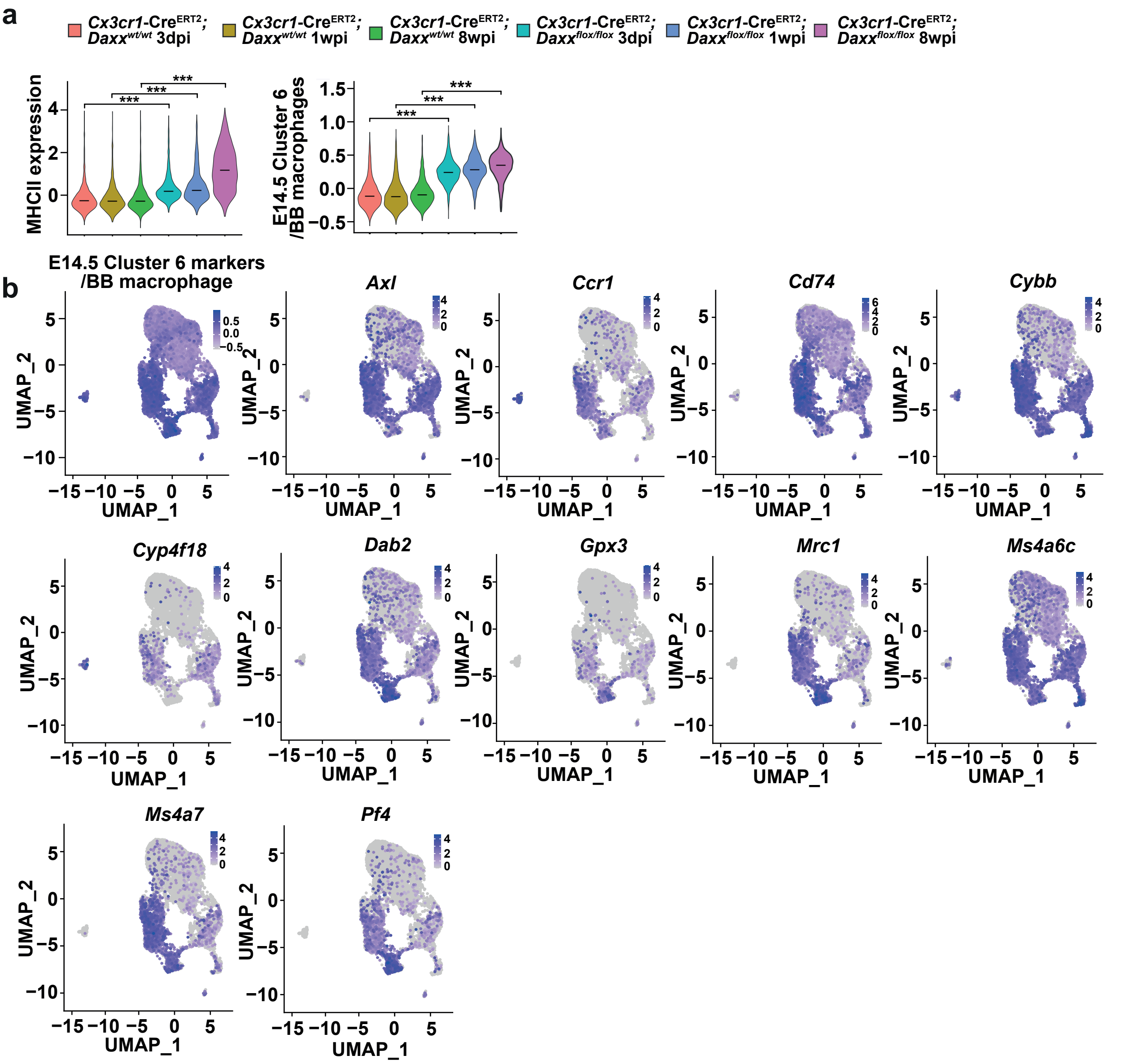

Supplementary Fig. 6. Microglia lacking *Daxx* show induction of DAM signatures over time. **a**, Violin plots using single cell RNA-seq data showing gene expression of MHCII, and E14.5 brain Cluster 6/border macrophages signatures[46] in *in vivo* microglia from *Cx3cr1*-Cre^{ERT2};*Daxx*$^{wt/wt}$ and *Cx3cr1*-Cre^{ERT2};*Daxx*$^{flox/flox}$ mice. The black line indicates the median expression/module score calculated using the AddModuleScore() function in Seurat. Statistical significance was assessed by pairwise two-sided Wilcoxon rank-sum tests using the geom_signif function (ggsignif R package) for the given comparisons, no correction for multiple testing; for MHCII expression, $P_{3dpi} < 2.22e-16$, $P_{1wpi} < 2.22e-16$, $P_{8wpi} < 2.22e-16$; for E14.5 Cluster 6, $P_{3dpi} < 2.22e-16$, $P_{1wpi} < 2.22e-16$, $P_{8wpi} < 2.22e-16$. **b**, UMAP plots showing E14.5 embryonic microglia Cluster 6 feature[46] as well as *Axl*, *Ccr1*, *Cd74, Cybb, cyp4f18, Dab2, Gpx3, Mrc1, Ms4a6c, Ms4a7* and *Pf4* in the microglia across all clusters. Asterisks (*) indicate statistical significance: *$P < 0.05$, **$P < 0.01$ and ***$P < 0.001$.

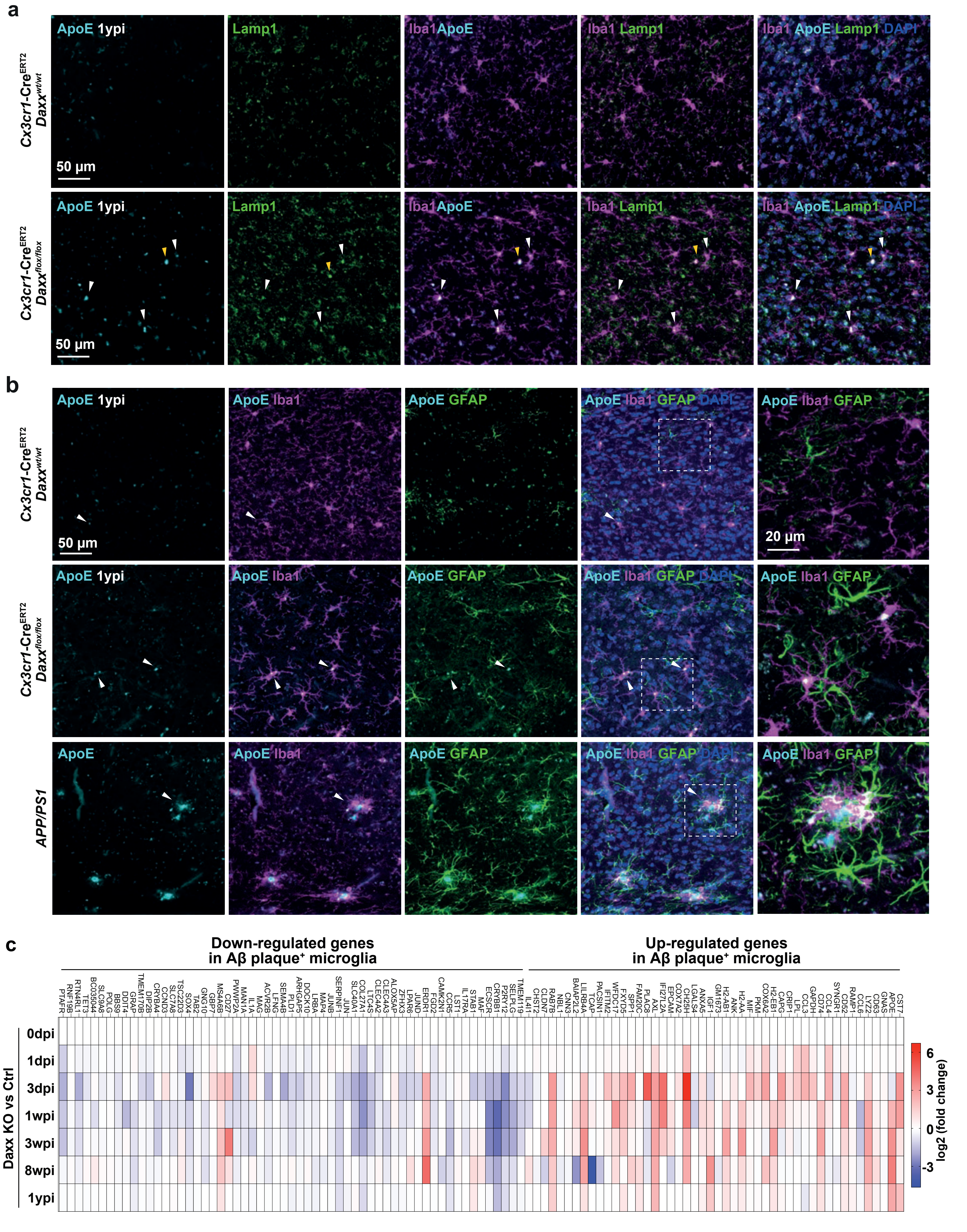

Supplementary Fig. 7. Loss of *Daxx* causes accumulation of ApoE in microglia but not in astrocytes, in part recapitulating gene expression changes found in plaque-associated ApoE-positive microglia. a, Representative confocal images of Iba1, ApoE and Lamp1 triple immunostaining in the cortex of *Cx3cr1*-Cre^{ERT2};*Daxx*$^{flox/flox}$ and *Cx3cr1*-Cre^{ERT2};*Daxx*$^{wt/wt}$ mice at 1ypi (*n* = 3 mice for each genotype); White arrowheads indicate the colocalization of ApoE and Lamp1 in the cell body of microglia; yellow arrowheads indicate the colocalization of ApoE and Lamp1 in the dendrite of microglia. **b**, Representative confocal images of Iba1, ApoE and GFAP triple immunostaining in the cortex of *Cx3cr1*-Cre^{ERT2};*Daxx*$^{flox/flox}$ and *Cx3cr1*-Cre^{ERT2};*Daxx*$^{wt/wt}$ mice at 1ypi and *APP/PS1* mice at 9-month-old (*n* = 3 mice for each genotype); Right panel showing high-magnification images in dash box; Arrowheads indicate accumulated ApoE protein in microglia. **c**, Heatmap showing relative expression levels of different gene clusters associated with Aβ plaque+ microglia[57] in microglia isolated from *Cx3cr1*-Cre^{ERT2};*Daxx*$^{flox/flox}$ (*Daxx* KO) vs *Cx3cr1*-Cre^{ERT2};*Daxx*$^{wt/wt}$ (Ctrl) mice from 0dpi to 1ypi.

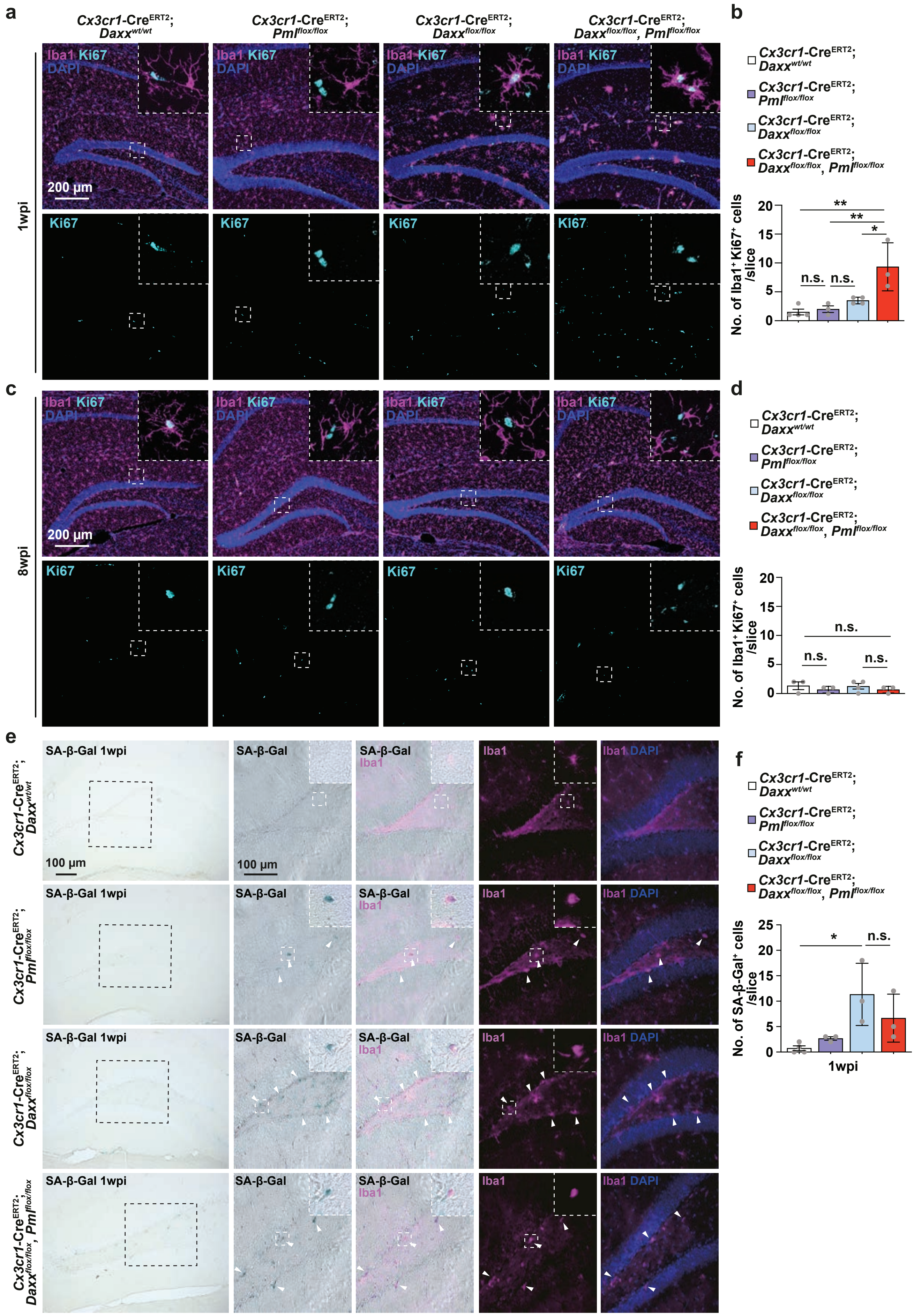

a
Cx3cr1-CreERT2; Daxxwt/wt
Cx3cr1-CreERT2; Pmlflox/flox
Cx3cr1-CreERT2; Daxxflox/flox
Cx3cr1-CreERT2; Daxxflox/flox, Pmlflox/flox
Iba1 Ki67 DAPI
Ki67
200 μm
1wpi
b
No. of Iba1+Ki67+ cells /slice
n.s.
**
*
c
8wpi
d
e
SA-β-Gal 1wpi
SA-β-Gal
SA-β-Gal Iba1
Iba1
Iba1 DAPI
100 μm
f
No. of SA-β-Gal+ cells /slice
1wpi

Supplementary Fig. 8. Effect of concomitant deletion of *Pml* and *Daxx* on microglial proliferation. a, b, Representative confocal images and quantification of Iba1 and Ki67 immunostaining in the hippocampus of *Cx3cr1*-Cre^{ERT2};$Daxx^{wt/wt}$ (WT, *n* = 3 mice), *Cx3cr1*-Cre^{ERT2};$Pml^{flox/flox}$ (*Pml* KO, *n* = 4 mice), *Cx3cr1*-Cre^{ERT2};$Daxx^{flox/flox}$ (*Daxx* KO, *n* = 4 mice), and *Cx3cr1*-Cre^{ERT2};$Daxx^{flox/flox}$,$Pml^{flox/flox}$ (*Daxx/Pm*l DKO, *n* = 3 mice) mice at 1wpi (Comparison by ordinary one-way ANOVA, F = 10.09, P = 0.0023, Tukey's multiple comparisons test: $P_{WT\ vs\ Pml\ KO}$ = 0.9875, $P_{WT\ vs\ Daxx\ KO}$ = 0.5254, $P_{WT\ vs\ Daxx/Pml\ DKO}$ = 0.0022, $P_{Daxx\ KO\ vs\ Pml\ KO}$ = 0.7669, $P_{Pml\ KO\ vs\ Daxx/Pml\ DKO}$ = 0.0056, $P_{Daxx\ KO\ vs\ Daxx/Pml\ DKO}$ = 0.0158). Inserted image in the upper-right of each image showing high-magnification image in dashed box. **c, d**, Representative confocal images and quantification of Iba1 and Ki67 immunostaining in the hippocampus of *Cx3cr1*-Cre^{ERT2};$Daxx^{wt/wt}$ (*n* = 3 mice), *Cx3cr1*-Cre^{ERT2};$Pml^{flox/flox}$ (*n* = 3 mice), *Cx3cr1*-Cre^{ERT2};$Daxx^{flox/flox}$ (n = 4 mice), and *Cx3cr1*-Cre^{ERT2};$Daxx^{flox/flox}$,$Pml^{flox/flox}$ (*n* = 3 mice) mice at 8wpi (Comparison by ordinary one-way ANOVA, F = 0.5556, P = 0.6573, Tukey's multiple comparisons test: $P_{WT\ vs\ Pml\ KO}$ = 0.9992, $P_{WT\ vs\ Daxx\ KO}$ = 0.7835, $P_{Daxx\ KO\ vs\ Daxx/Pml\ DKO}$ > 0.9999). Inserted image in the upper-right of each image showing high-magnification image in dashed box. **e, f**, Representative confocal images and quantification of co-staining for beta-galactosidase activity and Iba1 in the hippocampus of *Cx3cr1*-Cre^{ERT2};$Daxx^{wt/wt}$ (*n* = 4 mice), *Cx3cr1*-Cre^{ERT2};$Pml^{flox/flox}$ (*n* = 3 mice), *Cx3cr1*-Cre^{ERT2};$Daxx^{flox/flox}$ (*n* = 3 mice), and *Cx3cr1*-Cre^{ERT2};$Daxx^{flox/flox}$,$Pml^{flox/flox}$ (*n* = 3 mice) mice at 1wpi (Comparison by ordinary one-way ANOVA, F = 5.31, P = 0.0222, Tukey's multiple comparisons test: $P_{WT\ vs\ Pml\ KO}$ = 0.9023, $P_{WT\ vs\ Daxx\ KO}$ = 0.0195, $P_{Daxx\ KO\ vs\ Daxx/Pml\ DKO}$ = 0.4512). Black dashed boxes in the left panel identify high-magnification images of the second panel. Inserted image in the upper-right of each panel showing high-magnification image in white dashed box. Arrowheads indicate the colocalization of SA-β-Gal signal and $Iba1^+$ microglia. Data are presented as mean ± s.e.m. Asterisks (*) indicate statistical significance: *P < 0.05, **P < 0.01 and ***P < 0.001; n.s. indicates not significant.

Supplementary Fig. 1a

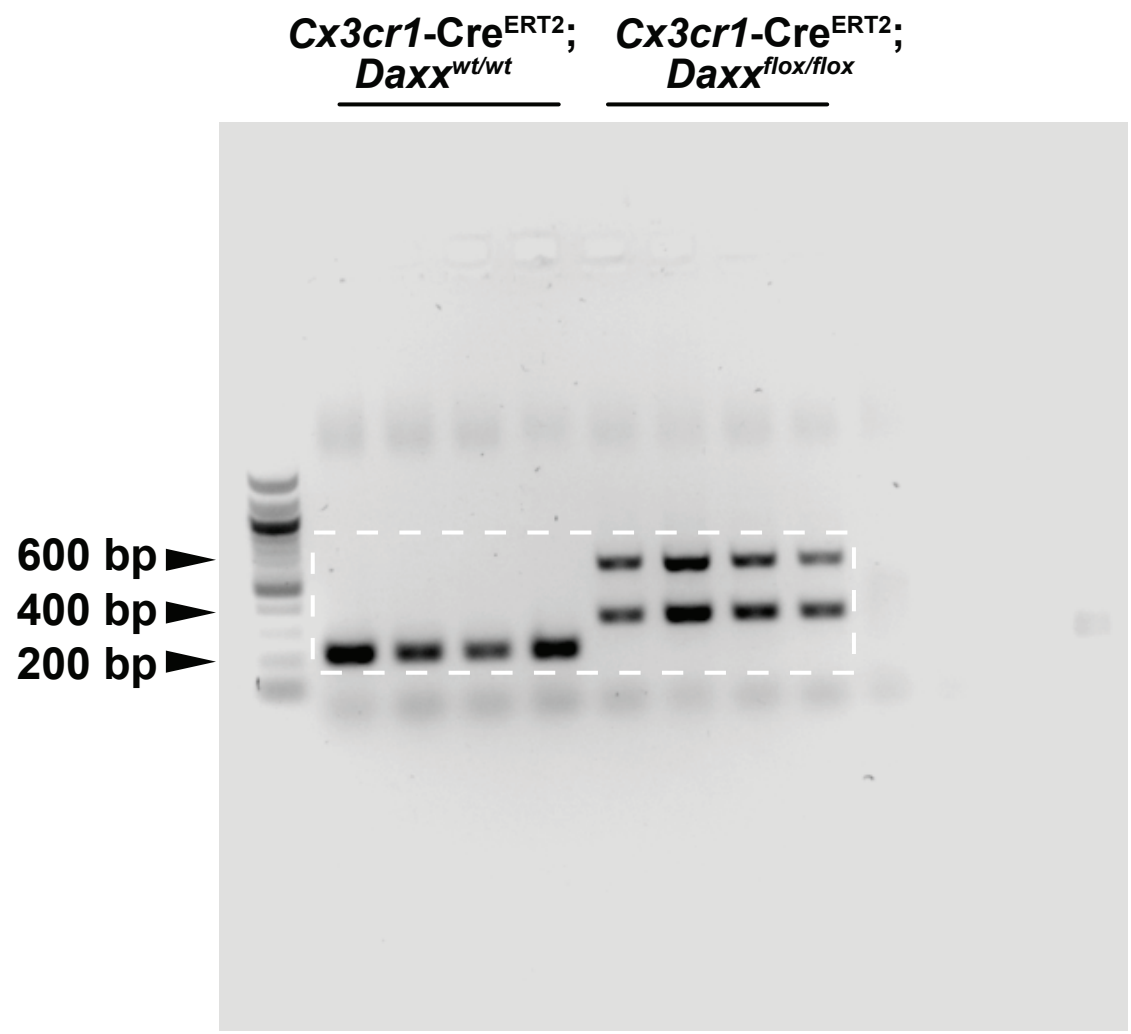

Supplementary Fig. 1b

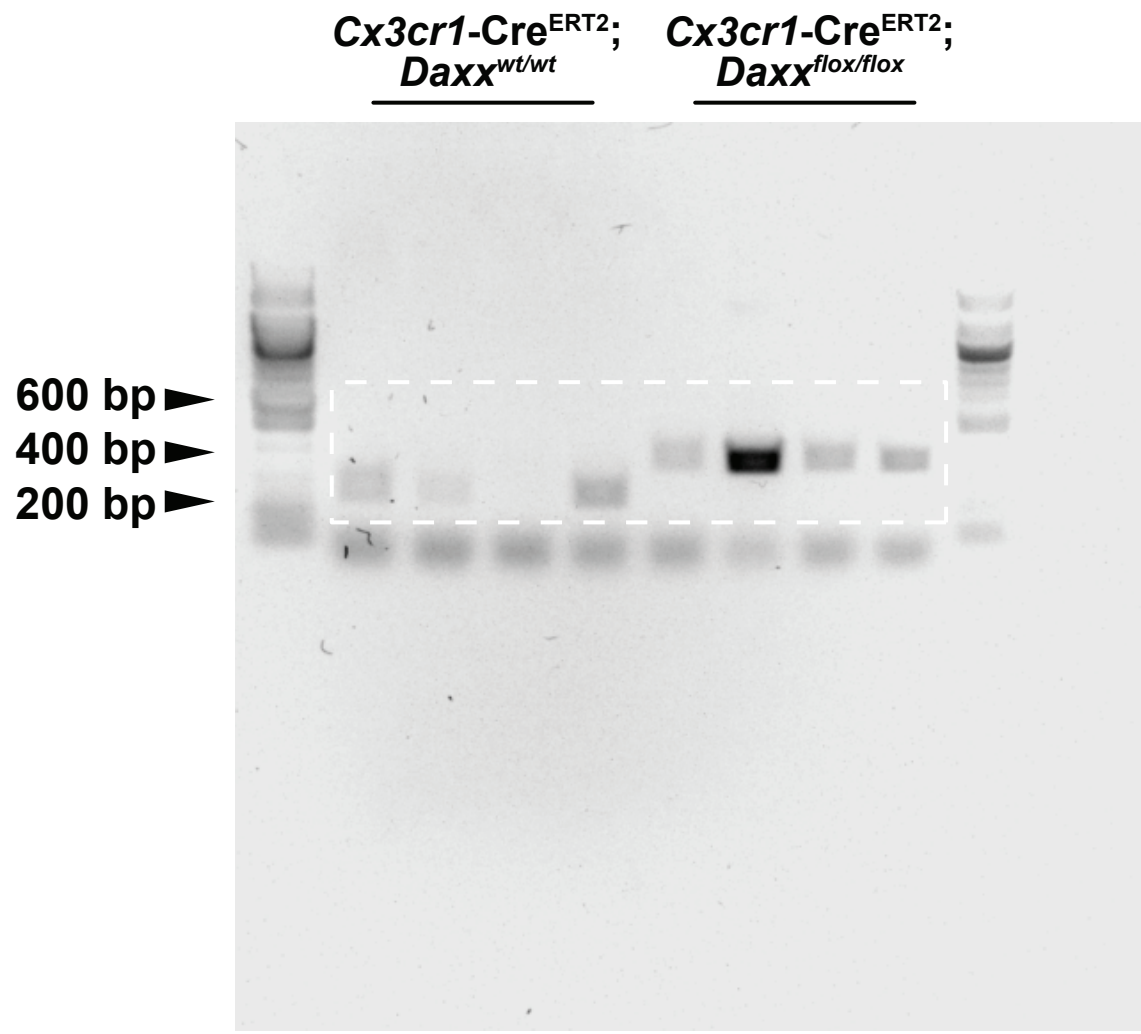

Supplementary Fig. 2f

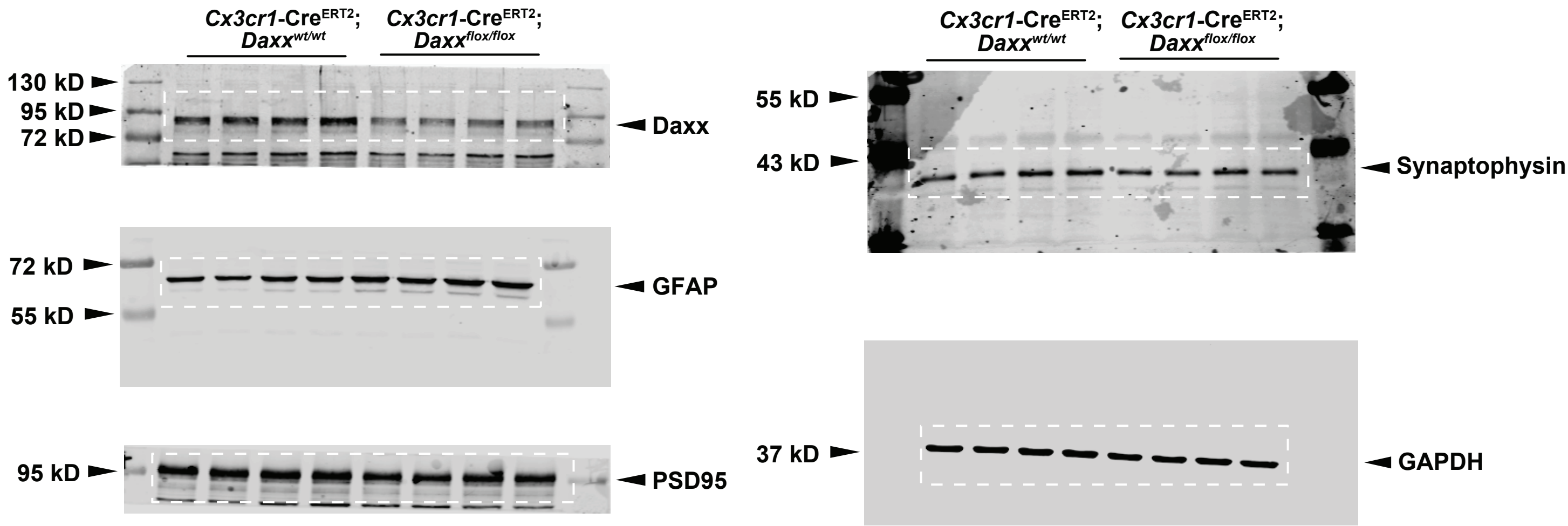

Note: Dashed white lines identify cropped areas

Supplementary Fig. 9. Unprocessed gels and blots corresponding to Supplementary Fig. 1 and 2.
