# Supplementary material for: Microglia activation by derepression of endogenous retroviruses drives inflammation and cellular senescence
Source: Nat Neurosci. 2026 Aug 17;29(10):2480–98. doi: 10.1038/s41593-026-02404-y (PMC13633122; doi:10.1038/s41593-026-02404-y)
Supplement: Supplementary file 2 [file 41593_2026_2404_MOESM22_ESM.pdf]

Fig. 1e

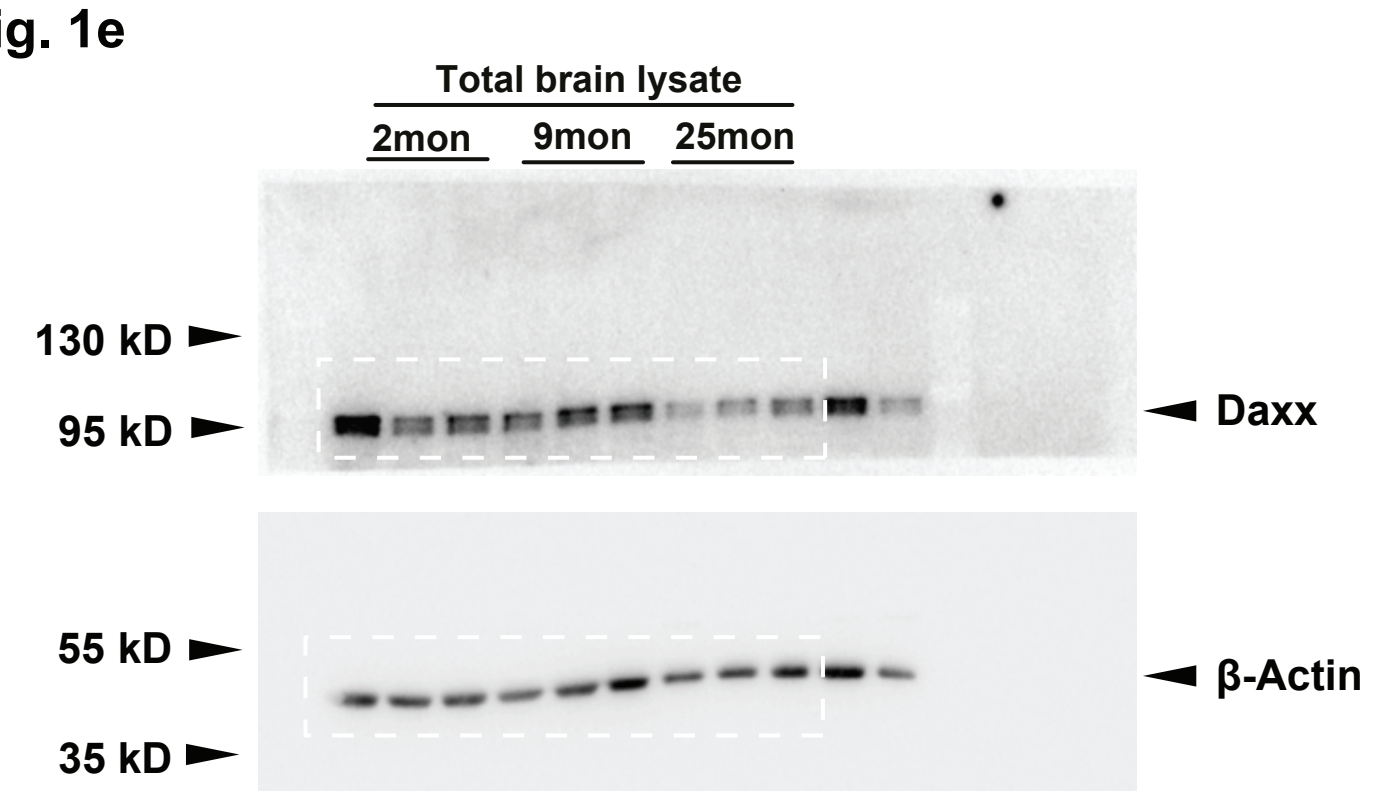

Fig. 1e

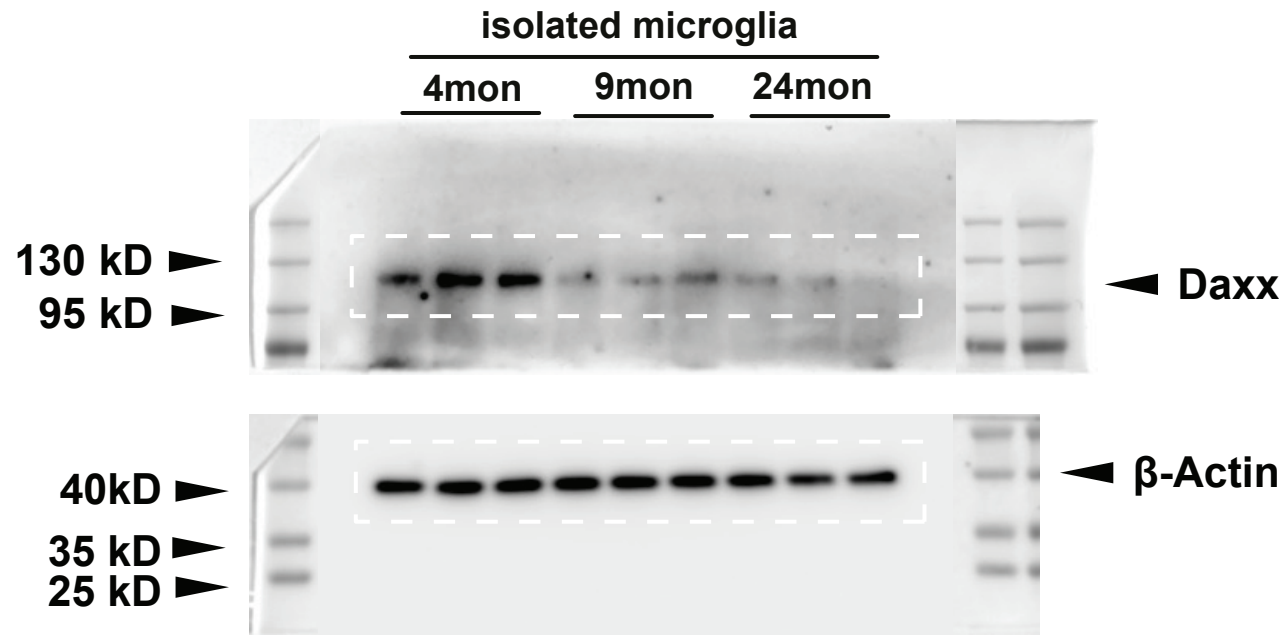

Fig.3b

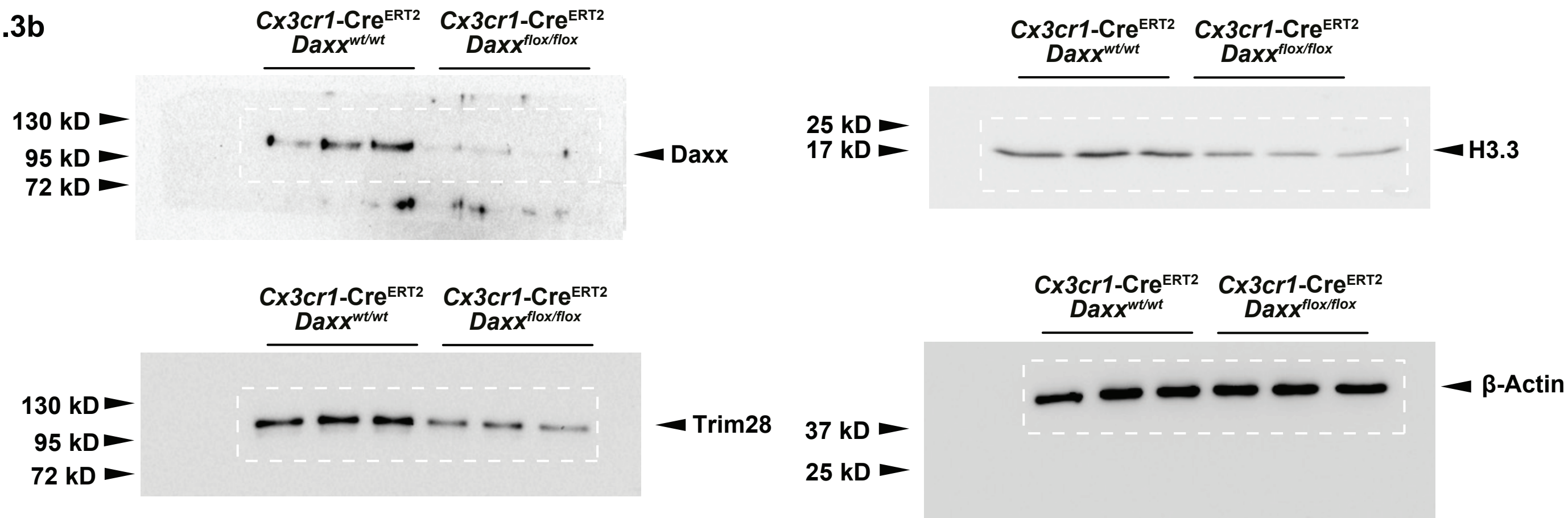

Fig. 3c

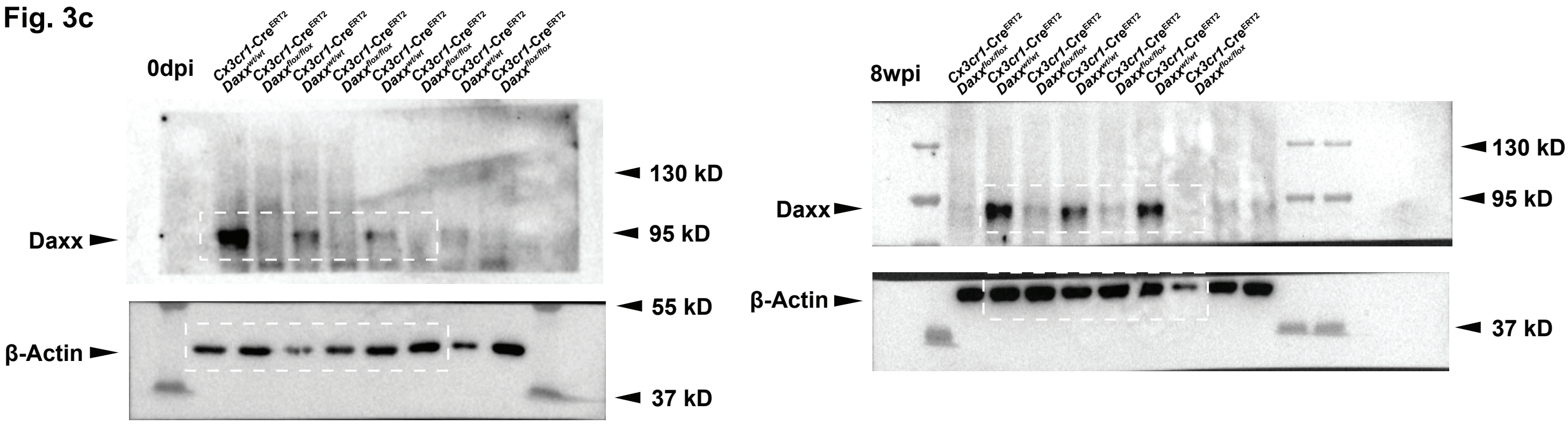

Note: Dashed white lines identify cropped areas

Fig. 8b

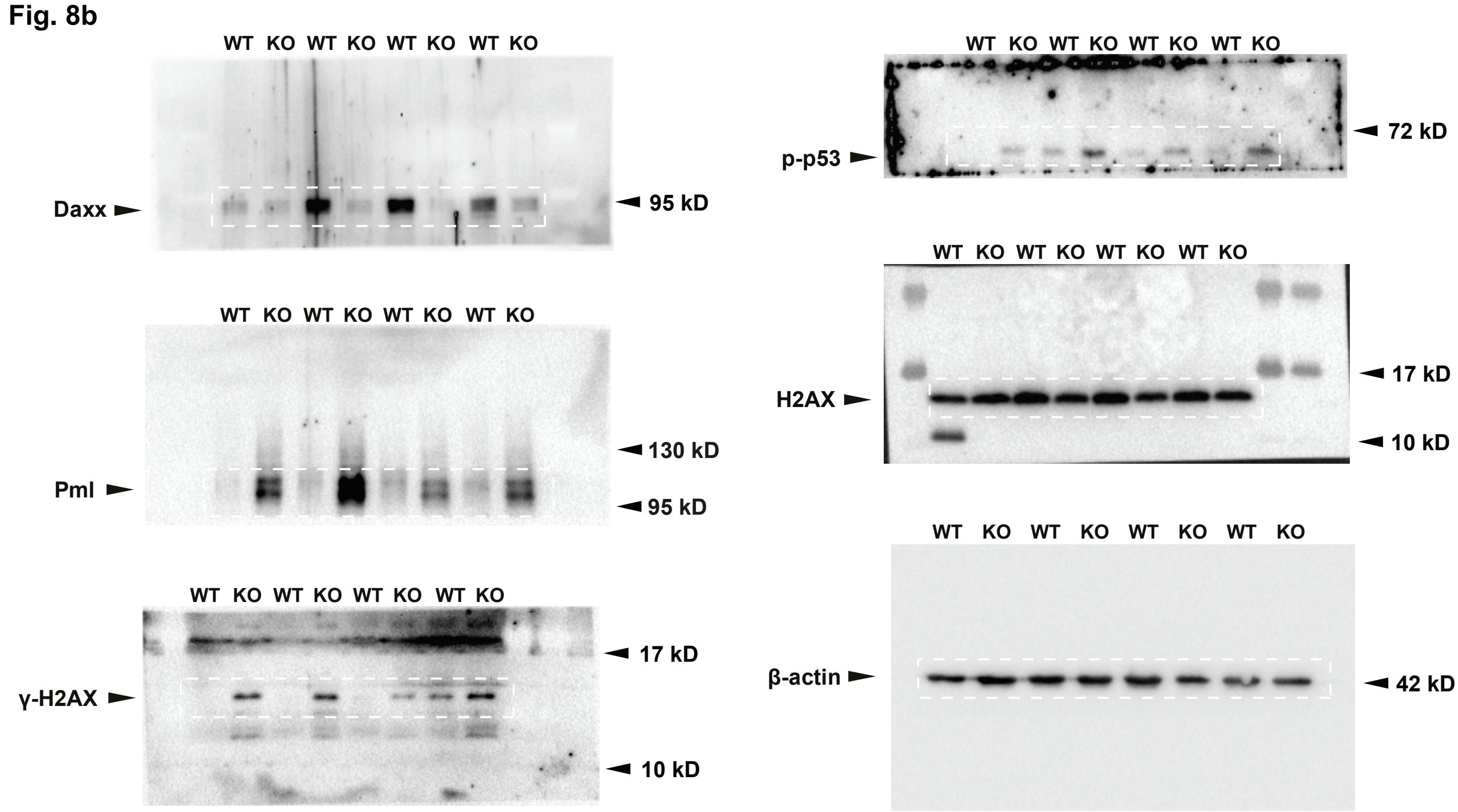

Extended Data Fig. 1f

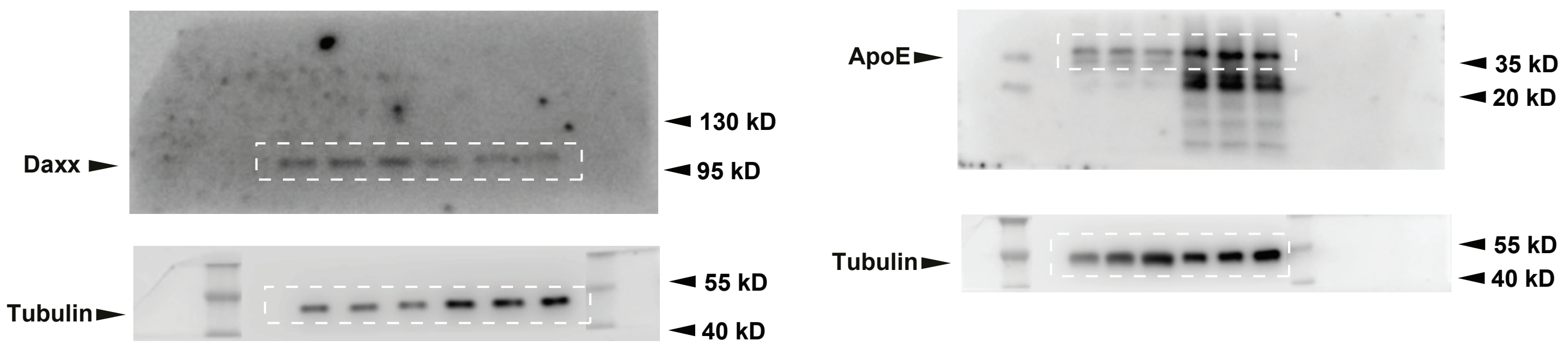

Note: Dashed white lines identify cropped areas

Extended Data Fig.2g

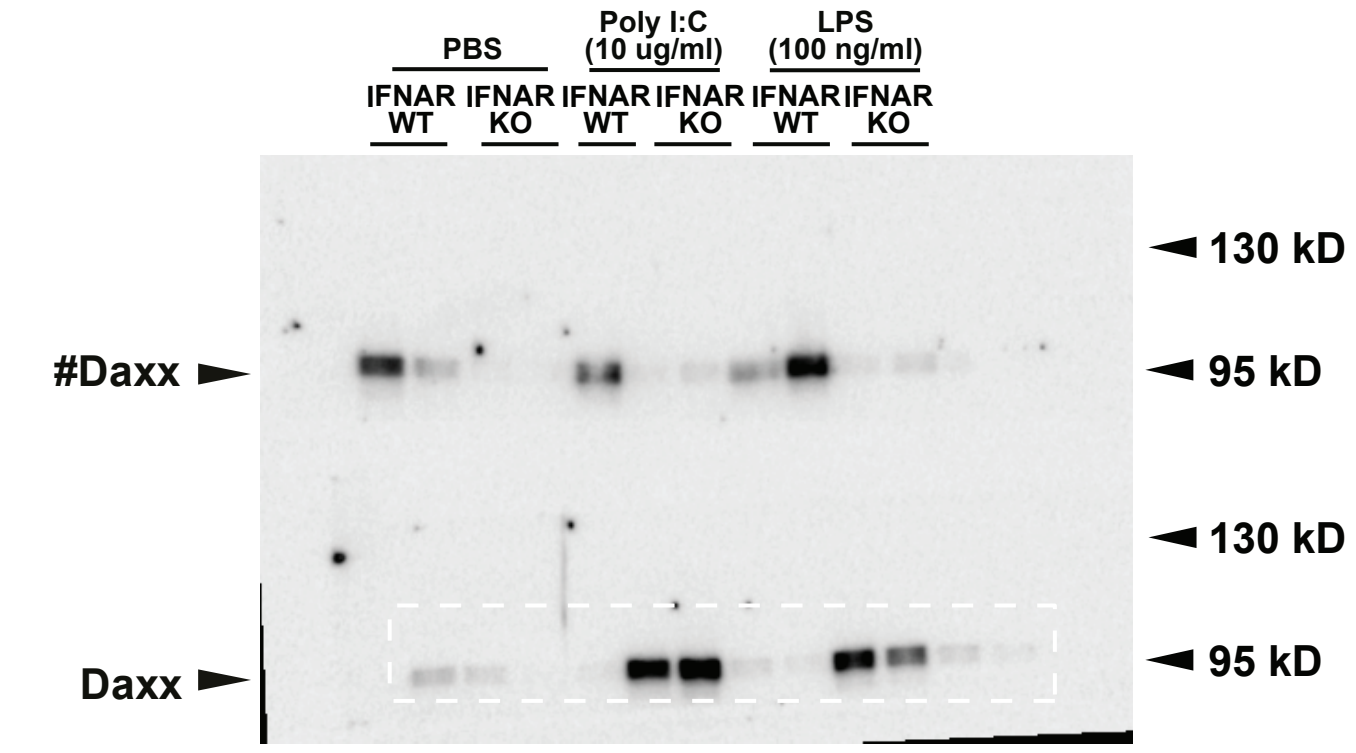

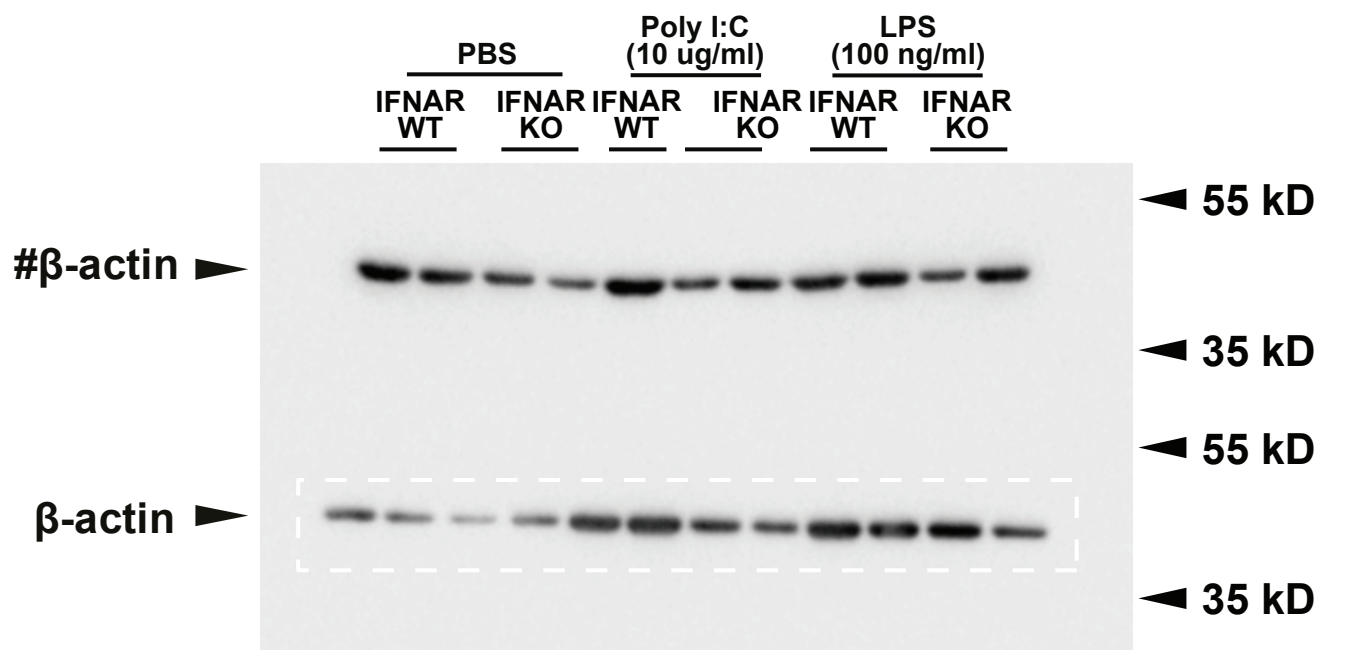

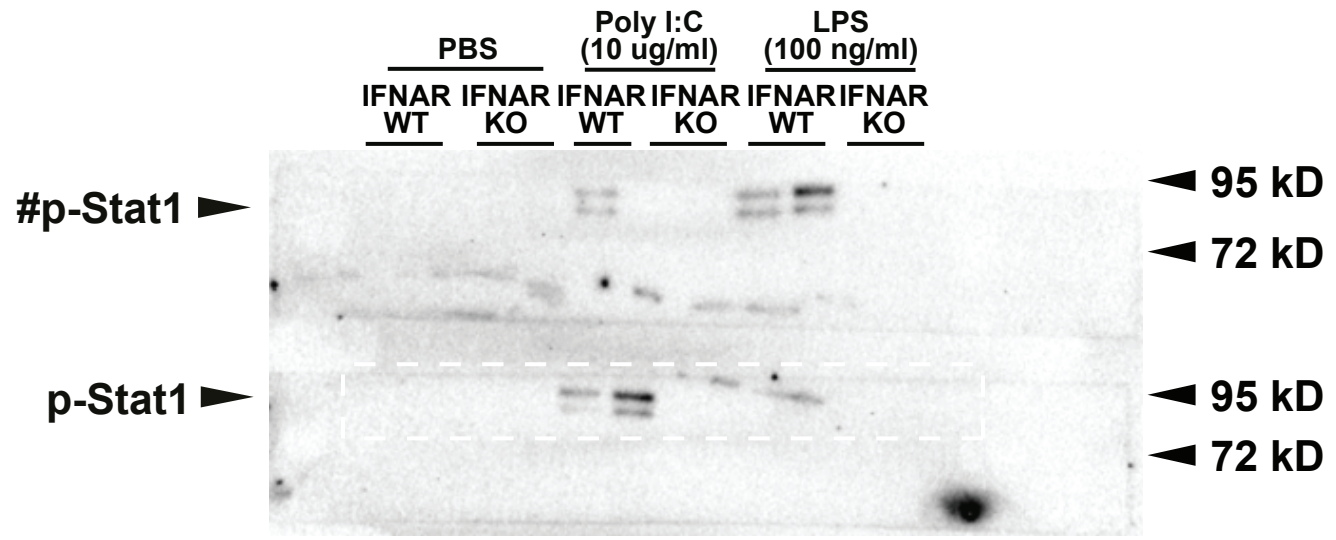

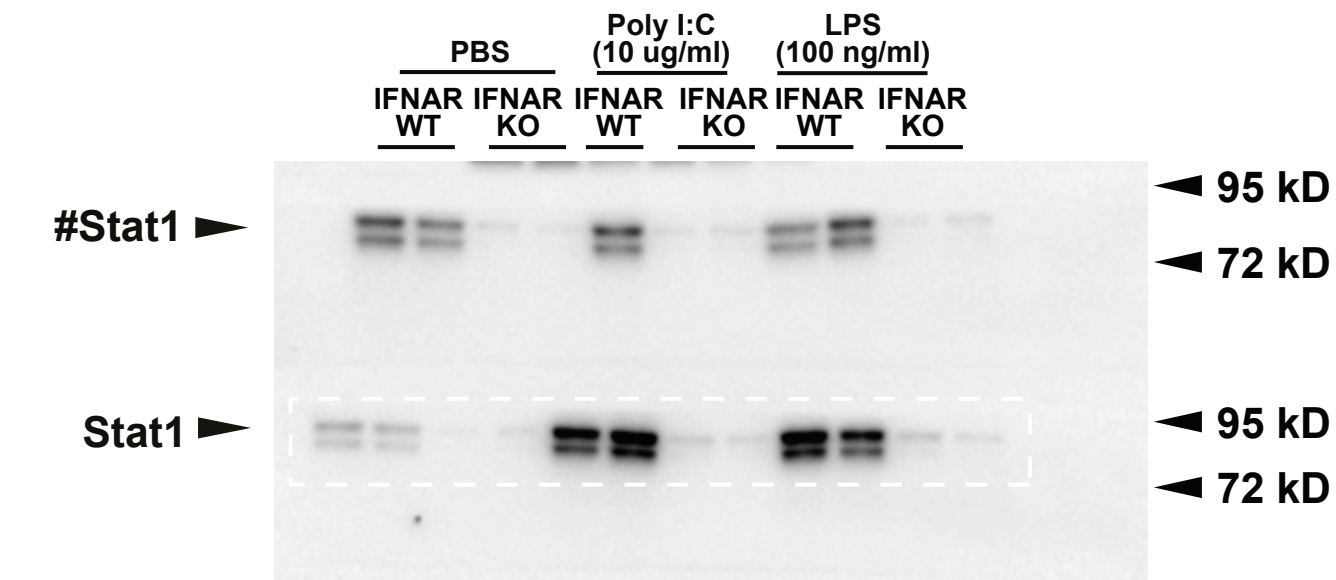

# additional panels shown above not in manuscript

Extended Dat Fig. 3a

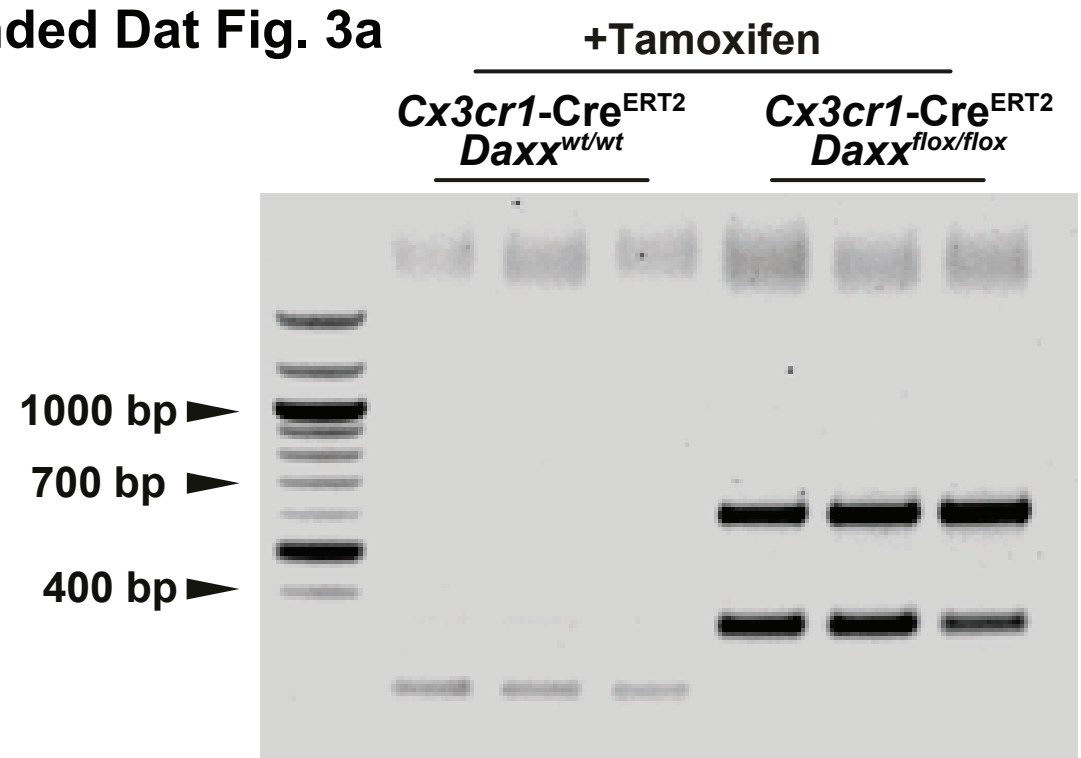

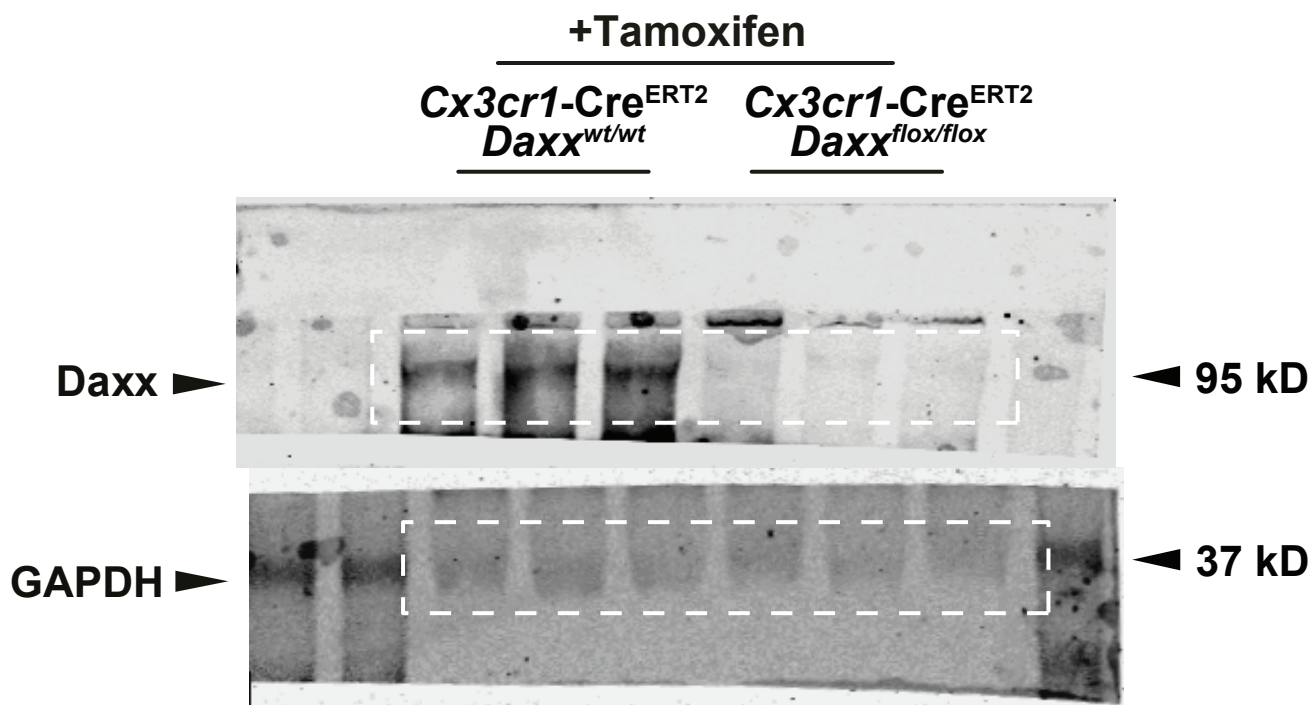

Extended Dat Fig. 3c

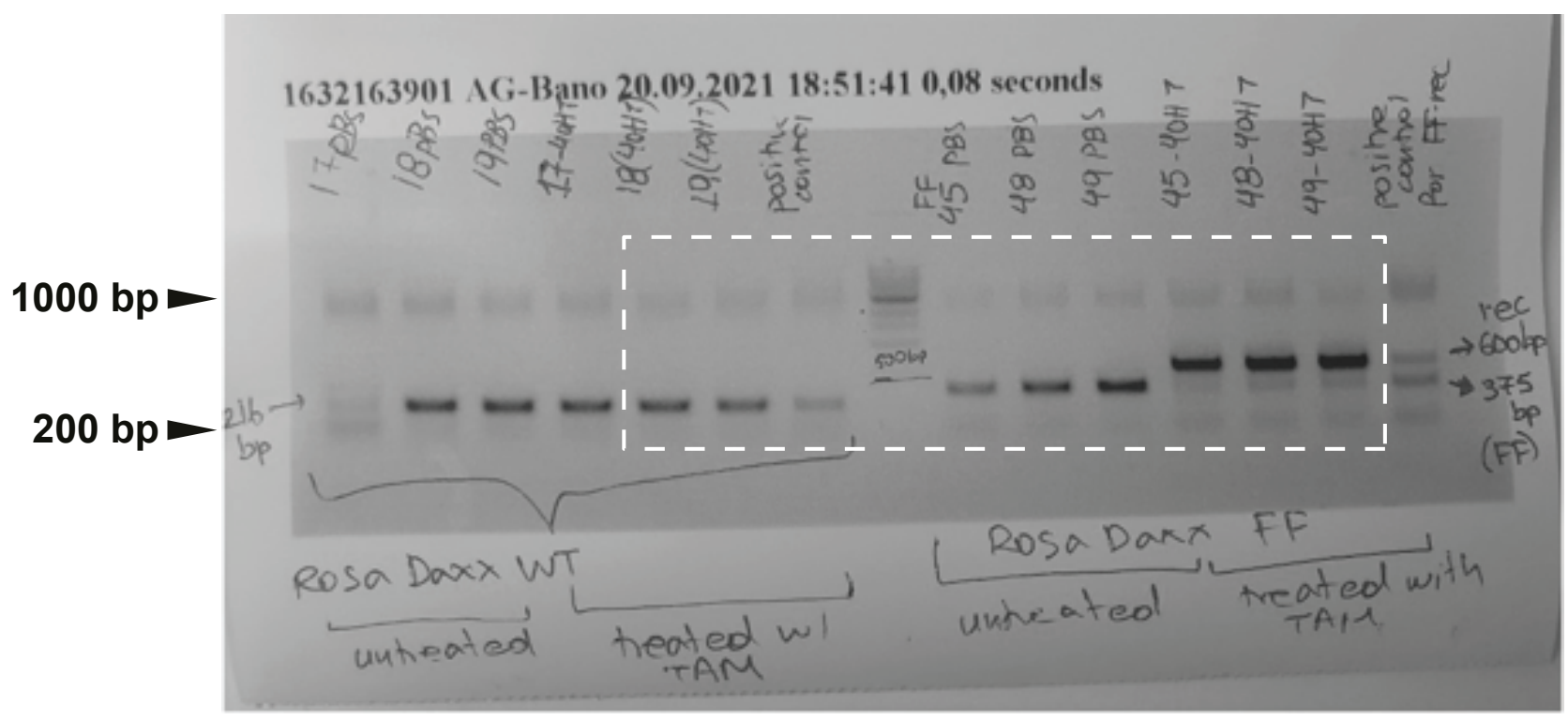

Note: Dashed white lines identify cropped areas

Extended Data Fig. 3e

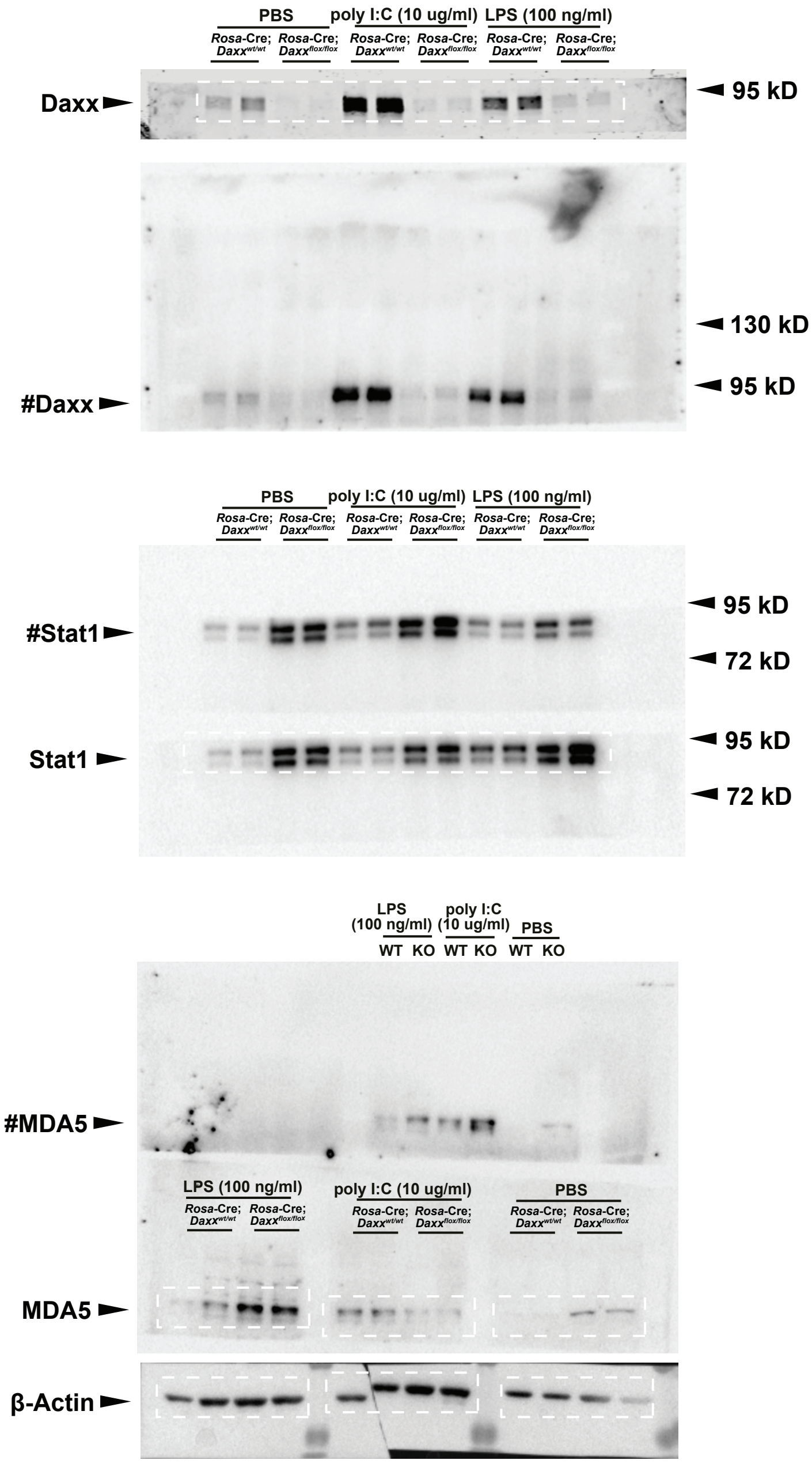

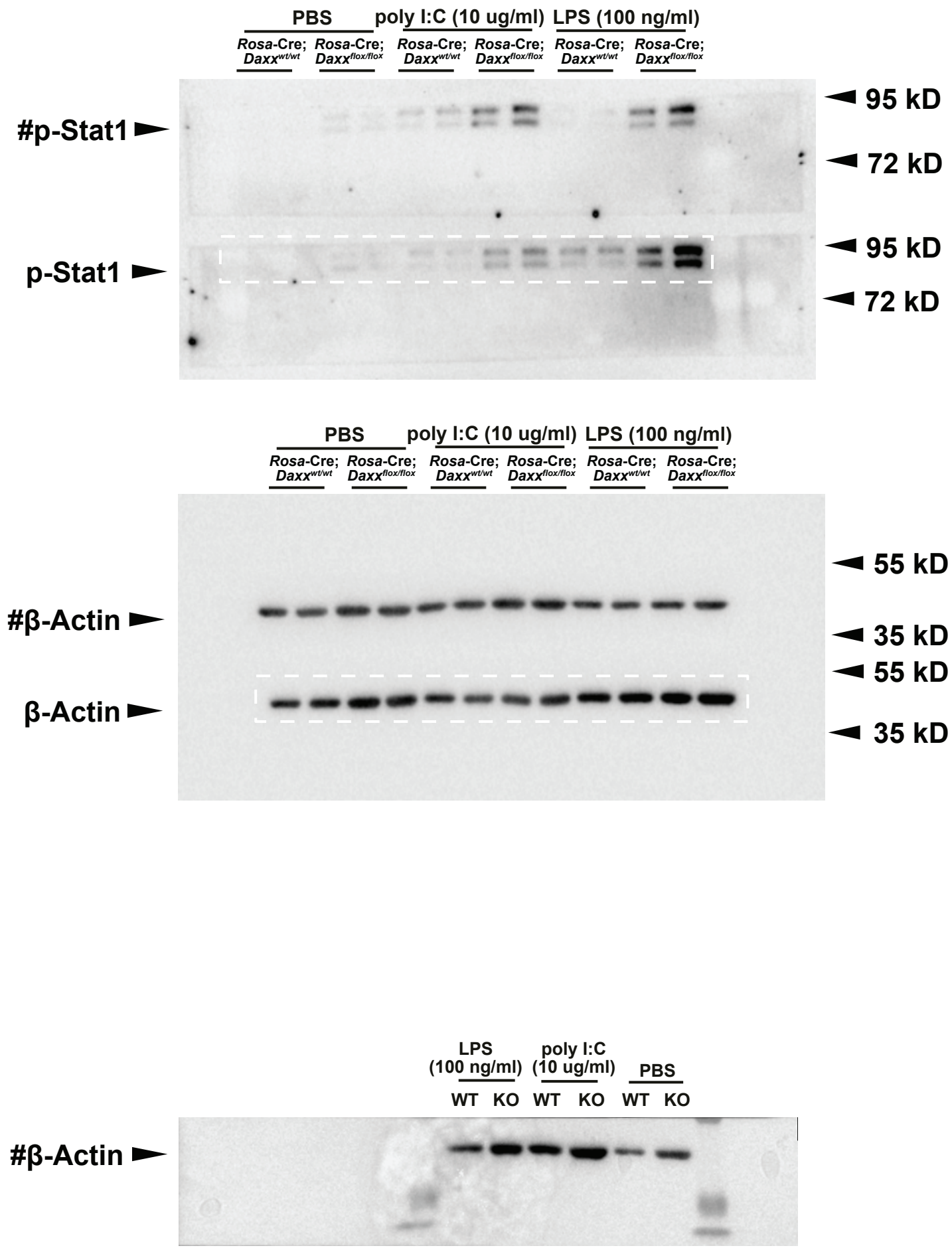

# additional panels shown above not in manuscript

Note: Dashed white lines identify cropped areas

Extended Data Fig. 3h

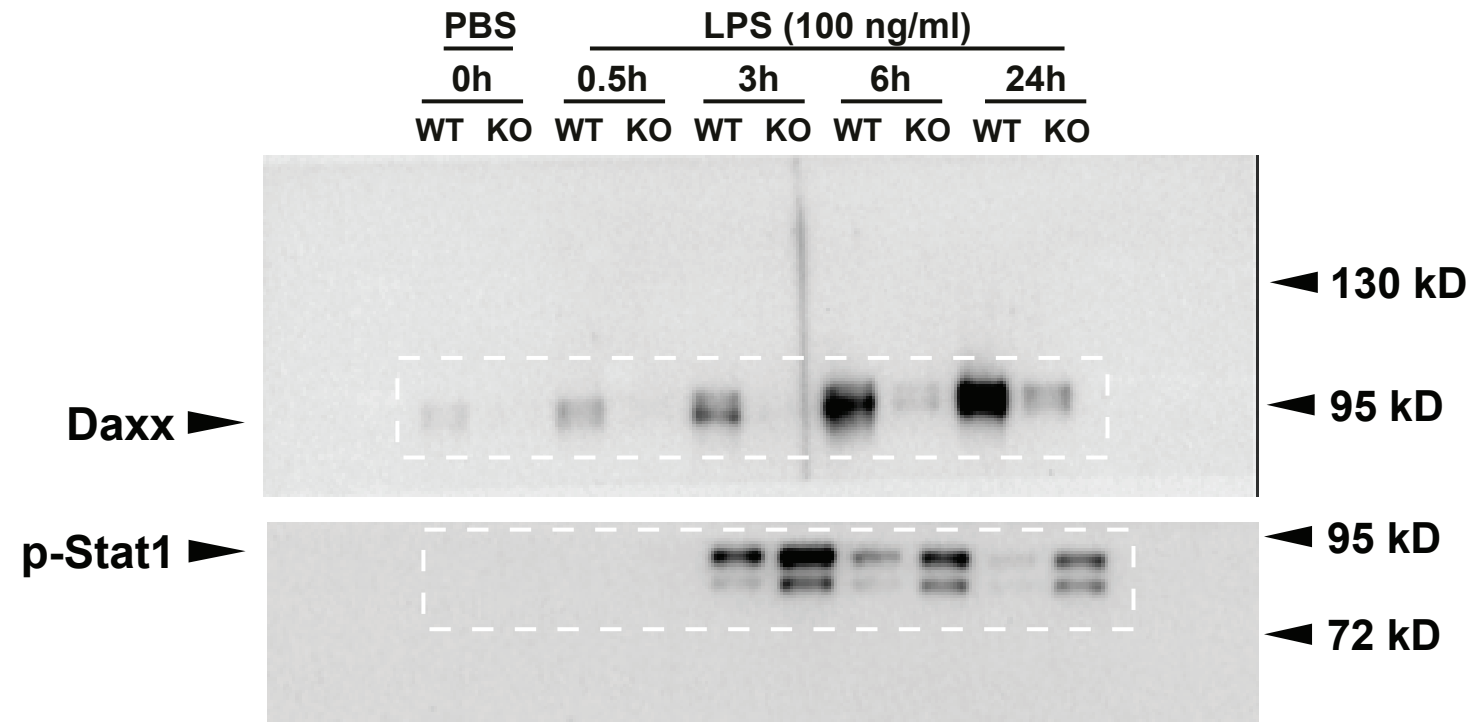

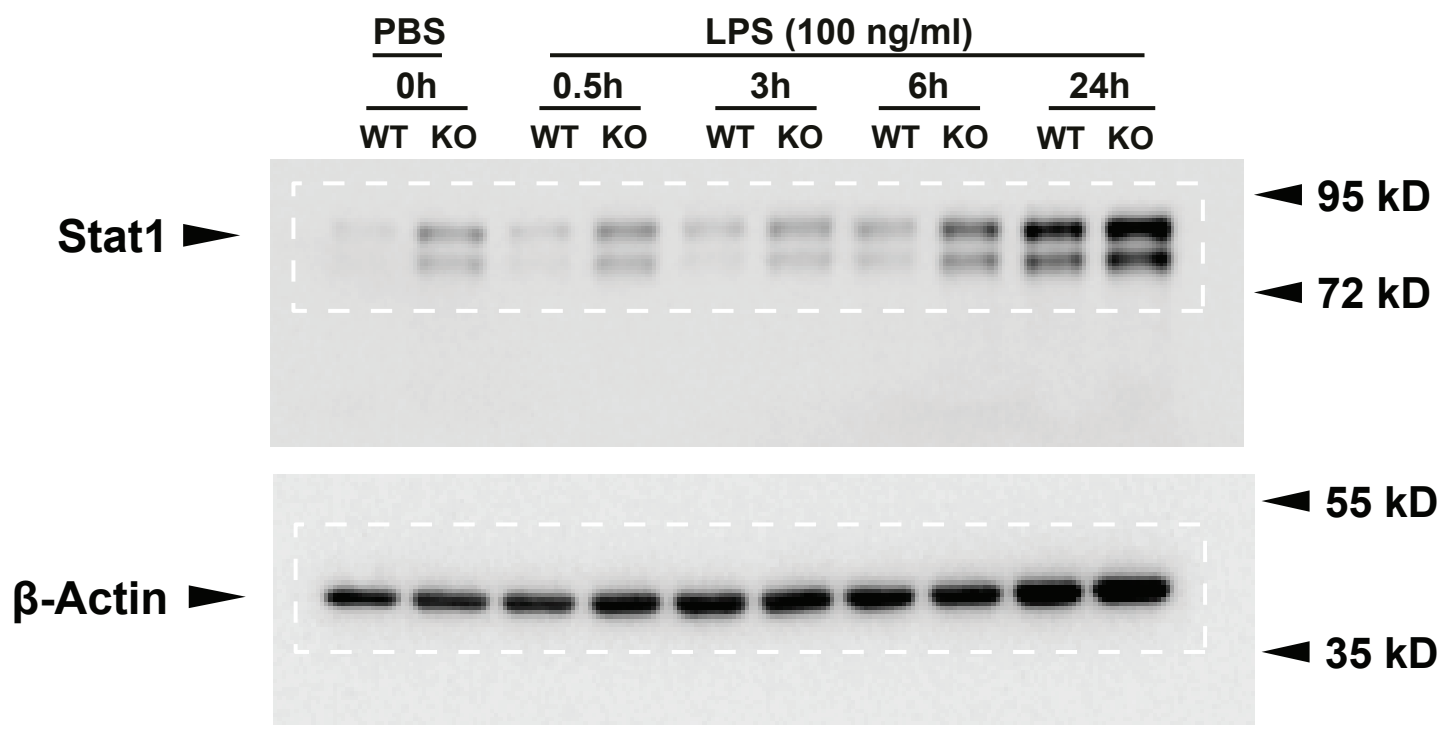

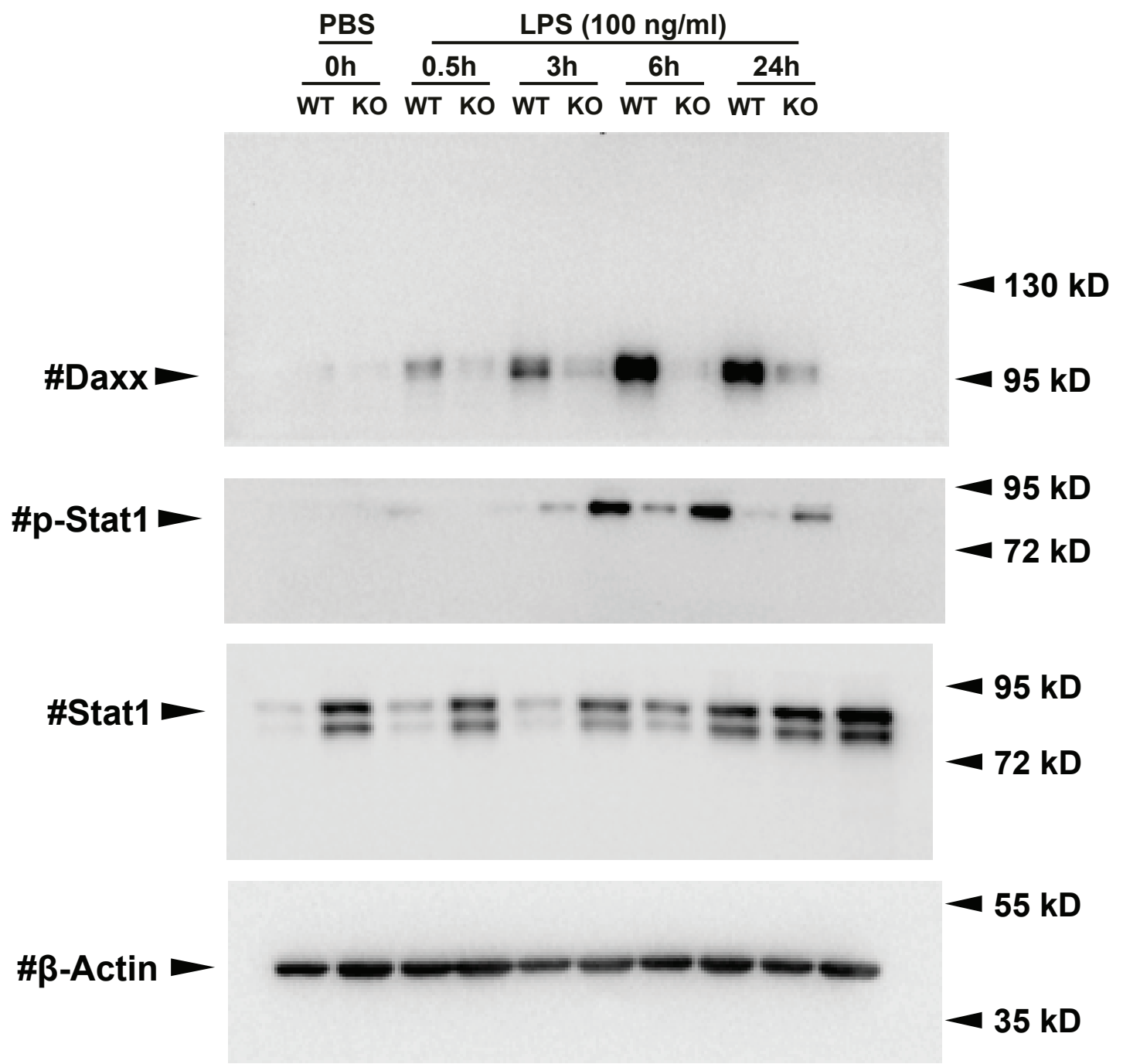

# additional panels shown above not in manuscript

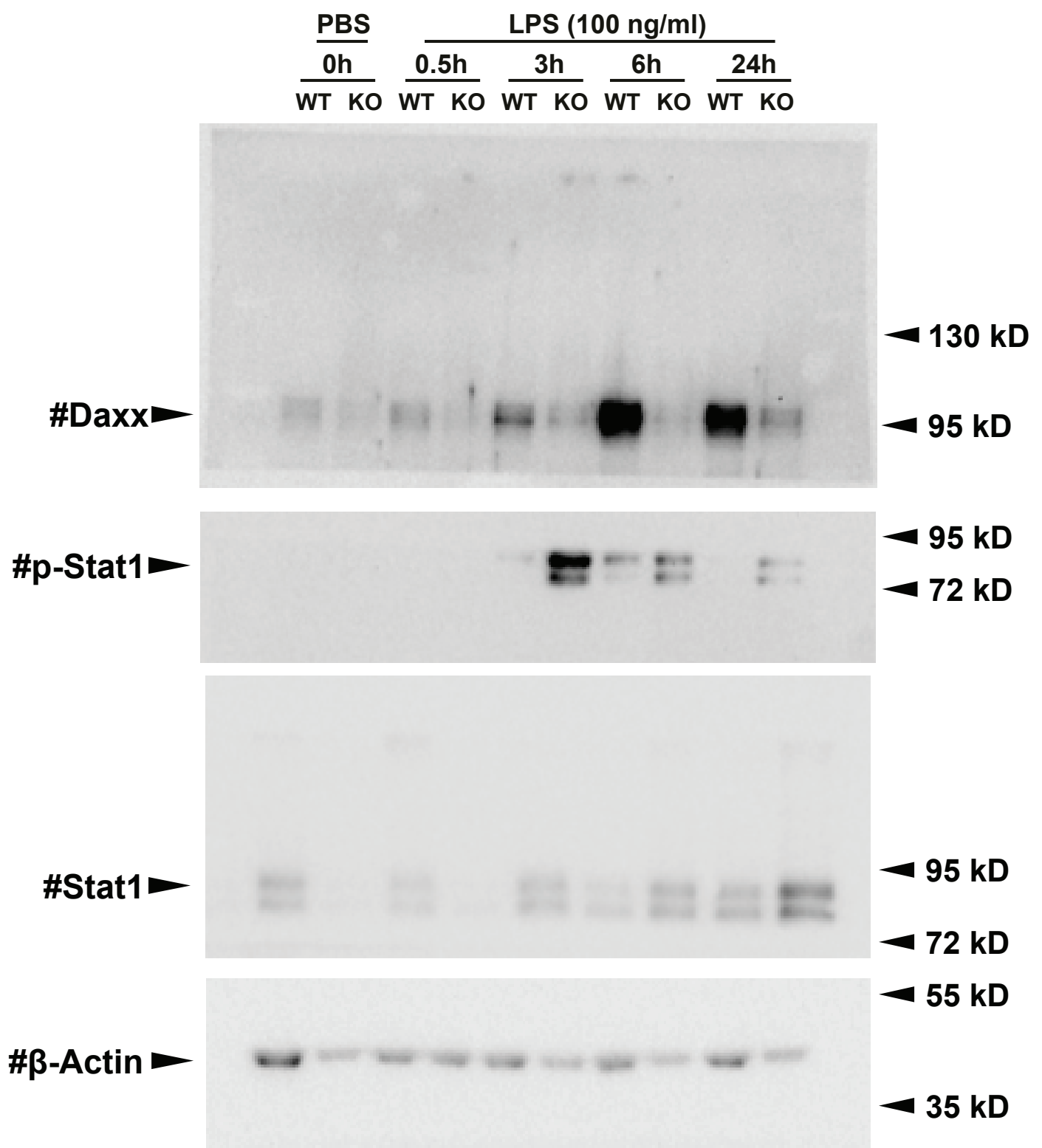

# additional panels shown above not in manuscript

Note: Dashed white lines identify cropped areas

Extended Data Fig. 6a

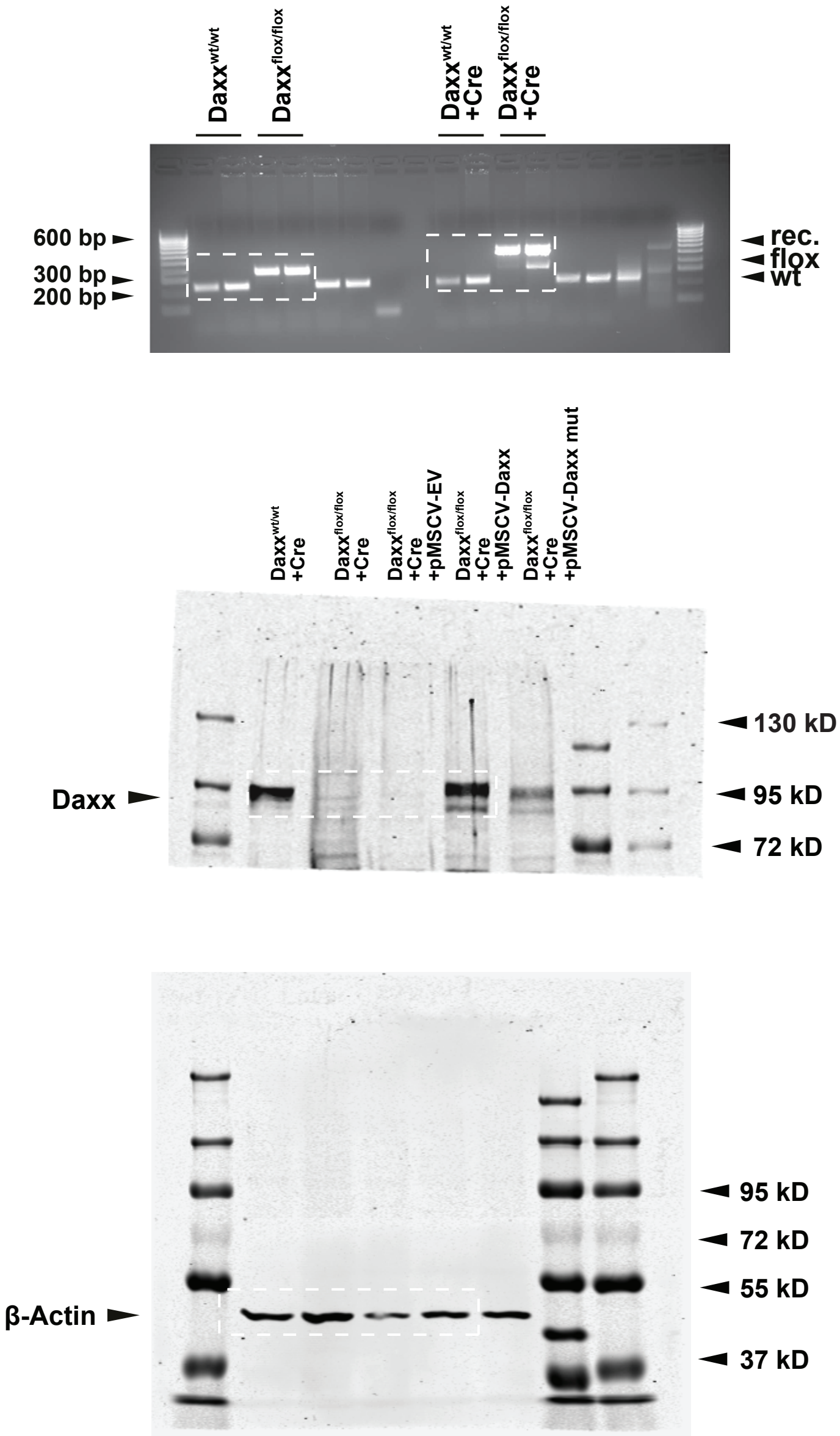

Note: Dashed white lines identify cropped areas

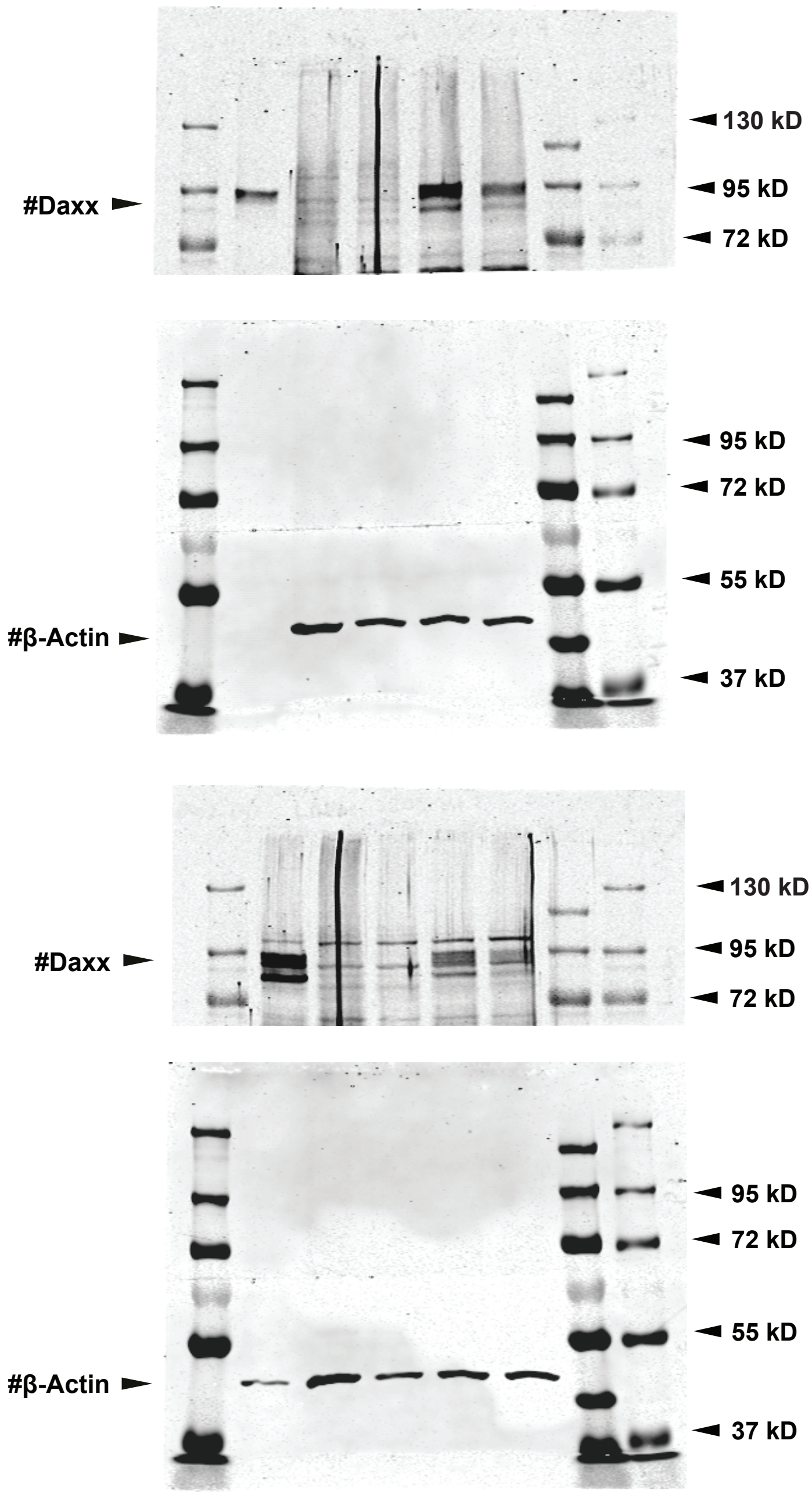

additional panels shown above not in manuscript

Extended Data Fig. 7f

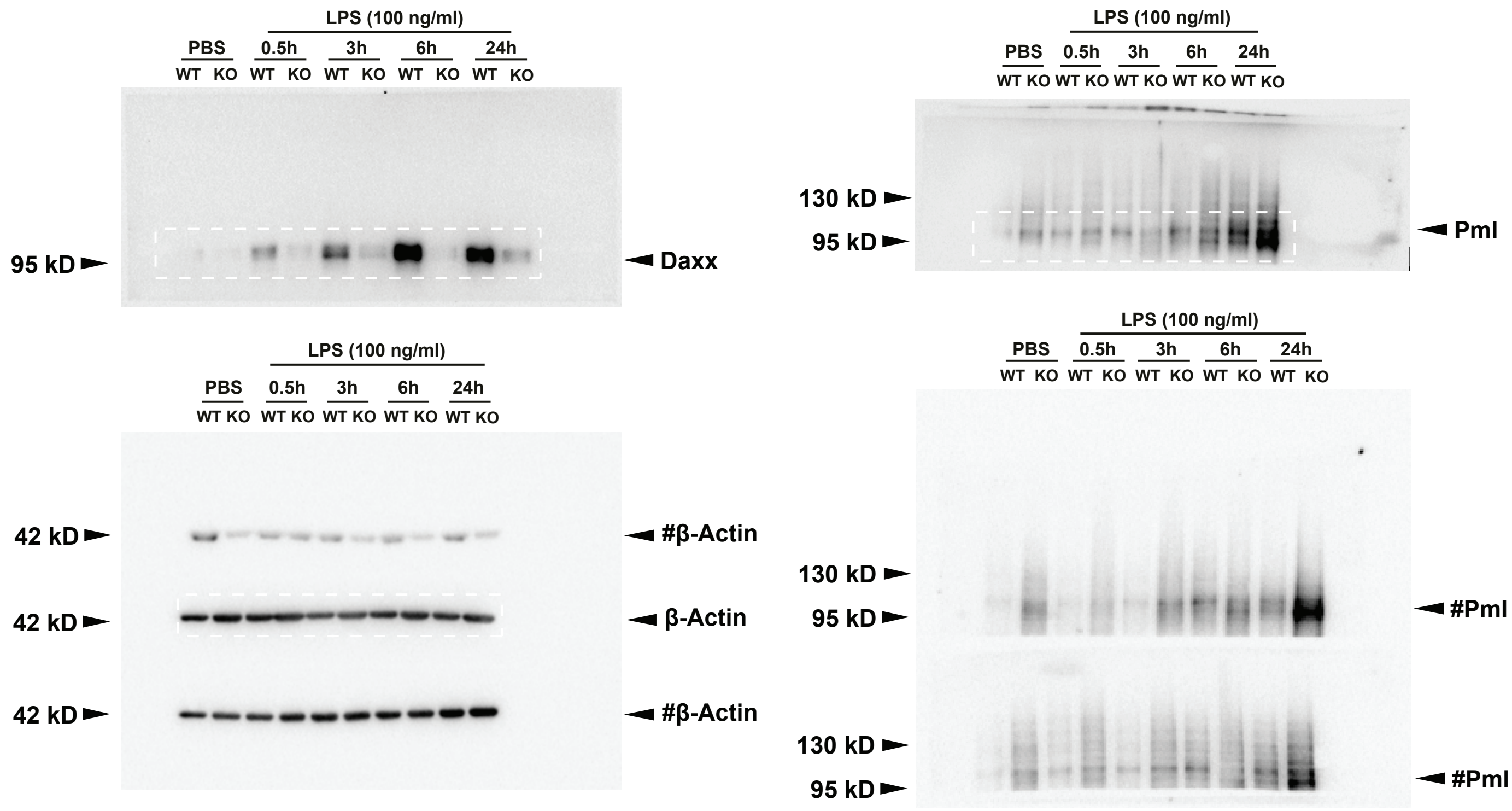

# additional panels shown above not in manuscript

Extended Data Fig. 10c

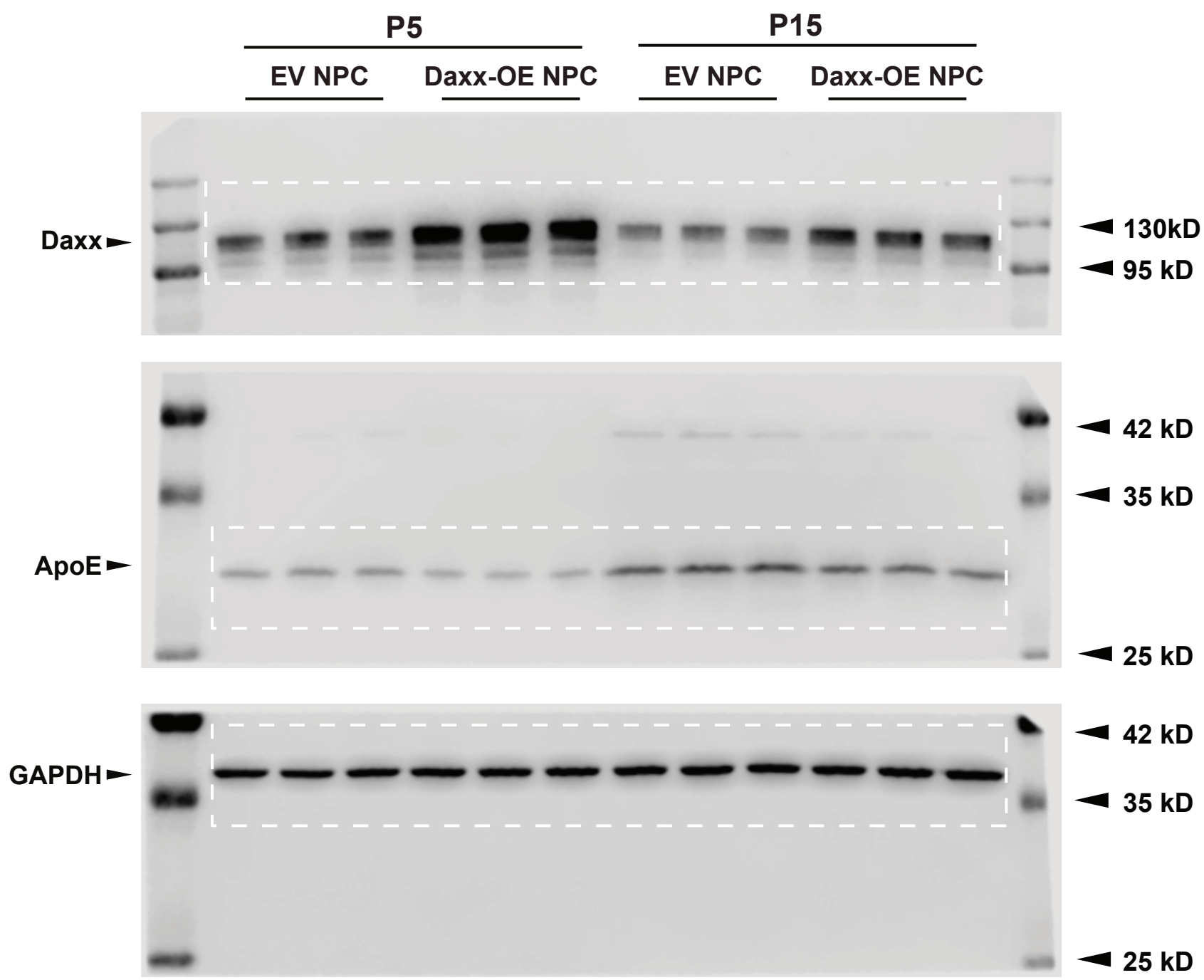

Note: Dashed white lines identify cropped areas
