# Supplementary material for: Microglia activation by derepression of endogenous retroviruses drives inflammation and cellular senescence
Source: Nat Neurosci. 2026 Aug 17;29(10):2480–98. doi: 10.1038/s41593-026-02404-y (PMC13633122; doi:10.1038/s41593-026-02404-y)
Supplement: Supplementary file 3 [file 41593_2026_2404_MOESM2_ESM.pdf]

nature portfolio

Corresponding author(s): Prof. Paolo Salomoni

Last updated by author(s): Jun 23, 2026

Reporting Summary

Nature Portfolio wishes to improve the reproducibility of the work that we publish. This form provides structure for consistency and transparency in reporting. For further information on Nature Portfolio policies, see our Editorial Policies and the Editorial Policy Checklist.

Statistics

For all statistical analyses, confirm that the following items are present in the figure legend, table legend, main text, or Methods section.

n/a	Confirmed	
☐	☒	The exact sample size (*n*) for each experimental group/condition, given as a discrete number and unit of measurement
☐	☒	A statement on whether measurements were taken from distinct samples or whether the same sample was measured repeatedly
☐	☒	The statistical test(s) used AND whether they are one- or two-sided<br>*Only common tests should be described solely by name; describe more complex techniques in the Methods section.*
☐	☒	A description of all covariates tested
☐	☒	A description of any assumptions or corrections, such as tests of normality and adjustment for multiple comparisons
☐	☒	A full description of the statistical parameters including central tendency (e.g. means) or other basic estimates (e.g. regression coefficient) AND variation (e.g. standard deviation) or associated estimates of uncertainty (e.g. confidence intervals)
☐	☒	For null hypothesis testing, the test statistic (e.g. *F*, *t*, *r*) with confidence intervals, effect sizes, degrees of freedom and *P* value noted<br>*Give P values as exact values whenever suitable.*
☒	☐	For Bayesian analysis, information on the choice of priors and Markov chain Monte Carlo settings
☒	☐	For hierarchical and complex designs, identification of the appropriate level for tests and full reporting of outcomes
☐	☒	Estimates of effect sizes (e.g. Cohen's *d*, Pearson's *r*), indicating how they were calculated

Our web collection on statistics for biologists contains articles on many of the points above.

Software and code

Policy information about availability of computer code

Data collection	Imaging data was collected using Zeiss 700 or Zeiss 900.<br>Blots was imaged using LICOR OXF-2068 or ChemiDoc XRS+ system<br>bulk RNA-seq, ATAC-seq, CUT&Tag and scRNA-seq data were collected by Illumina NexSeq 500, Illumina NovaSeq 6000, Illumina NovaSeq X Plus system.
Data analysis	All graphical and statistical analyses were performed using: ImageJ (v1.5e), ImageJ (v2.14), Imaris v9.8 software, Prism9 software (Graphpad Software,USA) and microsoft Excel.<br>R package Seurat (v.1.1), Bcl2fastq2 (V2.20), STAR (v. 2.6.1b), STAR (v2.7.1), Dropseq-tools (v. 2.0.0), Bioconductor package celda (1.10.0), monocle3 (1.0.0), DESeq2 (ver. 1.34.0), TrimGalore(v. 0.6.10), Subread (v. 2.0.3), sklearn python package, ChIPpeakAnno (ver. 3.28.1), atacseq pipeline (ver. 1.2.2), ChIPseeker R package (v. 1.26.2), SQuIRE pipeline (ver. 0.9.9.92), clusterProfiler R package (v. 3.18.1), deeptools (v. 3.5.1) package , BWA-MEM (v0.7.8-r455), SAMtools (v1.11), deepTools (v3.5.3), HOMER (v5.1) were used for bio-informatic analysis.<br>Please see Methods section in manuscript for further detail.

For manuscripts utilizing custom algorithms or software that are central to the research but not yet described in published literature, software must be made available to editors and reviewers. We strongly encourage code deposition in a community repository (e.g. GitHub). See the Nature Portfolio guidelines for submitting code & software for further information.

Data

Policy information about availability of data

All manuscripts must include a data availability statement. This statement should provide the following information, where applicable:

- Accession codes, unique identifiers, or web links for publicly available datasets
- A description of any restrictions on data availability
- For clinical datasets or third party data, please ensure that the statement adheres to our policy

ll original uncropped western blots in this study are provided in the Source Data as Source Data Raw blots. The raw and processed bulk RNA-Seq, ATAC-seq, scRNA-seq and CUT&Tag data are deposited on GEO (GSE255329, GSE276607, GSE267028, GSE272828,GSE309782). The following datasets containing expression analysis from mice microglia and neuronal cells were re-analyzed for this study: GEO accession number GSE121654 (Hammond et al.), GSE207932 (Li et al), GSE298386 (Li et al), GSE79819 (Matcovitch-Natan), GSE52564 (Zhang et al.), E-MTAB-5077 (Buttgereit et al.), GSE226092 (Fixen et al.), GSE119309 (Gerber et al), GSE150169 (Zhan etal.). Other data generated during and/or analyzed during the current study are available from the corresponding author on reasonable request.

Research involving human participants, their data, or biological material

Policy information about studies with human participants or human data. See also policy information about sex, gender (identity/presentation), and sexual orientation and race, ethnicity and racism.

Reporting on sex and gender	Sex was used as a covariate in all statistical models.
Reporting on race, ethnicity, or other socially relevant groupings	The human study population was overwhelmingly of European descent (>95%). Therefore, meaningful race or ethnicity-stratified analyses were not possible. Moreover, based on the animal findings, it was not expected that race, ethnicity or other social groupings would act as modifiers of the relation between DAXX and RTE expression levels.
Population characteristics	The current analysis was conducted on a total of 2922 participants of the Rhineland Study. Age, sex and smoking status data were derived from self-reports.
Recruitment	The human data were derived from the Rhineland Study, an ongoing community-based cohort study in Bonn, Germany. All inhabitants aged 30 years and above of two geographically defined areas were invited to participate in the Rhineland Study. The sole exclusion criterion for participation in the Rhineland Study was insufficient command of the German language to provide informed consent. For the present study we included the subset of all Rhineland Study participants in whom transcriptomic data were available.
Ethics oversight	The Rhineland Study was approved by the ethics committee of the University of Bonn, Medical Faculty. The Rhineland Study is carried out in accordance with the recommendations of the International Conference on Harmonization (ICH) Good Clinical Practice (GCP) standards (ICH-GCP). Written informed consent was obtained from all participants in accordance with the Declaration of Helsinki.

Note that full information on the approval of the study protocol must also be provided in the manuscript.

Field-specific reporting

Please select the one below that is the best fit for your research. If you are not sure, read the appropriate sections before making your selection.

☒ Life sciences ☐ Behavioural & social sciences ☐ Ecological, evolutionary & environmental sciences

For a reference copy of the document with all sections, see nature.com/documents/nr-reporting-summary-flat.pdf

Life sciences study design

All studies must disclose on these points even when the disclosure is negative.

Sample size	No statistical method was used to predetermine sample size. Sample size was chosen based on our previous studies using the same types of assays (Gerber et al. 2021, Nat Cell Biol.).<br>In vitro studies used a standard of at least N=3 independent experiments were always used.<br>Histology were performed on at least 3 mice per group.<br>Sample sizes for behavioral experiments were determined based on the current standard, as the minimum amount of mice required to detect significance with an alpha rate set at 0.05 in a standardly powered experiment.
Data exclusions	No data was excluded from the analysis.
Replication	Experiments run on the same day always included control and knock-out mice. Most experiments were performed on at least 3 independent trails. The attempts of replication were successful and no experiment was excluded. All samples and mice were handled following the same protocols. The number of biological replicates for Rose-CreERT;H3.3Afl/fl,H3.3B-/- mouse is limited to 2 mice per genotype, since tissues from this mouse line were obtained from our collaborators at Cornell, who at the time had limited animal availability within the timeframe of our study. Data from enzymatic dissociation-induced artificial genes were part of a control experiment suggested by the reviewer, which was based on a limited number of biological replicates due to the turnaround time of our revision. Bulk RNAseq at 8wpi were run with 2 biological

replicates per genotype/condition, and scRNAseq data were run with 2 biological replicates for 3dpi WT, 1wpi Daxx KO and 8wpi, and 1 replicate for 3dpi Daxx KO and 1wpi WT; CUT&Tag data were run with 3 biological replicates for H3K9me3 Daxx KO and H3.3 WT, and 2 biological replicates for H3K9me3 WT and H3.3 Daxx KO.

Randomization

Sample allocation was random and mice for experiments were randomly chosen based on availability of the right genotypes.

Blinding

During all animal studies, animals are labeled by identification number and genotype is unknown to experimenter during testing. For all in vitro studies, due to the relatively small number of biological replicates in each experiment, both control and KO groups are known to the experimenter. For all other analyses, the experiments were blinded to the genotype or treatment until completion of statistical analyses.

Reporting for specific materials, systems and methods

We require information from authors about some types of materials, experimental systems and methods used in many studies. Here, indicate whether each material, system or method listed is relevant to your study. If you are not sure if a list item applies to your research, read the appropriate section before selecting a response.

Materials & experimental systems

n/a	Involved in the study	
☐	☒	Antibodies
☒	☐	Eukaryotic cell lines
☒	☐	Palaeontology and archaeology
☐	☒	Animals and other organisms
☒	☐	Clinical data
☒	☐	Dual use research of concern
☒	☐	Plants

Methods

n/a	Involved in the study	
☒	☐	ChIP-seq
☒	☐	Flow cytometry
☒	☐	MRI-based neuroimaging

Antibodies

Antibodies used

Mouse monoclonal anti-Beta Amyloid (1:1000, 6E10, Covance Cat#SIG-39300)
Mouse monoclonal anti-β-actin (1:1000, Sigma-Aldrich Cat#A1978)
Rabbit monoclonal anti-ApoE (1:1000, Abcam, Cat#AB183597)
Mouse monoclonal anti-Bassoon (1:800, Enzo Cat#ADI-VAM-PS003-F)
Rat monoclonal anti-CD68 (1:200, Biolegend, Cat#137001; FA-11)
Rabbit polyclonal anti-Cleaved Caspase-3 (Cell Signaling, Cat#9661)
Rabbit polyclonal anti-Daxx (1:300, Santa Cruz Biotechnology, Cat#sc-7152)
Rabbit polyclonal anti-Daxx (1:1000, Abcam, Cat# Ab32140)
Chicken polyclonal anti-GFAP (1:300, Abcam, Cat#ab4674)
Rabbit polyclonal anti-γH2AX (1:1000, Cell Signaling, Cat#9718)
Rabbit polyclonal anti-H2AX (1:1000, Cell Signaling, Cat#2595)
Rabbit polyclonal anti-H3.3 (1:20, Millipore Cat#09-898)
Rabbit polyclonal anti-H3k9me3 (1:50, Active Motif, Cat#39062)
Chicken polyclonal anti-Homer1 (1:500, Synaptic Systems, Cat#160006)
Guinea pigs polyclonal anti-Iba1 (1:500, Synaptic Systems, Cat#234004)
Rabbit monoclonal anti-Ki67 (1:300, Abcam, Cat#ab16667)
Rat monoclonal anti-Lamp1 (1:300, Abcam Cat#ab25245)
Rat monoclonal anti-MBP (1:300, Abcam Cat#ab7349)
Rabbit polyclonal anti-MDA5 (1:1000, Thermo Fisher Scientific, Cat#PA5-20337)
Rabbit polyclonal anti-Mavs (1:1000, Abcam, Cat# Ab31334)
Chicken polyclonal anti-NeuN (1:500, Abcam, Cat#ab134014)
Mouse monoclonal anti-Pml (1:1000, Sigma-Aldrich, Cat#05-718)
Rabbit polyclonal anti-p53 (1:1000, Leica Biosystems, Cat#NCL-L-P53-CM5P)
Rabbit polyclonal anti-p-p53 (1:1000, Cell Signaling, Cat#9284)
Rabbit polyclonal anti-PSD95 (1:500, Synaptic Systems, Cat#124003)
Rabbit polyclonal anti-Stat1 (1:1000, Cell Signaling, Cat#9172S)
Rabbit polyclonal anti-p-Stat1 (1:1000, Cell Signaling, Cat#9167S)
Mouse monoclonal anti-SMI31 (1:200, Biolegend, Cat#801602)
Rabbit monoclonal anti-Sox9 (1:300, Abcam, Cat#ab185230)
Rabbit polyclonal anti-synaptophysin (1:500, Cell Signaling, Cat#4329)
Rabbit monoclonal anti-Trim28 (1:1000, Abcam Cat#ab10484)

Validation

All antibodies used are commercially available antibodies and detailed validation information of each antibody can be found from the manufacturer.

Animals and other research organisms

Policy information about studies involving animals; ARRIVE guidelines recommended for reporting animal research, and Sex and Gender in Research

Laboratory animals	C57BL/6 Daxx-floxed mice and PML-floxed mice were generated by Taconic Artemis (Cologne, Germany). IFNAR KO mice were purchsed from The Jackson Laboratory (stock no. 028228). Floxed Daxx mice were crossed with C57BL/6J Cx3cr1CreERT2 mice (JAX 020940), C57BL/6J Rosa26CreERT2 mice (JAX 008463). C57BL/6N floxed Hira mice were crossed with C57BL/6J Cx3cr1CreERT2 mice (JAX 020940). Floxed H3.3A,H3.3B-/- mice were crossed with C57BL/6J Rosa26CreERT2 mice (JAX 008463). To induce Cre activation, Rosa26-CreERT2 or Cx3cr1-CreERT2 positive mice at 6-7-week-old age was administrated with 80 mg/kg/day tamoxifen (T5648, Sigma-Aldrich) via oral gavage for five consecutive days. For polyinosinic-polycytidylic acid stimulation, mice were injected by i.p. with 300 µg of polyinosinic-polycytidylic acid (Poly I:C) for 5 or 12 hours. Mice were maintained on a 12-hour light/dark cycle with ad libitum access to regular chow food (ssniff Spezialdiäten GmbH).
Wild animals	The study did not involve wild animals
Reporting on sex	Mice of both genders were used in this study. Data of in vitro microglia or BMDM isolated from both male and female were pooled. Behavior tests were performed only on male mice.
Field-collected samples	The study did not involved field-collected samples.
Ethics oversight	All experimental procedures were approved by Landesamt für Verbraucherschutz und Ernährung Nordrhein-Westfalen (LAVE) (formely Landesamt für Natur, Umwelt und Verbraucherschutz Nordrhein-Westfalen (LANUV)) under protocols 81-02.04.2016.A486, 81-02.04.2020.A346 and 81-02.04.2021.A019.

Note that full information on the approval of the study protocol must also be provided in the manuscript.

Plants

Seed stocks	This study did not involve plants
Novel plant genotypes	This study did not involve plants
Authentication	This study did not involve plants
